# Homogeneous accretion of the Earth in the inner Solar System

**DOI:** 10.1038/s41550-026-02824-7

**Published:** 2026-03-27

**Authors:** Paolo A. Sossi, Dan J. Bower

**Affiliations:** Institute of Geochemistry and Petrology, Department of Earth and Planetary Sciences, ETH Zürich, Zurich, Switzerland

**Keywords:** Geochemistry, Early solar system, Inner planets

## Abstract

Meteorites are classified as either non-carbonaceous or carbonaceous, representing bodies that are likely to have formed in the inner or outer Solar System, respectively. Despite its location in the inner Solar System, the Earth is thought to contain either minor (~6%) or substantial amounts (~40%) of outer Solar System material. However, because neither interpretation leverages variations among multiple isotopic systems simultaneously, Earth’s provenance remains equivocal. Here we examine variations in ten nucleosynthetic isotope anomalies among planets and meteorite parent bodies to show that the linear extension of an array defined by non-carbonaceous bodies in any two isotopic anomalies always intersects the observed isotopic composition of the bulk silicate Earth to within 1 standard deviation. The Earth therefore formed exclusively from inner Solar System material whose composition did not vary over the course of accretion and was, on average, unlike that of any chondrite. Extension of the non-carbonaceous array yields isotopic compositions for Mercury and Venus that are more extreme than for Earth, implying a spatial or temporal gradient during the formation of the terrestrial planets.

## Abstract (web summary)

Earth’s origin is debated. Meteorites formed in the inner and outer Solar System, but Earth represents an endmember, inner Solar System source alone. Venus and Mercury are predicted to be more extreme, implying a gradient in the inner Solar System.

## Main

The identification of two, distinct populations of meteorites from their mass-independent isotopic compositions^1^, the ‘isotopic dichotomy’, has precipitated a revolution in our understanding of the provenance of planetary materials and, in turn, the spatio-temporal evolution of the early Solar System^2–5^. These two populations, named non-carbonaceous (NC) and carbonaceous (CC), preserve small differences in the abundances of isotopes produced by different nucleosynthetic processes, often expressed as their parts-per-ten-thousand deviation from a standard,

1 $${\varepsilon}^{i}X=\left(\frac{\left({i\atop}\!X/{j\atop}\!X\right)_{\mathrm{reservoir}}}{\left({i\atop}\!X/{j\atop}\!X\right)_{\mathrm{standard}}}-1\right)\times 10\,000$$

where *i* and *j* are the isotopic masses and *X* is the element. Isotopic anomalies in *ε*^54^Cr and *ε*^50^Ti, upon which the dichotomy was first mooted, are roughly linearly correlated among NC bodies, with CI chondrites, a CC body, falling close to^6^ or on an extension of this correlation^7^. Because the composition of the Earth, as inferred from that of its mantle, the bulk silicate Earth (BSE), is intermediate between the NC group and CI chondrites, it can be made from a mixture of CI chondrites and any combination of NC bodies in *ε*^54^Cr–*ε*^50^Ti space. The choice of NC body that represents the mixing endmember influences the apparent CI fraction that comprises the BSE. If enstatite chondrites (ECs) are chosen, then the BSE would be made of ~94% EC-like- and ~6% CI-like matter^8–11^ whereas if ureilites are chosen, then the BSE must contain 40% CI-like material^3,12,13^. Because CIs are thought to have originated in the outer Solar System, high CI fractions are cited as evidence of substantial accretion of sunward-drifting pebbles to the Earth^12–14^, whereas low CI fractions are posited to reflect its classical, oligarchic growth from planetesimals largely within the inner Solar System^10,11^.

Subsequently, the discovery of the dichotomy in two isotope ratios of a heavy element, Mo, revealed that the BSE is an s-process-enriched endmember among all extant meteorite groups^15^. This property was shown to extend to other heavy element nuclides (Zr, Ru and Nd^16–18^), which means that the Earth cannot reflect mixtures of the known NC and CC bodies alone. Instead, accretion of a third, ‘missing’ component to the Earth^10^ or preferential evaporation of s-process-depleted phases in the envelope of the proto-Earth^12^ have been proposed as possible solutions.

The acceptance, by some workers, of a higher apparent CC contribution to the BSE budget of Mo (~40 ± 20%)^11,19,20^ than that recorded in Ti and Cr, is taken as support of an earlier notion^21,22^ that the Earth accreted heterogeneously^8^. Proxies that are not nucleosynthetic in origin^21,23–25^ have been cited in support of a CC contribution to the BSE, particularly in the latter stages of its accretion. More recent models grounded in nucleosynthetic isotope variations indicate that Earth initially accreted NC-rich material and became more CC like over time, before the addition of an NC-rich late veneer^9,11,17^. This scenario requires that the growing Earth’s mantle equilibrates imperfectly with its core, such that isotopic anomalies in the BSE of more siderophile elements, namely, Mo, reflect later stages of Earth’s formation than do lithophile elements, such as Ti^8^. In addition to Mo, ~30% of the BSE’s Zn budget is interpreted to have been delivered by CC material^26–28^. Differences in Ru isotopic compositions between modern-day and Archean rocks also indicate a degree of heterogeneous accretion, although this may reflect diversity in the compositions of NC bodies rather than a CC contribution^29,30^. By contrast, a reassessment of Mo isotopic anomalies in the BSE indicates that they are consistent with an NC origin alone within present analytical uncertainties^31,32^, which, if correct, casts doubt on the necessity for the heterogeneous accretion of CC material.

All of the aforementioned models have been motivated by the perceived necessity to make Earth, at least partly, from some mixture of existing meteorite families or their components. However, these interpretations: (1) are based on only a subset of the measured isotopic anomalies^11,33^; (2) implicitly assume heterogeneous accretion^9^; and/or (3) invoke additional factors, namely, envelope processing^12^ or missing components^10^ to extend or modify the isotopic range in meteorites. Here we examine the composition of the BSE in relation to other meteorite groups in terms of ten different isotopic anomalies by means of a probabilistic approach that interrogates the data independently of any accretion model for the Earth.

## Results

### PCA and B-LFA

We quantified the isotopic relationship between the BSE and other planetary materials (hereinafter ‘reservoirs’) using the means and standard errors of a range of isotopic anomalies (*ε*^48^Ca, *ε*^50^Ti, *ε*^54^Cr, *ε*^54^Fe, *ε*^64^Ni, *ε*^66^Zn, *ε*^94^Mo, *ε*^95^Mo, *ε*^96^Zr and *ε*^100^Ru) for each reservoir, tabulated from the public OriginsLab database^9^ (Methods and Supplementary Section 1.1). The isotopic compositions of *ε*^94^Mo and *ε*^95^Mo in the BSE were recalculated, taking into account the most recent estimates^20,32^, which yielded *ε*^94^Mo = 0.00 ± 0.05 and *ε*^95^Mo = 0.03 ± 0.03 (Supplementary Fig. 1). We used the present-day *ε*^100^Ru of the BSE^17,29^. We excluded Δ^17^O, as variations are thought not to be primarily nucleosynthetic^34^. Data for Si isotopes were also neglected, owing to uncertainties in correcting for mass-dependent isotopic fractionation^9,12^.

The isotopic systems were classified based on either their: (1) cosmochemical character; or (2) nucleosynthetic origin. In the first scheme, the iron-loving, siderophile elements (Fe, Ni, Mo, Ru) were distinguished from the silicate-loving, lithophile elements (Ca, Ti, Cr, Zr and Zn), to identify any differences in the isotopic provenance of material brought to the BSE over time^8^. The second scheme separated iron-peak elements (Ca, Ti, Cr, Fe, Ni and Zn) from heavy elements (Mo, Zr and Ru), whose constituent nuclides have distinct nucleosynthetic heritage^35^, enabling detection of different stellar sources.

All isotope systems, in addition to each subset, were subjected to dimensionality reduction that combines principal component analysis (PCA) as a prior for Bayesian latent factor analysis (B-LFA; Methods). The analysis required data for the ten isotopic systems in each reservoir, limiting the analysis to the ureilites (9/10 ratios), Vesta group (9/10 ratios), L, LL (9/10 ratios), H, EL, EH (9/10 ratios), CI, CR, CV, CO, CM, Mars (9/10 ratios) and the BSE. The few missing isotopic ratios were inserted as synthetic priors based on correlations among meteorite groups (Supplementary Section 2.1). Reservoir mean values are weighted averages of samples whose isotopic compositions fall within uncertainty of one another. The Vesta group is an exception; it is a composite reservoir comprising samples from Vesta, angrites, acapulcoites, lodranites, mesosiderites and main group pallasites, based on criteria defined in Supplementary Section 2.1. The dataset used for the analysis is given in Supplementary Table 1.

**Fig. 1:**
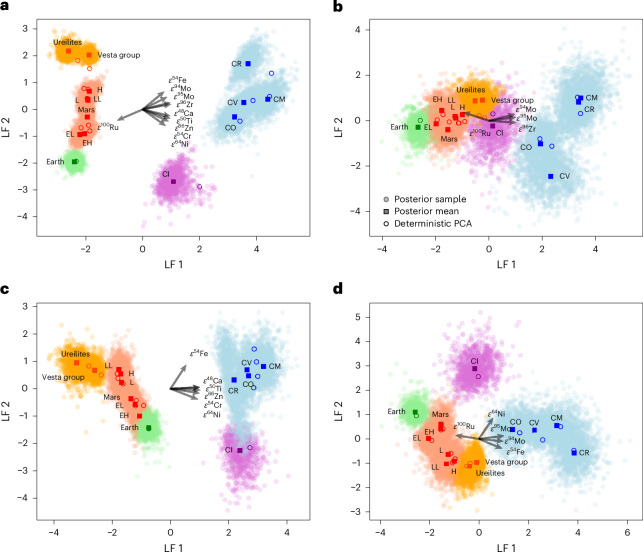
Results of the B-LFA and deterministic PCA. **a**–**d**, All elements (**a**), heavy elements (**b**), iron-peak elements (**c**) and siderophile elements (**d**) for all 14 reservoirs. The CI reservoir is clearly separated from the other reservoirs of the CC group. The NC group can also be divided into two subgroups; the ureilities and the Vesta group represent a population distinct from those of the group consisting of H, L, LL (OCs), Mars, EH and EL (ECs), collectively the OC–EC subgroup. The BSE is an extension of the OC–EC subgroup for all element groups, regardless of their chemical or nucleosynthetic affinity. Open circles are computed from deterministic PCA. Transparent circles show the 10^4^ samples from the posterior distribution and filled squares are the means of the B-LFA posterior. The arrows denote the loading vector for each isotopic ratio to the LFs (for example, a reservoir with high *ε*^100^Ru will plot to lower values of LF1 at near-constant LF2 in **d**).

Based on the means of the isotopic ratios for each reservoir, PCA was performed to determine the latent factors (LFs) that maximize the combined variance along orthogonal axes, ordered by the percentage of the variance they explain (LF1 and LF2; open circles, Fig. 1). The PCA is used as the prior distribution for the LFs and loading matrix of the B-LFA. In the B-LFA, the likelihood function represents the probability of the observed data (that is, the ten isotopic systems in the original, higher-dimensional space) given the LFs. Posterior distributions of LFs for each reservoir (filled circles, Fig. 1) combine the prior PCA estimate with the evidence provided by the observed data and their uncertainties. The B-LFA relaxes the constraint of variance orthogonality imposed by the PCA and, in so doing, provides more accurate estimates of the means and uncertainties of each reservoir than does PCA alone.

The first two principal components (PC1 and PC2) explain 90–97% of the variance in the data, depending on the chosen subset of isotopic anomalies (Supplementary Table 2), substantiating the two-dimensional representation in Fig. 1. Striking is the observation that the NC reservoirs (orange and red shades) are clearly separated from the CC reservoirs (blue and purple shades) by virtue of their distinct LF1 values when all isotopic ratios are considered (Fig. 1a), a feature that holds for iron-peak elements (Fig. 1c) and lithophile elements (Supplementary Fig. 3), but is less clearly pronounced among the heavy elements and siderophile elements (Fig. 1b,d). In all cases, CI chondrites are intermediate to, but distinct from, the NC and CC groups in LF1–LF2 space, providing further support for their status as a singular reservoir^4,9,36–38^.

The NC reservoirs occupy a similar location in LF1–LF2 space relative to one another, independent of the subset of isotopic anomalies chosen; the ureilites characterize one extremum of the group, whereas the BSE defines the other. This observation, already evident in *ε*^54^Cr–*ε*^50^Ti space^1^, therefore holds across all isotopic systems. Although the relationship between the NC reservoirs has been approximated as linear in *ε–ε* space^10,18^, it is evident from Fig. 1 that the Vesta group and ureilites (orange points) diverge from other NC reservoirs (see also Supplementary Fig. 4, ref. ^5^). An extension of the line passing through the remaining NC reservoirs—OCs (H, L, LL), Mars and the ECs (EH, EL), hereinafter the ‘OC–EC’ subgroup—always intersects the BSE, within uncertainty, for all elements (Fig. 1a) and any subset thereof (Fig. 1b–d). By contrast, this same linear extension never intersects the isotopic composition of CIs. Therefore, the notion that the BSE can be produced by a mixture of an NC body, namely, ECs^11^ or ureilites^3^ and CI chondrites, an interpretation permitted on the basis of *ε*^54^Cr–*ε*^50^Ti variations alone^7,39^, is precluded when all isotopic systems are considered together.

The location of any reservoir, A, in LF1–LF2 space relative to another reservoir, B, can be quantified using the signed Euclidean distance, ${d}_{{\rm{A}}-{\rm{B}}}^{{\rm{s}}}$, between them, where

2 $${d}_{{\mathrm{A}}-{\mathrm{B}}}^{{\mathrm{s}}}=\sqrt{{({\mathrm{LF}}{1}_{{\mathrm{A}}}-{\mathrm{LF}}{1}_{{\mathrm{B}}})}^{2}+{({\mathrm{LF}}{2}_{{\mathrm{A}}}-{\mathrm{LF}}{2}_{{\mathrm{B}}})}^{2}}\sim{\mathrm{s}}{\mathrm{i}}{\mathrm{g}}{\mathrm{n}}({{\mathbf{a}}}_{{\mathrm{B}}}\times {{\mathbf{v}}}_{{\mathrm{A}}-{\mathrm{B}}})$$

and the term in brackets is the cross product of ${{\bf{a}}}_{{\rm{B}}}$, the reference axis pointing away from body B and ${{\bf{v}}}_{{\rm{A}}-{\rm{B}}}$, the vector from B to A, ensuring that ${d}_{{\rm{A}}-{\rm{B}}}^{{\rm{s}}}$ can be positive or negative. To render ${d}_{{\rm{A}}-{\rm{B}}}^{{\rm{s}}}$ independent of the spread in the subset of isotopic anomalies chosen for the B-LFA, ${d}_{{\rm{A}}-{\rm{B}}}^{{\rm{s}}}$ is measured relative to that between a third, fixed reference, C and B, ${d}_{{\rm{C}}-{\rm{B}}}^{{\rm{s}}}$. The ratio;

3 $${R}_{{\rm{A}}}={d}_{{\rm{A}}-{\rm{B}}}^{{\rm{s}}}/{d}_{{\rm{C}}-{\rm{B}}}^{{\rm{s}}}$$

defines a dimensionless scale-invariant signed Euclidean distance (hereinafter ‘isotopic Euclidean distance’). For B = BSE (hence *R*_BSE_ = 0) and C = OC (hence *R*_OC_ = 1), then for A = EC, *R*_EC_ yields a constant factor, 0.43 ± 0.10 across all isotopic anomalies and subsets thereof (Fig. 1a–d and Supplementary Table 4).

Therefore, the BSE, as the silicate portion of a differentiated body, bears a similar isotopic relationship to the undifferentiated, chondritic bodies that constitute the OC–EC subgroup of the NC trend, irrespective of the siderophility or nucleosynthetic origin of the element. Two corollaries emerge from this observation: (1) Earth formed, on average, from NC material that lies on a linear extension of the trend defined by the OC–EC subgroup; and (2) the composition of this NC material is independent of the isotopic ratio used to infer it. Consequently, the isotopic composition of the BSE is intrinsic to the Earth and cannot reflect a mixture of existing planetary materials alone, as indicated for isotopic anomalies in heavy elements^10,29,30^ and potentially K isotopes^40^. However, unlike in the aforementioned studies, we propose that the endmember status of the BSE among the NC bodies applies equally across each of the ten isotopic systems considered here.

### Multivariate linear regression

To test hypotheses (1) and (2), above, we perform linear regressions using the York method^41^ between the mean of the isotopic anomalies (*x*_A_, *y*_A_ and their standard errors ${\sigma }_{{x}_{{\rm{A}}}}$, ${\sigma }_{{y}_{{\rm{A}}}}$) measured in all isotopic systems (for example, *ε*^50^Ti versus *ε*^96^Zr) among reservoirs, A, of the OC–EC subgroup (see Supplementary Section 3.1, Supplementary Fig. 5 and Supplementary Table 5 for the complete list of reservoirs). We fix the measured isotopic anomaly in one element, the predictor, *x*_BSE_ in the BSE to compute the isotopic compositions of the remaining nine elements, ${y}_{\mathrm{BSE}}^{1},\,{y}_{\mathrm{BSE}}^{2}\,\ldots \,{y}_{\mathrm{BSE}}^{n}$ (*n* = 9), from the linear correlations between them and the predictor. Standard errors on the predicted means, ${y}_{\mathrm{BSE}}^{n}\pm {\sigma }_{{y}_{\mathrm{BSE}}^{n}}$, are calculated by Monte Carlo simulation with 10^4^ samples from normal distributions about the means in the slope (*a* ± *σ*_*a*_) and intercept (*b* ± *σ*_*b*_) according to their covariance matrix for each of the linear correlations, and in the measurement of the predictor (${x}_{\mathrm{BSE}}\pm {\sigma }_{{x}_{\mathrm{BSE}}}$). This procedure is repeated by cycling through each of the ten isotopic ratios as the predictor to give the error-weighted mean and standard deviation for each isotopic ratio, ${\widehat{y}}_{\mathrm{BSE}}^{n}\pm {\sigma }_{{\widehat{y}}_{\mathrm{BSE}}^{n}}$. The analysis is described in the Methods and regression and goodness-of-fit metrics are shown in Supplementary Figs. 6 and 7.

**Fig. 2:**
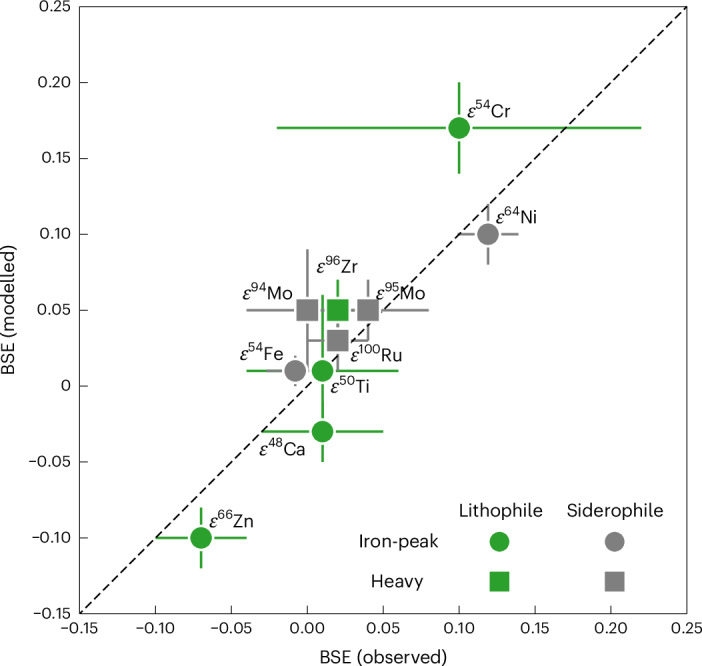
Comparison of the observed and modelled isotopic composition of the BSE. The observed mean isotopic anomalies in the BSE and their standard errors are shown against those predicted by multivariate linear regression, calculated by assuming that the BSE lies on an extension of the NC array defined by the OC–EC subgroup, ${\widehat{y}}_{\mathrm{BSE}}^{n}\pm {\sigma }_{{\widehat{y}}_{\mathrm{BSE}}^{n}}$. Isotopic anomalies are grouped according to the geochemical character and nucleosynthetic origin of the nuclides. The dashed line represents the 1:1 line. All modelled isotopic anomalies fall within 1*σ* of their observed values.

The error-weighted mean of any given predicted isotopic ratio computed across all predictors, ${\widehat{y}}_{\mathrm{BSE}}^{n}\pm {\sigma }_{{\widehat{y}}_{\mathrm{BSE}}^{n}}$, falls within one standard deviation (mean across all groups is 0.65 ± 0.55) of the corresponding measured isotopic ratio, ${x}_{\mathrm{BSE}}^{n}\pm {\sigma }_{{x}_{\mathrm{BSE}}^{n}}$ (Fig. 2 and Supplementary Tables 6 and 7). Moreover, the composition of the BSE is predicted equally well using linear regressions between any two combinations of lithophile, siderophile, iron-peak and heavy elements according to their *Z*-scores (Supplementary Table 8 and Supplementary Fig. 8). As groups, the mean absolute *Z*-scores per group range between 0.73 ± 0.18 between heavy element–iron-peak pairs and 0.57 ± 0.14 between siderophile–siderophile element pairs. This is evident in Fig. 2, where all element groups have isotopic compositions that adhere to the 1:1 line. All individual binary correlations in *ε–ε* space are shown in Supplementary Figs. 9 to 18. Figure 2 shows that the composition of the BSE, for each isotopic system, is entirely consistent with that of an NC body; specifically, an endmember of the OC–EC subgroup.

Two alternative hypotheses state that CI chondrites constitute ~6% (refs. ^9,11^) or ~40% (ref. ^12^) by mass of the Earth, with the remainder being made up of an NC body, either ECs^10,11^ or ureilites^3,12^, respectively. We show that both models are unnecessary for two reasons. First, binary mixing of CI chondrites and an NC body alone cannot reproduce the isotopic composition of heavy elements (*ε*^94^Mo, *ε*^95^Mo, *ε*^96^Zr and *ε*^100^Ru) in the BSE, given that it is an endmember among all known meteorites for these systems^15,18^. Second, although this shortfall can be remedied by a ‘missing NC-component’^9,10^ or envelope processing^12^ to produce an endmember rich in s-process isotopes, we show that this component, owing to the correlated nature of isotopic anomalies in the OC–EC subgroup of the NC group, must simultaneously be enriched in neutron-rich isotopes of iron-peak elements, obviating the need for a CI component.

To constrain the maximum permissible mass fraction of CI material, *f*CI, that remains consistent, within uncertainty, with the measured BSE composition, we perturbed it by adding CI chondrites. The isotopic compositions for each element in the resulting mixtures were calculated by: (1) weighting the CI and BSE isotopic compositions by their corresponding concentrations; and (2) assuming identical concentrations (Methods). The BSE’s depletion of siderophile elements makes their isotopic compositions, especially those of Mo and Ru, sensitive to CI chondrite addition, where *f*CI < 0.3 ± 0.1% or $< 0.{1}_{-0.03}^{+0.05}$%, respectively (Supplementary Fig. 19). For mixing between isotopically CI-like and BSE-like endmembers, but with equivalent concentrations of each element, *ε*^48^Ca constrains *f*CI to below 2 ± 0.2% in the BSE and, as a refractory lithophile element, in the bulk Earth (Supplementary Fig. 20). In either case, these are lower than previously inferred^10–12^. Because the isotopic compositions of *ε*^100^Ru and *ε*^48^Ca in other CC meteorites are more distinct from the BSE than are CI, the allowed *f*CC is correspondingly lower. Because these are maximum values, the isotopic composition of the BSE is consistent with lower amounts of CC material. Indeed, ~0.1%, ~0.3% and 1% of CI addition (by mass) would deliver the entire BSE complement of N, C and H, respectively^42^. Their mantle isotopic compositions, albeit mass dependent, are also consistent with an NC-like provenance, although their surface reservoirs are fractionated, complicating unambiguous identification of provenance^43^. Thus, the mass of carbonaceous material is likely to constitute less than ~0.1% of the BSE and <2% of the bulk Earth.

Excesses in the *ε*^100^Ru of Eoarchean (0.22 ± 0.04)^29^ and Hawaiian rocks (0.09 ± 0.03)^30^ relative to the modern-day BSE (0.02 ± 0.02) implies some heterogeneous accretion to the Earth, supported by *ε*^40^K data^40^. These authors suggest that such a signature could represent the pre-late-veneer mantle, which then accreted an s-process-depleted component (either NC or CC)^30,40^. However, because both siderophile and lithophile elements predict the modern-day BSE *ε*^100^Ru composition equally accurately (Fig. 2), the material responsible for determining the isotopic compositions of elements whose abundances cannot reflect the late veneer (for example, Ti or Ca), also set the Ru budget of the modern-day BSE. If the interpretation of refs. ^30^ and ^40^ is correct, then the observed consistency between the present-day *ε*^100^Ru of the BSE and those of lithophile element isotopic anomalies would be a coincidence. Alternatively, we propose that s-process heterogeneities preserved in *ε*^100^Ru (refs. ^29,30^) were derived from reservoir(s) whose contribution, on a mass basis, was negligible relative to that recorded in the contemporary BSE.

## Discussion

Our analysis shows that all elements, irrespective of their geochemical character or nucleosynthetic origin, record the same isotopic provenance in the BSE; that of an endmember among the NC group. The composition of the BSE is therefore defined as homogeneous with respect to isotopic anomalies. This observation permits two interpretations: (1) the Earth accreted material that, on average, maintained the same nucleosynthetic isotope composition during the time interval over which its core formed, ~34 Myr^44^ (see ref. ^30^ for an alternative interpretation); and/or (2) Earth could have accreted material with distinct nucleosynthetic isotope compositions (that is, heterogeneous accretion), but this heterogeneity must have been subsequently erased through perfect core–mantle equilibration. In both scenarios, the isotopic composition of any given siderophile element recorded in the BSE is equal to that of the bulk Earth.

That the isotopic compositions of bodies in the OC–EC subgroup (including the BSE) in any two elements of differing volatility (for example, Zn and Ti) define linear trends^18^ indicates that the variations are likely to have arisen by mixing between two endmember isotopic reservoirs, A and B, in which the concentrations of two elements, *i* and *j*, were subequal (that is, [*i*/*j*]_A_ ≈ [*i*/*j*]_B_). For a linear relationship to hold, option (2) is subject to the additional constraint that the degree of volatile depletion (for example, Zn/Ti ratio) was subequal among the different nucleosynthetic components that Earth accreted throughout its formation. Although possible^30,40^, these additional conditions make option (1) more probable.

Option (1) permits that the isotopic composition of Earth-forming material was either uniform or adhered to some distribution whose mean corresponds to the composition of the Earth. Because the OC–EC subgroup reservoirs are linearly related in *ε*–*ε* space, each individual reservoir could have sampled different portions of some continuous, yet imperfectly mixed isotopic distribution. Such isotopic continuity might have been achieved across either time (an evolving inner disk composition sampled sequentially by different NC bodies) or space (a spatial gradient in composition sampled contemporaneously^29,45^).

**Fig. 3:**
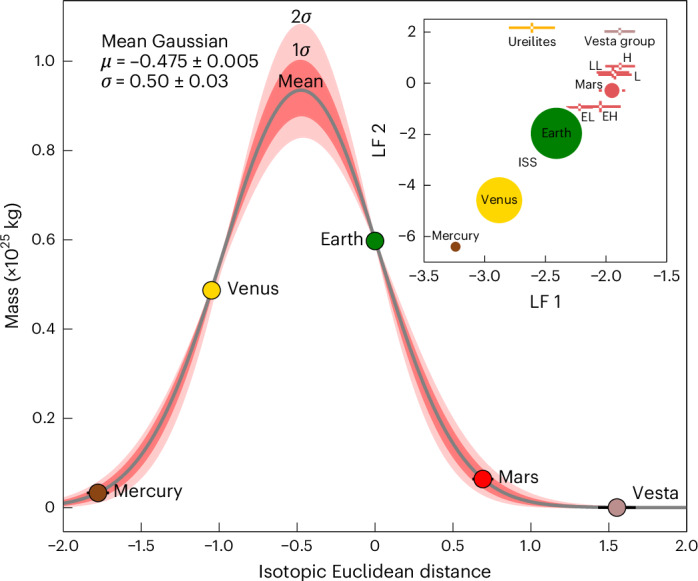
Prediction of the isotopic compositions of Venus and Mercury in isotopic Euclidean distance space. The mean and associated 1*σ* and 2*σ* confidence intervals around a mass-conserving Gaussian fit to the isotopic Euclidean distances (*R*_A_) of Mars and the Earth as a function of their mass. The confidence intervals were calculated by 10^4^ Monte Carlo simulations that account for the uncertainties in the isotopic compositions of three bodies across all isotopic anomalies in LF1–LF2 space shown in Fig. 1a. The isotopic Euclidean distances of Venus and Mercury are predicted based on their known masses. The peak of the distribution, *μ* = −0.475 ± 0.005 represents the mean isotopic composition of the inner Solar System, expressed in isotopic Euclidean distance from the Earth, and *σ* = 0.50 ± 0.03 represents its standard deviation and associated standard errors on both values. Inset: The locations of NC bodies in LF1–LF2 space across all elements (Fig. 1a), in which the size of the point is scaled proportional to the mass of the body. ISS denotes the mean composition of the inner Solar System.

The isotopic Euclidean distances (*R*_A_, Supplementary Table 4) between the Earth (0), Mars (0.69 ± 0.07) and Vesta (1.55 ± 0.07), are correlated with their semimajor axes (1.00 AU, 1.52 AU and 2.36 AU, respectively). Although such a gradient has been speculated to exist in individual *ε–ε* space, the coordinates of Mercury and Venus along this axis remain undetermined^29,45,46^. Because the present-day distribution of mass in the inner Solar System varies as a Gaussian function of heliocentric distance about 0.896 AU, we propose that *R*_A_ and planetary mass is also Gaussian. This model enables prediction of the isotopic compositions of Mercury and Venus by requiring that the integral of the Gaussian equals the combined masses of the four terrestrial planets. Figure 3 shows that the isotopic Euclidean distances, *R*_Venus_ and *R*_Mercury_, are $-1.0{5}_{-0.00}^{+0.01}$ and −1.78 ± 0.07, respectively, corresponding to coordinates (LF1, LF2) of (−2.88 ± 0.00, −4.58 ± 0.02)_Venus_ and (−3.24 ± 0.03, −6.40 ± 0.18)_Mercury_ in Fig. 1a (see also Supplementary Table 9 and Supplementary Fig. 22).

This scenario predicts that the isotopic compositions of Venus and Mercury lie to more extreme values than Earth. One possibility is that incomplete mixing took place in the nebular gas, before condensation of grains was able to modify volatile/refractory element ratios. Dynamically, this could relate to turbulent mixing and/or diffusive transport in the early stages of disk accretion due to infall^47^, where temperatures at the midplane in the terrestrial planet-forming region still exceeded the condensation temperatures of oxides and silicates^48^. The Gaussian mass distribution could be achieved either by a Grand Tack-like scenario^49^ or through pressure bumps in the disk that led to the formation of a ring^50,51^. If the terrestrial planets accreted from individual ‘rings’ at different heliocentric distances, then the predicted compositions of Venus and Mercury need not adhere to the single Gaussian shown in Fig. 3 but could represent individual Gaussians that can move in Euclidean space. In either case, because Venus and Earth together represent the bulk of the mass in the inner Solar System, they should bear a strong isotopic resemblance to one another. The corollary is that the peripheral bodies (Mercury, Mars and Vesta) should have more extreme compositions, as they stochastically sample poorly mixed tails (Fig. 3). Sample return missions to the inner planets, Venus and Mercury, are sorely needed to test these ideas.

## Methods

### Data aggregation and curation

Isotopic data were sourced from the compilation maintained by OriginsLab, hereinafter, OLC^9^, the sole exception being Mo isotopic data for an estimate of the BSE, which were supplemented by two, more recent, papers^20,32^ (Supplementary Section 1.1.1). The error-weighted mean, *μ*_*i*_ and its standard error *σ*_*i*_ for each reservoir were calculated from the raw, published data by defining a weighting factor, *ω*_*i*_ for each individual sample, *a*, of a given isotope ratio, *i* (after ref. ^41^):

4 $${\omega }_{i}^{a}=\frac{1}{{({\sigma }_{i}^{a})}^{2}}.$$

The sum across all samples of the products of the weighting factors (${\omega }_{i}^{a}$) and the measured values (${x}_{i}^{a}$), divided by the sum of the weighting factors, for each individual sample, yields the group mean value,

5 $${\mu }_{i}=\frac{{\sum }_{a}{\omega }_{i}^{a}{x}_{i}^{a}}{{\sum }_{a}{\omega }_{i}^{a}}$$

and the associated standard error

6 $${\sigma }_{i}=\sqrt{\frac{1}{{\sum }_{a}{\omega }_{i}^{a}}}.$$

To detect and remove outliers from the group mean, Grubbs’ test was implemented, in which the weighted standard deviation was calculated using

7 $${\sigma }_{w}=\sqrt{\frac{{\sum }_{a}{\omega }_{i}^{a}{({x}_{i}^{a}-{\mu }_{i})}^{2}}{{\sum }_{a}{\omega }_{i}^{a}}},$$

and a Grubbs test statistic was calculated for each sample ${x}_{i}^{a}$ using

8 $$G=\frac{| {x}_{i}^{a}-{\mu }_{i}| }{{\sigma }_{w}}$$

and was compared with the critical value, here defined for *α* = 0.05,

9 $${G}_{\mathrm{crit}}=\frac{(n-1){t}_{\alpha /2n,n-2}}{\sqrt{n\left(n-2+{t}_{\alpha /2n,n-2}^{2}\right)}}$$

where *t*_*α*/2*n*,*n*−2_ is the critical value from the *t*-distribution with *n* − 2 degrees of freedom. If *G* > *G*_crit_, then the data point was rejected and the test was iterated until there are no outliers remaining.

### PCA/B-LFA

A deterministic PCA was performed using the PCA class of the machine-learning library sklearn.decomposition version 0.24.2^52^ on the means of the isotopic compositions of the reservoirs (Supplementary Table 1) to obtain the principal components (PCs) that maximize the combined variance along orthogonal axes (Supplementary Table 2). PCA provides a deterministic solution for dimensionality reduction but does not incorporate measurement uncertainties. To address this limitation, we implemented a B-LFA^53,54^ using PyMC5, which explicitly models uncertainties in the isotopic composition of each reservoir. In this framework, the PCs, which lack associated uncertainty, serve as initial estimates (priors) for the LFs (scores) and the loading matrix. Unlike PCA, which assumes a fixed solution, B-LFA allows probabilistic inference by treating the LFs as a multivariate normal distribution, where the mean is derived from the deterministic PCA solution. To ensure flexibility, each LF was assumed to have unit variance (that is, independent and standardized). In addition, a non-informative gamma distribution was used as a hyperprior on the precision (inverse variance) of the loading matrix, enabling the model to adjust uncertainty independently for each LF.

To further improve robustness, we modelled the observed data with a Student’s *t*-distribution, which mitigates sensitivity to outliers. The mean of this distribution was defined as the matrix product of the loading matrix and the LFs, and the noise scales were set using the standard deviations derived from the isotope data (Supplementary Table 1). To accommodate both nearly normal and heavy-tailed data, the normality parameter of the Student’s *t*-distribution was assigned an exponential prior (see, for example, ref. ^55^). For inference, we performed 12,000 steps to sample the posterior distribution. In summary, by leveraging a Bayesian approach, our method extends PCA by incorporating uncertainty, which enables a more accurate and probabilistic characterization of isotopic compositions across different reservoirs.

### York multivariate linear regression

We employed the York approach^56^ to compute linear regressions among isotopic ratios in reservoirs with means *x* and *y* and standard errors *σ*_*x*_ and *σ*_*y*_ following the widely used method of ref. ^41^ (their equation 13):

10a $$a=\bar{{\rm{y}}}-b\bar{x}$$

10b $$b=\frac{\sum W\beta V}{\sum W\beta U}$$

10c $${\sigma }_{a}^{2}=\frac{1}{\sum W}+{\bar{x}}^{\,2}{\sigma }_{b}^{2}$$

10d $${\sigma }_{b}^{2}=\frac{1}{\sum W{u}^{2}}.$$

where *a* and *b* are the intercept and slope of the line, *σ*_*a*_ and *σ*_*b*_ are their associated standard errors and *u* = *x* − *x*_adj_ where *x*_adj_ is the adjusted value of *x* according to the fit. The term *β* given by

11 $$\beta =W\left(\frac{U}{\omega (y)}+\frac{bV}{\omega (x)}-(bU+V\,)\frac{r}{\alpha }\right),$$

adjusts the contributions of individual data points based on the relative uncertainties in *x* and *y* (*ω*, equation (4)), where the regression weighting factor, *W* is

12 $$W=\frac{\omega (x)\omega (y)}{\omega (x)+{b}^{2}\omega (y)-2br\alpha },$$

and $U=x-\overline{x}$, $V=y-\overline{{\rm{y}}}$ (where $\overline{x}$ and $\overline{{\rm{y}}}$ denote the predicted values), *r* is the correlation coefficient between errors in *x* and *y*, and *α* is the geometric mean in the standard errors of *x* and *y*. Here we set *r* = 0; that is, we impose the condition that errors in the means of two isotopic ratios, *x* and *y*, are uncorrelated. This simplifies equations (11) and (12), such that the best-fit values of *a* and *b* are symmetrical.

The goodness-of-fit is quantified by the metric ‘GOF’, defined as

13 $$\mathrm{GOF}=\frac{{\sum }_{i}{W}_{i}{(\,{y}_{i}-a-b{x}_{i})}^{2}}{n-2}=\frac{{\chi }^{2}}{\nu },$$

where the term in brackets yields the residuals (the difference between the observed and predicted values of *x* and *y*) and *n* is the number of data points used in the regression.

#### Prediction of the composition of the BSE

Here we postulate that the composition of the BSE is consistent with a linear extension of the array defined by reservoirs in the OC–EC subgroup of NC meteorites in *ε–ε* (*x*–*y*) space. To test this hypothesis, we fixed the isotopic composition of the BSE in one of the ten isotopic systems considered here, termed the predictor, *x*_pred_, and computed the remaining nine isotopic ratios (*n* = 9) according to

14 $${\bf{y}}={\bf{b}}\cdot ({x}_{\mathrm{pred}}\pm {\sigma }_{{x}_{\mathrm{pred}}})+{\bf{a}},$$

where **y**, **b** and **a** are the column vectors containing the resulting *y* values and the input *b* (slope) and *a* (intercept) values as well as their associated standard errors (*σ*):

15 $${\bf{y}}=\left[\begin{array}{l}{y}_{1}\pm {\sigma }_{{y}_{1}}\\ {y}_{2}\pm {\sigma }_{{y}_{2}}\\ \vdots \\ {y}_{n}\pm {\sigma }_{{y}_{n}}\end{array}\right],\,{\bf{b}}=\left[\begin{array}{l}{b}_{1}\pm {\sigma }_{{b}_{1}}\\ {b}_{2}\pm {\sigma }_{{b}_{2}}\\ \vdots \\ {b}_{n}\pm {\sigma }_{{b}_{n}}\end{array}\right],\,{\bf{a}}=\left[\begin{array}{l}{a}_{1}\pm {\sigma }_{{a}_{1}}\\ {a}_{2}\pm {\sigma }_{{a}_{2}}\\ \vdots \\ {a}_{n}\pm {\sigma }_{{a}_{n}}\end{array}\right].$$

To calculate the uncertainties associated with the unknown **y**, a Monte Carlo simulation was performed in which the values of *x*_pred_, **b** and **a** were all varied over 10^4^ iterations according to an assumed normally distributed *σ* about their mean values.

To leverage all regressions to compute the value of the BSE, the weighted mean of the predicted *y* value of isotope ratio *j*, ${\widehat{y}}_{j}$, was calculated based on the *y*_*j*_ values given by the other nine predictor isotope ratios (*x*_*i*,pred_, *i* ≠ *j*), following equations (4) to (6).

### Permissible fraction of CI material in the BSE

This exercise was designed to determine the maximum allowed fraction of CC material that can be added to the BSE, assuming that it represents an NC body. To do so, the mass balance equation was solved in two ways. The first used concentration-weighted mass fractions of the present-day BSE and CI chondrites^57^ to compute the isotopic composition of the mixture:

16 $${\varepsilon }_{{\rm{BSE}}+{\rm{CI}},i}={w}_{{\rm{BSE}},i} ({\varepsilon }_{{\rm{BSE}},i}\pm {\sigma }_{{\rm{BSE}},i})+{w}_{{\rm{CI}},i} ({\varepsilon }_{{\rm{CI}},i}\pm {\sigma }_{{\rm{CI}},i})$$

where *ε*_BSE,*i*_ and *ε*_CI,*i*_ are the isotopic composition of element *i* in the BSE or CI chondrites, respectively, and

17 $${w}_{{\rm{B}}{\rm{S}}{\rm{E}},i}=\left(\frac{{c}_{\mathrm{BSE},i} {f}_{\mathrm{BSE},i}}{{c}_{\mathrm{BSE},i} {f}_{\mathrm{BSE},i}+{c}_{\mathrm{CI},i} {f}_{\mathrm{CI},i}}\right),{w}_{{\rm{C}}{\rm{I}},i}=\left(\frac{{c}_{\mathrm{CI},i} {f}_{\mathrm{CI},i}}{{c}_{\mathrm{BSE},i} {f}_{\mathrm{BSE},i}+{c}_{\mathrm{CI},i} {f}_{\mathrm{CI},i}}\right)$$

are the concentration-weighted mass fractions of the BSE and CI chondrites, respectively, in the mixture. Here *c* denotes the concentration of *i* and *f*_BSE,*i*_ = 1 − *f*_CI,*i*_ denotes the relative mass fraction of BSE in the mixture contributing to *i*. The second method was designed such that the concentration of element, *i*, was identical in the BSE-like and in CI-like endmembers: that is, *c*_BSE,*i*_ = *c*_CI,*i*_ for all *i*, for which equation (16) simplifies to

18 $${\varepsilon }_{{\rm{B}}{\rm{S}}{\rm{E}}+{\rm{C}}{\rm{I}},i}={f}_{{\rm{B}}{\rm{S}}{\rm{E}},i} ({\varepsilon }_{{\rm{B}}{\rm{S}}{\rm{E}},i}\pm {\sigma }_{{\rm{B}}{\rm{S}}{\rm{E}},i})+{f}_{{\rm{C}}{\rm{I}},i} ({\varepsilon }_{{\rm{C}}{\rm{I}},i}\pm {\sigma }_{{\rm{C}}{\rm{I}},i}).$$

The value of *f*_CI_ is sampled from 0 to 1 in 10,000 steps and, at each *f*_CI_, we performed *N* = 10,000 Monte Carlo simulations, where the isotopic compositions *ε*_BSE_ and *ε*_CI_ for the ten isotopic ratios (that is, *ε*^48^Ca, *ε*^50^Ti, *ε*^54^Cr, *ε*^54^Fe, *ε*^64^Ni, *ε*^66^Zn, *ε*^96^Zr, *ε*^94^Mo, *ε*^95^Mo and *ε*^100^Ru) were randomly drawn from normal distributions centred on their measured values with standard deviations corresponding to their measurement standard errors (*σ*_BSE_ and *σ*_CI_).

To determine the acceptable CI mass fraction for each *N*, the computed mixture composition for each element *i* must satisfy

19 $${\varepsilon }_{{\rm{BSE}},i}-{\sigma }_{{\rm{BSE}},i}\le {\varepsilon }_{{\rm{BSE}}+{\rm{CI}},i}\le {\varepsilon }_{{\rm{BSE}},i}+{\sigma }_{{\rm{BSE}},i}$$

where *ε*_BSE+CI,*i*_ is the simulated mixture composition for element *i*. A simulation was considered successful only if this condition held for all elements simultaneously. The fraction of successful models as a function of *f*_CI_ yields an upper bound on the permissible CI fraction in the BSE.

### Prediction of the isotopic compositions of Venus and Mercury

Leveraging the observations that: (1) the masses of the terrestrial planets are distributed in an approximately Gaussian manner about 0.9 AU as a function of semimajor axis; and (2) the semimajor axis is correlated with the isotopic Euclidean distance (*R*_A_) of a body, A (A = Earth (BSE), Mars and Vesta) computed according to equations (2) and (3), we predicted the isotopic Euclidean distances of Venus (*R*_Venus_) and Mercury (*R*_Mercury_). To do so, we fitted a mass-conserving Gaussian to the masses of Earth and Mars given their determined values of ${R}_{{\rm{A}}}^{{\rm{s}}}$ across all isotopic systems (Supplementary Table 4);

20 $$M({R}_{{\rm{A}}})=\sum {M}_{\mathrm{bodies}} \frac{2}{\sigma \sqrt{2{\rm{\pi }}}}\exp \left(-\frac{{({R}_{{\rm{A}}}-\mu )}^{2}}{2{\sigma }^{2}}\right)$$

where ∑*M*_bodies_ is the combined masses of Mercury, Venus, Earth and Mars, *μ* is the mean of the Gaussian distribution and *σ* is its standard deviation. This procedure was repeated 10^4^ times by Monte Carlo sampling over normal distributions about the mean of the values of *R*_A_ of the Earth and Mars to assess their influence on best-fit values of *μ* and *σ*. The values of *R*_Venus_ and *R*_Mercury_ were recovered by inserting their known values of *M* into equation (20) and solving for *R*_A_.

## Supplementary information

Supplementary Information Supplementary Text, Figs. 1–22 and Tables 1–9.

Peer Review File

Supplementary Data Tables Supplementary Data Table 1 with tabs: NC_CC_AllData_R1, regression_results, all_idata_summary, heavy_idata_summary, iron-peak_idata_summary, siderophile_idata_summary, lithophile_idata_summary.

## Data Availability

All data generated in this work are available in the main text and Supplementary Information.

## Code availability

The combined PCA–B-LFA was performed using the open-source Python package Bedroc v.0.2.0, which is available at https://github.com/ExPlanetology/bedroc. The multivariate linear regression analysis and Gaussian fitting model are available as Python scripts at the associated OSF repository^58^.
