# Supplementary material for: Homogeneous accretion of the Earth in the inner Solar System
Source: Nat Astron. 2026 Mar 27;10(7):972–9. doi: 10.1038/s41550-026-02824-7 (PMC13379315; doi:10.1038/s41550-026-02824-7)
Supplement: Supplementary file 1 — Supplementary Text, Figs. 1–22 and Tables 1–9. [file 41550_2026_2824_MOESM1_ESM.pdf]

nature astronomy

Supplementary information https://doi.org/10.1038/s41550-026-02824-7

Homogeneous accretion of the Earth in the inner Solar System

In the format provided by the authors and unedited

Supplementary Information

Contents

Supplementary Information 1

1 Data Curation 2
1.1 Full Dataset . . . 2
1.1.1 Molybdenum isotopic composition of the bulk silicate Earth . . 2

2 PCA / Bayesian Latent-Factor Analysis 3
2.1 Dataset for PCA / Bayesian Latent-Factor Analysis . . . 3
2.2 Supplementary Results for PCA / Bayesian-Latent Factor Analysis . . 7

3 Multivariate Linear Regression 10
3.1 Dataset for Multivariate Linear Regression . . . 10
3.2 Supplementary Results for Multivariate Linear Regression . . . 13
3.2.1 Correlation coefficient and Goodness-of-Fit . . . 13
3.2.2 Prediction of the isotopic composition of the bulk silicate Earth 15

4 Mass fraction of CI in the bulk silicate Earth 28

5 Prediction of the isotopic compositions of Venus and Mercury 30

6 Acknowledgments 36

7 Author contributions 36

8 Additional information 36

1 Data Curation

1.1 Full Dataset

All data for the groups (error-weighted mean and standard error) used as a basis for producing the models and figures in this work can be found in the Supplementary Data file, under the tab '*NC_CC_AllData*'. In the vast majority of cases, the group averages are identical to those reported in (1), which were calculated without outlier removal.

1.1.1 Molybdenum isotopic composition of the bulk silicate Earth

In determining the nucleosynthetic isotopic composition expressed in a given ratio, internal normalisation to a ratio of two, other isotopes is performed. For the isotopes of Mo, the normalisation is made by using the ^{98}Mo/^{96}Mo ratio, which is corrected to its assumed true value (1.453173, ref. 2) from the value measured by mass spectrometry according to the exponential law (3). However, as first shown by (2), correcting the measured isotopic composition in this manner results in 'U-shaped' patterns when the $\varepsilon^{i/96}$Mo anomaly is expressed relative to the $^{i/96}$Mo ratio of a standard, plotted as a function of $^{i/96}$Mo (their Fig. 1). The authors concluded that $\varepsilon^{i/96}$Mo ratios calculated in this manner reflect the inadequate correction of the mass-dependent isotopic fractionation endemic to the sample. The conclusion is that this leads to spurious $\varepsilon^{i/96}$Mo ratios, should the mass-dependent isotopic fractionation diverge markedly from that of the standard.

In order to circumvent such artifacts, here we re-calculate the ε^{94}Mo and ε^{95}Mo composition of the bulk silicate Earth (BSE) using data that report both the mass-independent (i.e., nucleosynthetic) and mass-dependent compositions on the same, terrestrial sample. To do so, we use the data of refs. (2) and (4). Mass-dependent isotopic compositions in the samples are expressed as δ^{98}Mo, where:

$$\delta^{98}Mo = \left[\frac{(^{98}Mo/^{95}Mo)_{smp}}{(^{98}Mo/^{95}Mo)_{std}} - 1\right] \times 1000. \quad \text{(S1)}$$

In Supplementary Figure 1, we find that ε^{94}Mo is strongly negatively correlated with δ^{98}Mo, whereas ε^{95}Mo is only weakly so given its closer proximity in atomic mass, to the normalising isotope ^{96}Mo. The linear fits were performed using the York regression scheme (see Methods, ref. 5). As such, the accuracy of the ε^{94}Mo composition of the BSE, in particular, is sensitive to the appropriate selection of samples that have undergone minimal mass-dependent isotopic fractionation relative to the standard.

As shown in Supplementary Figure 1, the analysed samples in refs. (2) and (4) define linear trends that overlap with one another, indicating no systematic offset between the two methods employed by the authors. Furthermore, such linearity is an indication that mass-dependent fractionation effects on the ε^{94}Mo ratio are not cumulative; that is, even if the δ^{98}Mo of a sample with a value close to that of the normalising standard was acquired through multiple mass fractionation events, this does not result in any substantial spurious ε^{94}Mo fractionation.

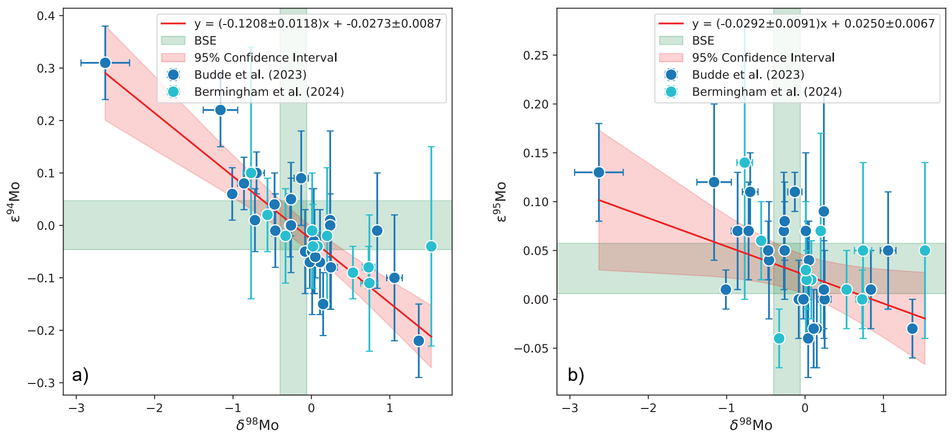

Supplementary Figure 1 Variations of the mass-dependent isotopic compositions of terrestrial samples, expressed as δ^{98}Mo, relative to those in nucleosynthetic isotopic anomalies corrected according to the exponential law and internally normalised to the ^{98}Mo/^{96}Mo ratio for a) ε^{94}Mo and b) ε^{95}Mo. All data are mean values of replicate analyses and their 2-σ standard errors from (2) (blue points) and (4) (teal points). The red line represents the best fit to the data using the York regression (5) and the red field the 95 % confidence interval on the regression. The recovered of the bulk silicate Earth (BSE) for ε^{94}Mo and ε^{95}Mo for a range of δ^{98}Mo = -0.23±0.17 (2-σ), is given by the green, horizontal bar.

The key consideration in order to assess whether the BSE expresses nucleosynthetic Mo isotopic deviations from meteorites, is to ensure that the *relative* magnitude of mass-dependent isotopic correction remains constant among them. That is, the samples chosen to represent the BSE value should have δ^{98}Mo isotopic values similar to those of meteorites to which it is compared. The range of δ^{98}Mo values among chondrites is close to -0.2 ‰ (e.g., 6), which is indistinguishable from that of the bulk silicate Earth (7; 8). Here, we take an average value of δ^{98}Mo = -0.23 ± 0.17 ‰, a conservative 2σ standard error to represent the range of plausible terrestrial and extraterrestrial compositions. This value is used in concert with the range of nucleosynthetic isotopic anomalies in Supplementary Figure 1 to derive values of ε^{94}Mo = 0.00 ± 0.05 and ε^{95}Mo = 0.03 ± 0.03, which are adopted throughout this study. These values lie between the previously reported estimates of 0.04 ± 0.06 and 0.10 ± 0.04 (9) and of -0.07 ± 0.03 and -0.01 ± 0.03 (4).

2 PCA / Bayesian Latent-Factor Analysis

2.1 Dataset for PCA / Bayesian Latent-Factor Analysis

The Principal Component Analysis (PCA) dimensionality reduction scheme is based on the principle that the principal components are computed by eigenvalue decomposition of the covariance matrix. The covariance matrix describes the correlation between any two values (here, isotope ratios) i and j, in a reservoir, R ($r \in 1, ..., R$), and is calculated as:

$$C_{ij} = \frac{1}{R}\sum_{r=1}^{R}(X_{r,i} - \hat{X}_i)(X_{r,j} - \hat{X}_j) \quad \text{(S2)}$$

where $X_{r,i}$ is the values of isotope ratio i in the rth reservoir, and $\hat{X}_i$ is the mean of the ith isotope ratio across all reservoirs. This leads to the condition that $X_{r,i}$ must exist across all i, otherwise the covariance matrix is undefined. To ensure sufficient data coverage, we apply a threshold, including only reservoirs with data for at least 8 out of the 10 isotope ratios, i (see the tab '*NC_CC_AllData*' in the Supplementary Data, Section 1.1) are selected for PCA / B-LFA. These are shown in Supplementary Table 1.

Reservoir	Group	ε^{48}Ca	ε^{50}Ti	ε^{54}Cr	ε^{54}Fe	ε^{64}Ni	ε^{66}Zn	ε^{96}Zr	ε^{94}Mo	ε^{95}Mo	ε^{100}Ru
CI	CC	2.07 ± 0.07	1.91 ± 0.06	1.47 ± 0.09	0.031 ± 0.016	0.62 ± 0.10	0.30 ± 0.07	0.81 ± 0.16	0.79 ± 0.41	0.69 ± 0.25	-0.24 ± 0.13
CM	CC	3.13 ± 0.14	2.95 ± 0.10	0.98 ± 0.06	0.228 ± 0.027	0.37 ± 0.09	0.25 ± 0.05	1.23 ± 0.07	2.33 ± 0.90	1.42 ± 0.22	-0.69 ± 0.24
CO	CC	3.87 ± 0.56	3.41 ± 0.35	0.84 ± 0.13	0.163 ± 0.045	0.26 ± 0.11	0.24 ± 0.02	1.09 ± 0.07	1.31 ± 0.25	0.75 ± 0.17	-0.92 ± 0.45
CV	CC	2.51 ± 0.02	3.35 ± 0.14	0.91 ± 0.03	0.269 ± 0.036	0.30 ± 0.03	0.29 ± 0.04	1.16 ± 0.16	1.11 ± 0.04	1.01 ± 0.03	-1.06 ± 0.08
CR	CC	2.10 ± 0.02	2.22 ± 0.24	1.29 ± 0.06	0.323 ± 0.046	0.26 ± 0.15	0.30 ± 0.03	1.18 ± 0.09	2.22 ± 0.27	1.32 ± 0.19	-0.95 ± 0.13
H	NC	-0.24 ± 0.29	-0.57 ± 0.09	-0.38 ± 0.02	0.116 ± 0.047	-0.16 ± 0.05	-0.30 ± 0.05	0.34 ± 0.02	0.67 ± 0.07	0.29 ± 0.04	-0.30 ± 0.05
L	NC	-0.21 ± 0.05	-0.63 ± 0.02	-0.32 ± 0.06	0.106 ± 0.05	-0.11 ± 0.06	-0.25 ± 0.03	0.30 ± 0.03	0.59 ± 0.19	0.21 ± 0.11	-0.28 ± 0.13
LL	NC	-0.36 ± 0.02	-0.65 ± 0.05	-0.42 ± 0.05	0.129 ± 0.04	-0.18 ± 0.05	-0.27 ± 0.03	0.29 ± 0.02	0.62 ± 0.11	0.20 ± 0.06	*-0.29 ± 0.09**
EH	NC	-0.11 ± 0.02	-0.12 ± 0.07	0.00 ± 0.07	*0.064 ± 0.03**	0.07 ± 0.03	-0.16 ± 0.03	0.15 ± 0.05	0.45 ± 0.08	0.21 ± 0.06	-0.08 ± 0.08
EL	NC	-0.18 ± 0.04	-0.31 ± 0.06	0.03 ± 0.07	0.064 ± 0.03	-0.05 ± 0.04	-0.16 ± 0.04	0.17 ± 0.03	0.27 ± 0.04	0.12 ± 0.04	-0.08 ± 0.05
Ureilites	NC	-1.33 ± 0.05	-1.97 ± 0.15	-0.88 ± 0.04	0.149 ± 0.012	-0.20 ± 0.21	-0.34 ± 0.02	*0.50 ± 0.10**	0.93 ± 0.04	0.42 ± 0.03	-0.30 ± 0.05
Mars	NC	-0.20 ± 0.02	-0.44 ± 0.04	-0.18 ± 0.03	0.061 ± 0.02	0.08 ± 0.11	-0.20 ± 0.03	0.27 ± 0.02	0.33 ± 0.06	0.25 ± 0.04	*-0.17 ± 0.10**
Vesta Group†	NC	-0.99 ± 0.04	-1.30 ± 0.05	-0.69 ± 0.02	0.120 ± 0.025	*-0.19 ± 0.06**	-0.21 ± 0.09	0.38 ± 0.03	1.03 ± 0.03	0.47 ± 0.02	-0.42 ± 0.02
Earth	BSE	0.01 ± 0.04	0.01 ± 0.05	0.10 ± 0.12	-0.008 ± 0.019	0.12 ± 0.02	-0.07 ± 0.03	0.02 ± 0.02	0.00 ± 0.05	0.03 ± 0.03	0.02 ± 0.02

†Vesta Group is calculated as a weighted average of the following reservoirs: Vesta, Brachinites, Main Group Pallasites, Mesosiderites, Lodranites, Acapulcoites and Angrites. See text for more information.

*Indicates values that were estimated (not measured) in order to perform the PCA. See text for more information.

NB: Some data for Ni isotopes are originally reported as ε^{62}Ni. We converted all data to ε^{64}Ni according to the relation: $\varepsilon^{64}\text{Ni} = (2.84 \pm 0.31)\varepsilon^{62}\text{Ni}$ (1).

Key References.

Ca. (10; 11; 12). **Ti.** (13; 14; 15; 16; 17). **Cr.** (18; 19; 20; 21; 22; 23). **Fe.** (24; 25). **Ni.** (26; 27; 28; 29; 30). **Zn.** (31; 32; 33; 34; 35). **Zr.** (36; 37; 38). **Mo.** (39; 40; 9; 41; 16; 2; 42; 4). **Ru.** (43; 44; 45; 40; 46; 47).

Supplementary Table 1 Data for all reservoirs used in the PCA / B-LFA analysis reported as mean and 2σ standard errors.

Of the 14 reservoirs listed in Supplementary Table 1, only the Vesta Group represents a composite reservoir, constructed from a weighted average of data for Vesta, Brachinites, Main Group Pallasites, Mesosiderites, Lodranites, Acapulcoites and Angrites. They are not taken to represent a single parent body, but as a group of small telluric bodies whose nucleosynthetic isotope ratios are similar to one another (see 17).

One rationale for doing so lies in the overlapping (Brachinites), indistinguishable (Mesosiderites) and similar (Main Group Pallasites) Δ^{17}O isotopic compositions of these three groups with those of the Howardite-Eucrite-Diogenite clan of meteorites from 4-Vesta (48). Indeed, Mesosiderites are thought to derive from the same parent body (i.e., 4-Vesta, 49), while Brachinites also share petrological features to HEDs in their high FeO contents (50). Main Group Pallasites are clearly distinguished from HEDs in Δ^{17}O, but their nucleosynthetic isotopic compositions for which data exist among the two meteorite groups (ε^{50}Ti, ε^{54}Cr and ε^{96}Zr) their compositions overlap within uncertainty. Angrites, acapulcoites and lodranites have distinct Δ^{17}O values to those from the aforementioned groups, yet, they too have ε^{50}Ti and ε^{54}Cr that overlap with those of other small telluric bodies (17).

To quantitatively formalise the criteria for the constituent members of this reservoir, we performed PCA / B-LFA on a subset of isotopic systems (ε^{48}Ca, ε^{50}Ti, ε^{54}Cr and ε^{96}Zr) among the H, L, LL, EH, EL, ureilites, Mars and Earth, together with individual members of bodies proposed to constitute the 'Vesta group'; Vesta, angrites, acapulcoites, mesosiderites and main group pallasites (Supplementary Figure 2).

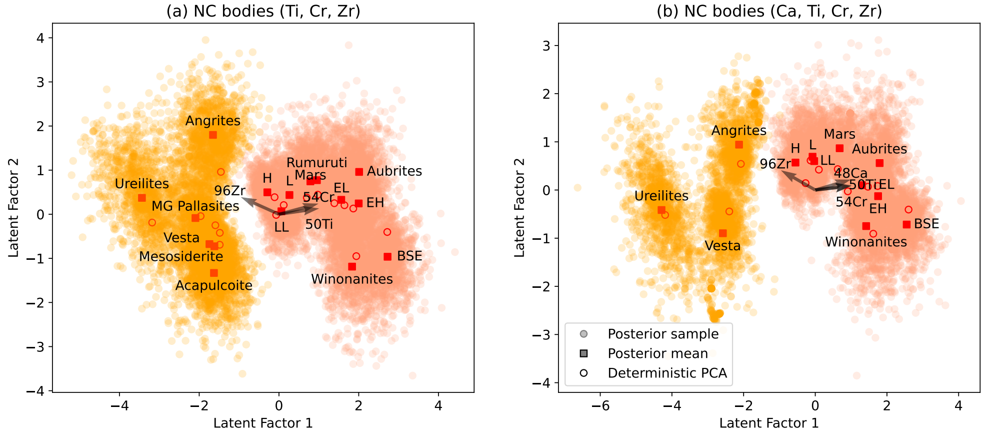

Supplementary Figure 2 Results of the Bayesian latent factor analysis (B-LFA) and deterministic Principal Component Analysis (PCA) for 10^4 iterations across a) ε^{50}Ti, ε^{54}Cr and ε^{96}Zr and b) ε^{48}Ca, ε^{50}Ti, ε^{54}Cr and ε^{96}Zr among individual NC bodies. See the tab '*NC_CC_AllData*' in the Supplementary Data. Refer to caption for Fig. 1 for more detail.

Supplementary Figure 2 shows that the Main Group Pallasites, Vesta, Mesosiderites and Acapulcoites (and hence Lodranites) have very similar LF1-LF2 coordinates. Angrites have indistinguishable LF1 values but higher LF2 values, which largely reflect their more *s*-process-depleted ε^{96}Zr. Yet, Angrites and other Vesta group meteorites have indistinguishable ε^{48}Ca and ε^{50}Ti anomalies. Therefore, on the main axis of variation, LF1, angrites should be grouped with Vesta group meteorites. We note that, since nucleosynthetic isotope compositions for the Angrites are available only for the four systems noted above, additional data is required to establish isotopic relationships for each of the 10 systems considered in this work.

For the reservoirs with less than 10 isotope ratios available, proxy values were estimated based on the following logic:

- **LL.** Missing value: ε^{100}Ru. Average of H and L chondrite values.
- **EH.** Missing value: ε^{54}Fe. Assumed identical to EL.
- **Ureilites.** Missing value: ε^{96}Zr. Estimated on the basis of linear correlations between ε^{96}Zr and other isotope ratios among the NC group.
- **Mars.** Missing value: ε^{100}Ru. Assumed intermediate between OC and EC bodies, as per other isotopic systems.
- **Vesta Group.** Missing value: ε^{64}Ni. Estimated on the basis of linear correlations between ε^{64}Ni with other iron-peak element isotopic ratios among NC bodies.

Finally, it should be noted that the PCA values are used only as *priors* for the B-LFA. The B-LFA yields the *posterior* probability function for each value, which differs from the prior value defined in Supplementary Table 1.

2.2 Supplementary Results for PCA / Bayesian-Latent Factor Analysis

Output data for the PCA / B-LFA can be found in the Supplementary Data file Excel workbook under the '*elementset_idata_summary*' tabs where '*elementset*' corresponds to the set of isotope ratios used for the analysis (e.g., iron-peak). A summary of the variance explained by each of the principal components (PCs) in the PCA is given in Supplementary Table 2.

Supplementary Table 2 Explained Variance (%) in Principal Component Analysis among different isotopic anomaly subsets.

	All	Siderophile	Iron-Peak	Heavy Element	Lithophile
**PC1**	73.59	71.20	78.68	86.00	90.54
**PC2**	16.53	18.85	16.02	9.76	6.06
**PC3**	5.03	7.13	3.46	3.31	2.52
**PC4**	2.32	2.52	1.21	0.93	0.63
**PC1+PC2**	90.12	90.05	94.70	95.76	96.60

An additional set of isotopic ratios, those corresponding to the lithophile elements, run in a manner identical to that used to produce Fig. 1 in the main text, is shown in Supplementary Figure 3, below.

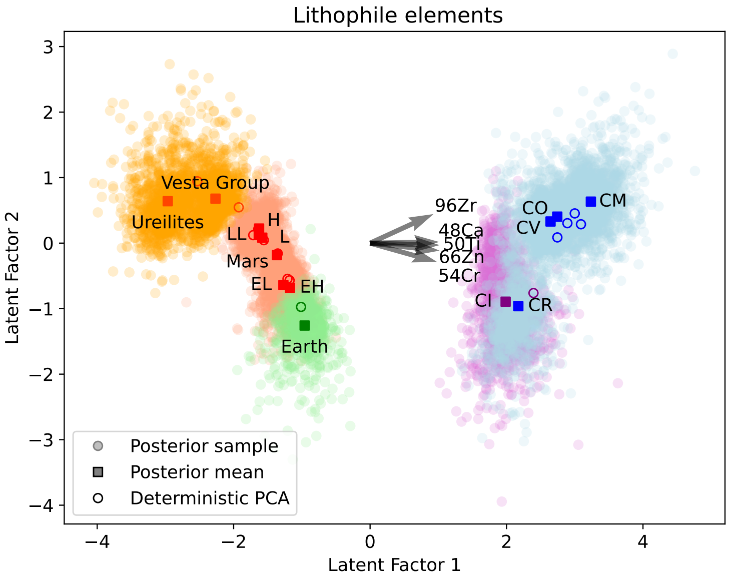

Supplementary Figure 3 Results of the Bayesian latent factor analysis (B-LFA) and deterministic Principal Component Analysis (PCA) for 10^4 iterations across the nucleosynthetic isotopic compositions among the lithophile elements. See Supplementary Table 1 for data. Refer to caption for Fig. 1 in the main text for more detail.

Evidence for the departure of the ureilites and the Vesta Group from the linear trend defined by the remaining NC bodies is sought by performing a linear regression, based on the York method (see *Methods*) to the coordinates of each of these bodies in LF1-LF2 space using the 'All elements' set of isotopic anomalies (Supplementary Figure 4, Supplementary Table 3).

Supplementary Table 3 Linear regression through groups consisting of different subsets of meteorites defining the 'EC-OC' subgroup of the NC trend using their LF1-LF2 values defined across all elements (Fig. 1(a) of the main text).

	**York Regression**					**% Change**	
Groups	a (slope)	σ_a	b (intercept)	σ_b	χ^2	a	b
H, L, LL, EH, EL	5.58	1.57	11.17	3.15	0.23	-5.1	-4.2
H, L, LL, Mars, EH, EL	5.88	1.59	11.66	3.18	0.33	0.0	0.0
H, L, LL, Mars, EH, EL, Earth	5.05	0.81	10.01	1.68	0.30	-14.1	-14.2
H, L, LL, Mars, EH, EL, Vesta Group	9.76	2.95	19.76	5.86	0.52	66.0	69.5

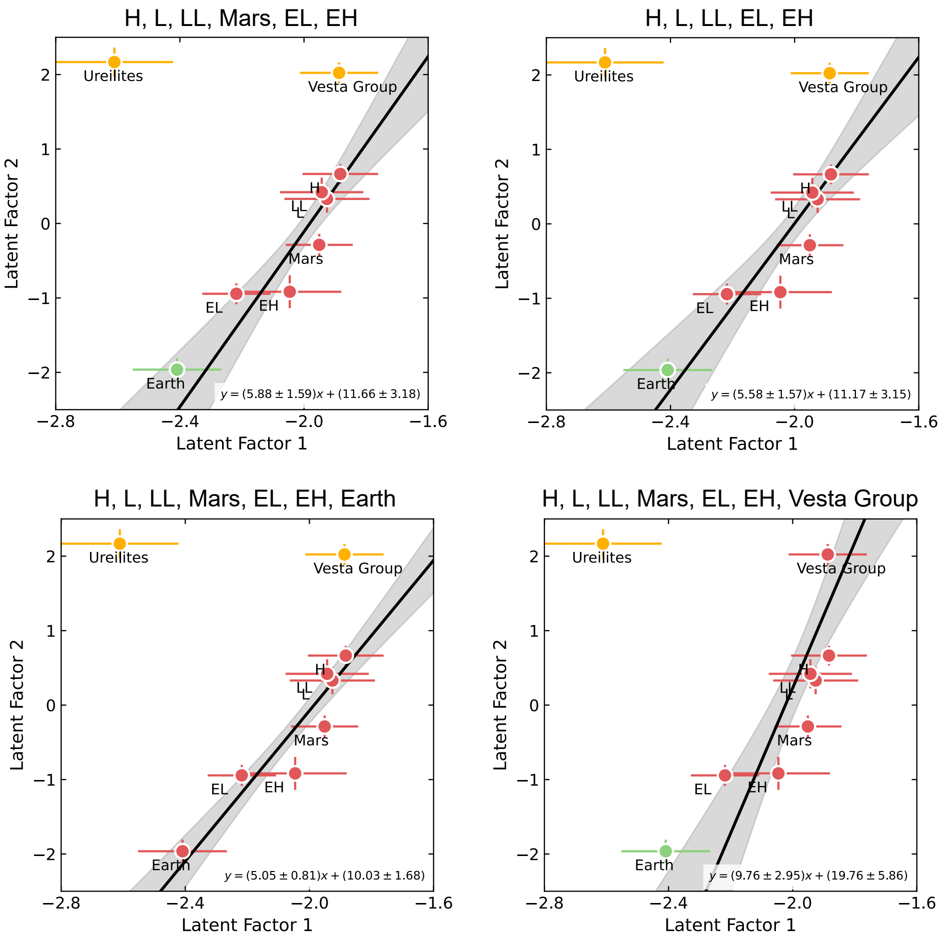

Supplementary Figure 4 Linear, York regressions (black line) with its 2-σ uncertainty envelope (grey field) through a subset of bodies that make up the NC trend based on their coordinates that describe their means and 2-σ uncertainties in Latent Factor 1 vs. Latent Factor 2 space, as taken from the Supplementary Data, under the tab '*all_idata_summary*' (Fig. 1a of the main text).

The analysis shows that, for combinations of just chondrites (H, L, LL, EH, EL) or chondrites with Mars (H, L, LL, Mars, EH, EL), there are no differences in slope and intercept within uncertainty (within $\sim$ 5 % relative) and the reduced χ^2 values are comparable (0.23 vs. 0.33; Supplementary Table 3), illustrating that Mars is a member of the OC-EC subgroup. A similar argument can be made for Earth. If, instead, the Vesta group is added (H, L, LL, Mars, EH, EL, Vesta Group), then both the slope and intercept, as well as the χ^2 increase by a factor $\sim$1.6, indicating a substantial decrease in goodness of fit. Indeed, the Vesta group lies marginally outside

the 2-σ uncertainty of the best fit line for all other combinations of NC bodies (Supplementary Figure 4). We therefore conclude the Vesta group, as currently defined, does not lie on a linear extension of the 'OC-EC' subgroup.

The Isotopic Euclidean Distance, computed as the Euclidean Distance between an NC body, A and the BSE (d_{A-BSE}), referenced to that between the mean of the ordinary chondrites and the BSE (d_{OC-BSE}) in LF1-LF2 space, is denoted R_A. The mean values of R_A and their uncertainties are given in Supplementary Table 4 for different subsets of isotopic anomalies.

Supplementary Table 4 Isotopic Euclidean Distances (R_A) among different isotopic anomaly subsets.

R_A	All	Siderophile	Iron-Peak	Heavy Element	Lithophile
**BSE**	0	0	0	0	0
**EC**	0.41±0.10	0.46±0.15	0.34±0.24	0.52±0.38	0.43±0.38
**Mars**	0.69±0.07	0.48±0.20	0.57±0.15	0.70±0.33	0.74±0.24
**OC**	1	1	1	1	1
**Vesta Group**	1.55±0.07	1.32±0.12	1.29±0.12	1.66±0.33	1.49±0.27

3 Multivariate Linear Regression

3.1 Dataset for Multivariate Linear Regression

The object of this exercise is to determine whether the bulk silicate Earth lies on an extension of the linear trend defined by the EH, EL, Mars, LL, L and H meteorites ('OC-EC' subgroup of the NC group) in Fig. 1 in the main text. Note that, although the LL, L and H chondrites could be grouped into a single point representing ordinary chondrites, because the York regression applied is based on weighted averages, there is no statistical difference in grouping them or leaving them separate (as done here). It was shown in Fig. 1 in the main text and in Supplementary Section 2.2 (above), that the Vesta group and ureilites fall off the linear array defined by these bodies in LF1-LF2 space, and are hence not included in the Multivariate Linear Regression (MLR).

Other meteorite groups are not included in Fig. 1 in the main text due to lack of available data across a sufficient number of isotopic systems (compare the data in the Supplementary Data file tab '*NC_CC_AllData*' with those in Supplementary Table 1). In order to ascertain whether these bodies, which pertain largely to the iron meteorites and a selection of chondrite- (Kakangari, Rumuruti) and primitive achondrites (Aubrites, Winonaites), adhere to the trend defined by the OC-EC group in Fig. 1 in the main text, we perform the same exercise (PCA / B-LFA) and examine their distribution in LF1-LF2 space. To do so, a more limited number of isotopic anomalies are implemented, namely,

- Subset 1 - Supplementary Figure 5a. ε^{54}Fe, ε^{64}Ni, ε^{94}Mo, ε^{95}Mo, ε^{100}Ru.

- Subset 2 - Supplementary Figure 5b. ε^{54}Cr, ε^{94}Mo, ε^{95}Mo, ε^{100}Ru.

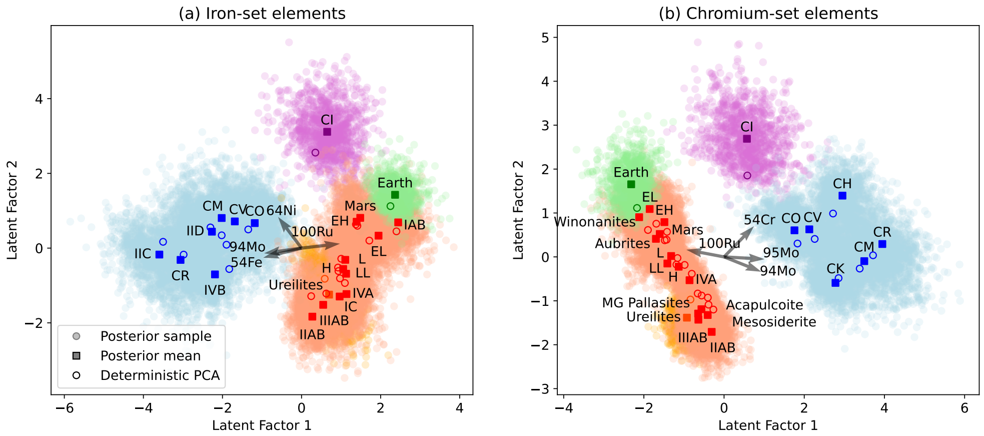

Supplementary Figure 5 Results of the Bayesian latent factor analysis (B-LFA) and deterministic Principal Component Analysis (PCA) across (a) Subset 1 and (b) Subset 2 with data taken for all available, individual reservoirs from the Supplementary Data file, under the tab '*NC_CC_AllData*'. The iron meteorites (IAB, IVA, IC, IIAB, IIIAB) and the primitive achondrites (Aubrites, Winonaites) have LF1-LF2 characteristics consistent with those of the group consisting of H, L, LL, Mars, EH and EL (the 'OC-EC' subgroup; red). As per other groups of isotopic anomalies, the bulk silicate Earth (green) is an extension of the 'OC-EC' subgroup for all element groups, regardless of their chemical or nucleosynthetic affinity. Open circles are computed from deterministic PCA. Transparent, coloured circles show samples from the posterior distribution and filled squares are the means of the B-LFA. The arrows denote the loading vector for each isotopic ratio to the latent factors (e.g., a reservoir with high ε^{100}Ru will plot to lower values of LF1 at near-constant LF2 in panel (b)). The Bayesian analysis captures the uncertainty associated with the latent variables and the standard error on the observed data.

The analysis shows that both the iron meteorite parent bodies and chondrite- and primitive achondrites are consistent with the linear trend defined by the OC-EC group. The Winonaites share affinities with the IAB iron meteorites in their Mo- and Ru isotopic compositions, consistent with their common origin inferred from O isotopes and petrologic considerations (51). Iron meteorites with more extreme nucleosynthetic isotope compositions (IIABs, IIIABs) also fall on an extension of the array defined by the 'OC-EC' subgroup. Consequently, these groups are included in the 'OC-EC' subgroup used to predict the composition of the BSE in the multivariate linear regressions, and the full dataset used in shown in Supplementary Table 5.

Reservoir	Group	Δ^{17}O	ε^{48}Ca	ε^{50}Ti	ε^{54}Cr	ε^{54}Fe	ε^{64}Ni	ε^{66}Zn	ε^{96}Zr	ε^{94}Mo	ε^{95}Mo	ε^{100}Ru
H	OC-EC	0.72 ± 0.05	-0.24 ± 0.29	-0.57 ± 0.09	-0.38 ± 0.02	0.116 ± 0.047	-0.16 ± 0.05	-0.30 ± 0.05	0.34 ± 0.02	0.67 ± 0.07	0.29 ± 0.04	-0.30 ± 0.05
L	OC-EC	1.03 ± 0.04	-0.21 ± 0.05	-0.63 ± 0.02	-0.32 ± 0.06	0.106 ± 0.05	-0.11 ± 0.06	-0.25 ± 0.03	0.30 ± 0.03	0.59 ± 0.19	0.21 ± 0.11	-0.28 ± 0.13
LL	OC-EC	1.19 ± 0.06	-0.36 ± 0.02	-0.65 ± 0.05	-0.42 ± 0.05	0.129 ± 0.04	-0.18 ± 0.05	-0.27 ± 0.03	0.29 ± 0.02	0.62 ± 0.11	0.20 ± 0.06	–
EH/Aub	OC-EC	0.00 ± 0.08	-0.11 ± 0.02	-0.10 ± 0.08	-0.02 ± 0.07	–	0.07 ± 0.03	-0.16 ± 0.03	0.19 ± 0.04	0.45 ± 0.08	0.22 ± 0.04	-0.06 ± 0.03
EL	OC-EC	-0.01 ± 0.07	-0.18 ± 0.04	-0.31 ± 0.06	0.03 ± 0.07	0.064 ± 0.03	-0.05 ± 0.04	-0.16 ± 0.04	0.17 ± 0.03	0.27 ± 0.04	0.12 ± 0.04	-0.08 ± 0.05
Kak	OC-EC	-1.09 ± 0.35	-1.30 ± 0.25	–	-0.44 ± 0.04	–	–	–	0.42 ± 0.04	–	–	–
Rum	OC-EC	2.64 ± 0.15	–	-0.47 ± 0.1	-0.06 ± 0.09	0.064 ± 0.03	–	–	0.25 ± 0.05	0.42 ± 0.09	0.18 ± 0.05	–
Win	OC-EC	-0.49 ± 0.07	-0.21 ± 0.09	-0.29 ± 0.11	-0.14 ± 0.04	–	–	–	0.02 ± 0.09	0.22 ± 0.06	0.09 ± 0.05	-0.06 ± 0.1
Mars	OC-EC	0.27 ± 0.03	-0.20 ± 0.02	-0.44 ± 0.04	-0.18 ± 0.03	0.061 ± 0.02	0.08 ± 0.11	-0.20 ± 0.03	0.27 ± 0.02	0.33 ± 0.06	0.25 ± 0.04	–
IAB*	Iron Meteorites	–	–	–	–	-0.01 ± 0.02	-0.06 ± 0.06	-0.19 ± 0.13	–	0.04 ± 0.05	-0.04 ± 0.03	-0.02 ± 0.05
IC	Iron Meteorites	–	–	–	–	0.10 ± 0.05	-0.24 ± 0.09	-0.33 ± 0.05	–	0.88 ± 0.03	0.34 ± 0.01	-0.28 ± 0.03
IIAB	Iron Meteorites	-0.66 ± 0.13	–	–	-0.83 ± 0.03	0.16 ± 0.03	-0.29 ± 0.1	–	–	1.18 ± 0.02	0.51 ± 0.02	-0.43 ± 0.02
IIE	Iron Meteorites	0.57 ± 0.05	–	–	-0.59 ± 0.13	–	–	–	–	0.68 ± 0.05	0.30 ± 0.03	–
IIIAB*	Iron Meteorites	-0.20 ± 0.04	–	–	-0.81 ± 0.05	0.10 ± 0.05	-0.32 ± 0.07	–	–	1.02 ± 0.05	0.44 ± 0.03	-0.50 ± 0.10
IIICD	Iron Meteorites	–	–	–	–	–	–	–	–	0.11 ± 0.03	-0.02 ± 0.04	–
IIIE	Iron Meteorites	–	–	–	–	–	-0.26 ± 0.08	–	–	0.95 ± 0.06	0.43 ± 0.04	-0.51 ± 0.06
IVA	Iron Meteorites	1.17 ± 0.10	–	–	-0.47 ± 0.06	0.10 ± 0.04	-0.28 ± 0.04	–	–	0.79 ± 0.04	0.40 ± 0.03	-0.28 ± 0.07

Supplementary Table 5 Data for all (NC) reservoirs used in the MLR analysis. Values are given with their 2σ uncertainties (±). Missing data is denoted as "–". NB. Δ^{17}O data were not used in the analysis, but are listed here for completeness. *We note the distinction between IAB and IIICD is outdated (52) but retain it here to conform with recent work on the topic (4).

3.2 Supplementary Results for Multivariate Linear Regression

3.2.1 Correlation coefficient and Goodness-of-Fit

The York regression procedure described in the *Methods* section is applied to all binary combinations of isotope ratios (110 in total) for the NC bodies given in Supplementary Table 5 to quantify $a \pm \sigma_a$ and $b \pm \sigma_b$ between each isotope pair. The results can be accessed in full in the Supplementary Data file, under the tab '*regression_results*'. The Pearson correlation coefficient matrix is shown in Supplementary Figure 6 and the Goodness-of-Fit (GOF) matrix in Supplementary Figure 7.

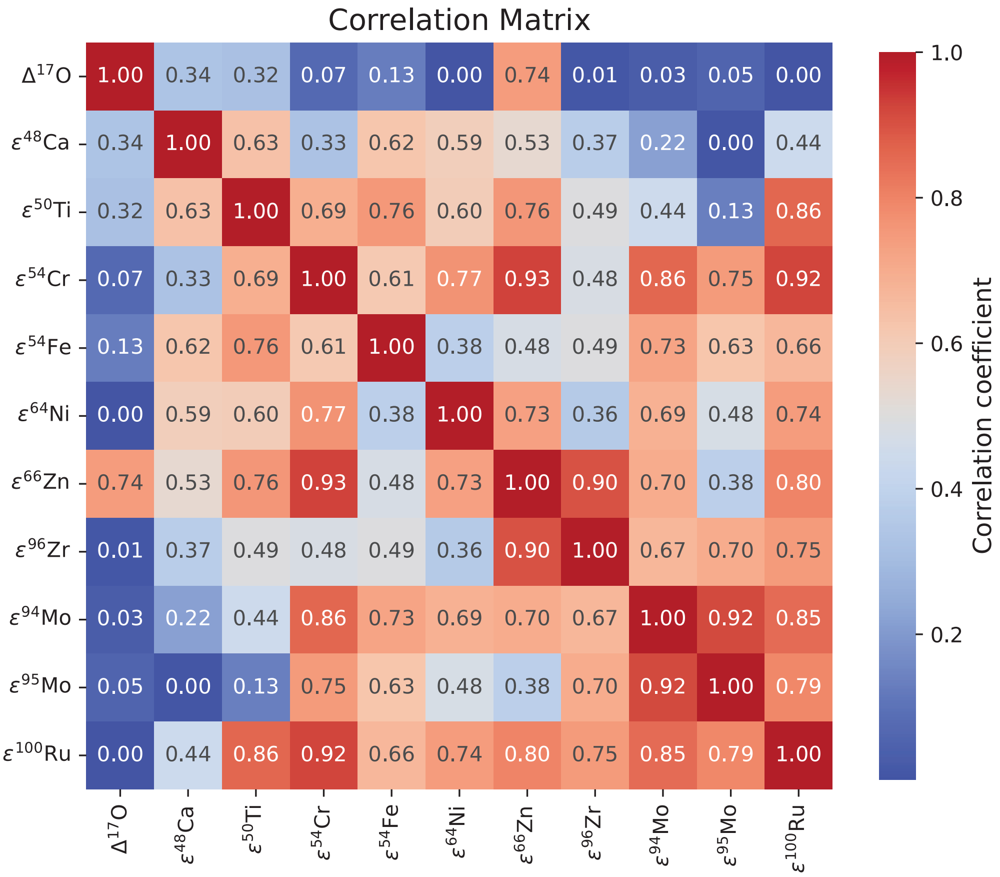

Supplementary Figure 6 Matrix showing the Pearson correlation coefficients (r^2) of binary isotope ratio pairs.

Immediately apparent is that the correlations between Δ^{17}O and the other isotopic systems in the NC meteorites is poor (<0.34, aside from 0.74 between ε^{66}Zn), a property reflected in the high (i.e., poor) Goodness-of-Fit (GOF). This observation substantiates the assertion that variations in Δ^{17}O are decoupled from those of the

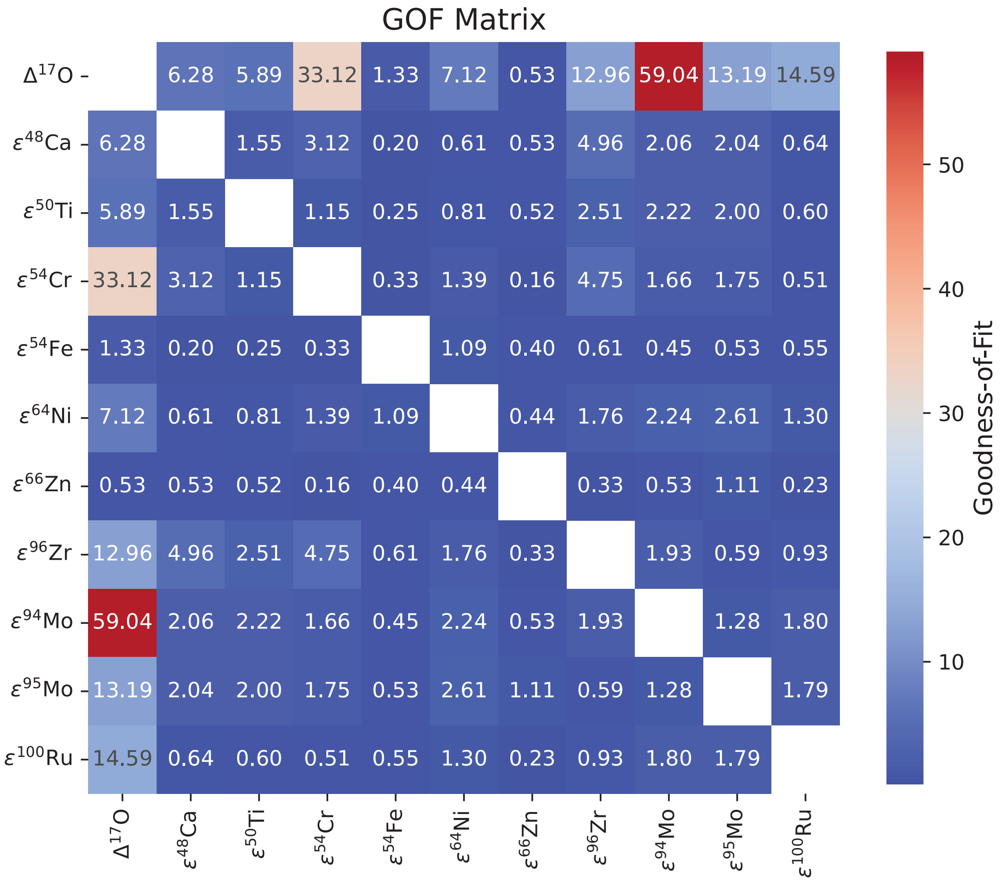

Supplementary Figure 7 Matrix showing the Goodness-of-Fit (eq. 13 of main text) of binary isotope ratio pairs.

other isotopic systems. Variations in, for example, ε^{i}Mo ratios among meteorites can be modelled to result from different proportions of isotopes produced by the nucleosynthetic $r-$ and $s-$processes (e.g., 9; 53) or in ^{54}Cr by supernovae (54; 55). That there is no correlation in the variations of isotopes of both iron-peak and heavy elements with those of Δ^{17}O implies that the variations in the latter are not solely nucleosynthetic in origin (56). Consequently, they are excluded from the MLR analysis.

The correlations in the remaining 10 isotopic systems are strong among NC group meteorites. The mean correlation coefficient (r^2) across all isotope ratio is 0.61±0.21 (1 sd), with ε^{48}Ca being the only system for which the mean r^2 is below 0.5 (0.42±0.21, 1 sd), owing to the lack of high-precision data for certain meteorite groups (Kakangari and H chondrites, see Supplementary Table 5). On the other hand, correlations between ε^{100}Ru and the other isotopic systems are uniformly high with

the exception of with ε^{48}Ca, leading to an average r^2 of 0.76±0.14 (1 sd).

Goodness-of-Fit values with the exception of ε^{48}Ca vs. ε^{96}Zr, and ε^{96}Zr vs. ε^{54}Cr, are lower than 3. Should the linear regression between two quantities be a reasonable description of the data, then GOF values should be close to unity. In some cases, notably for ε^{54}Fe and ε^{66}Zn, the GOF is often below 1 (down to 0.16 for ε^{66}Zn vs. ε^{54}Cr), implying the residuals of the York fit to the data are better than expected given the uncertainties. This arises because the overall spread in isotopic ratios is low relative to the uncertainties for these two isotopic systems.

This exercise serve to underline that, in the vast majority of cases, variations between two isotopic systems are satisfactorily described by a linear regression. Therefore, isotopic variations among the group of NC meteorites shown in Supplementary Table 5 are linearly correlated to a good approximation.

3.2.2 Prediction of the isotopic composition of the bulk silicate Earth

As described in the *Methods* section of the main text, the linear regression determined by York fit among the reservoirs in each binary ε-ε plot are used to compute the isotopic composition of the bulk silicate Earth (BSE), assuming *one* of the isotopic ratios is known (and is that of the BSE), termed the predictor, $x_{pred} \pm \sigma_{x_{pred}}$. To do so, a Monte Carlo simulation is run to sample the uncertainty in the predictor value, together with those in the slope and intercept of the regressions, 10,000 times assuming a Gaussian distribution about the mean values. This exercise yields a value for each of the remaining isotope systems, y^n_{BSE}, and the weighted mean across all predictor nine values are collated as $\hat{y}^n_{BSE}$. The weighted mean and associated standard error are shown in Supplementary Table 6 and in the main text in Fig. 2.

Supplementary Table 6 Predicted and observed isotopic composition of the bulk silicate Earth (BSE)

character	type	ratio	pred. mean ($\hat{y}_j$)	pred. st. err. ($\sigma_{\hat{y}_j}$)	obs. mean	obs. st. err.
lithophile	iron-peak	ε^{48}Ca	-0.03	0.02	0.01	0.04
lithophile	iron-peak	ε^{50}Ti	0.01	0.05	0.01	0.05
lithophile	iron-peak	ε^{54}Cr	0.17	0.03	0.10	0.12
siderophile	iron-peak	ε^{54}Fe	0.007	0.009	-0.008	0.019
siderophile	iron-peak	ε^{64}Ni	0.11	0.02	0.12	0.02
lithophile	iron-peak	ε^{66}Zn	-0.10	0.02	-0.07	0.03
lithophile	heavy	ε^{96}Zr	0.05	0.02	0.02	0.02
siderophile	heavy	ε^{94}Mo	0.05	0.03	0.00	0.05
siderophile	heavy	ε^{95}Mo	0.04	0.02	0.03	0.02
siderophile	heavy	ε^{100}Ru	0.03	0.02	0.02	0.02

In order to quantify the difference in the means of the distributions of the predicted values relative to the observed values of the BSE, the Z-score is computed for each predictor-predicted pair, according to:

$$Z = (y_{mod} - y_{obs})/\sqrt{\sigma^2_{mod} + \sigma^2_{obs}} \tag{S3}$$

where a Z-score < 1 indicates that the differences in the means of the two distributions are within one combined standard deviation of one another. The corresponding Z-score matrix is shown in Supplementary Table 7 and in Supplementary Figure 8.

Supplementary Table 7 Z-scores for binary isotope diagrams. Predictors are organised by columns and predicted values by rows.

	ε^{48}Ca	ε^{50}Ti	ε^{54}Cr	ε^{54}Fe	ε^{64}Ni	ε^{66}Zn	ε^{96}Zr	ε^{94}Mo	ε^{95}Mo	ε^{100}Ru
ε^{48}Ca		0.66	0.69	0.18	1.14	-0.31	-0.90	-0.46	2.24	0.94
ε^{50}Ti	-0.61		0.91	0.26	-0.17	-0.53	0.03	-0.10	-0.73	0.18
ε^{54}Cr	-0.64	-0.81		-1.25	-0.63	-0.65	0.32	-0.53	-0.85	-0.74
ε^{54}Fe	0.15	0.23	-1.78		0.85	0.11	-0.05	-0.51	-1.00	0.09
ε^{64}Ni	-0.93	0.14	0.63	0.86		-0.65	-1.06	0.15	0.50	0.86
ε^{66}Zn	0.50	0.92	1.08	0.17	1.29		-0.64	0.21	-0.09	0.66
ε^{96}Zr	-1.12	0.07	0.32	0.07	-1.85	-0.63		1.17	0.51	-0.97
ε^{94}Mo	-0.53	-0.13	-0.55	0.49	0.18	0.16	-1.35		-1.12	-0.32
ε^{95}Mo	-3.81	-1.35	-0.92	0.93	0.61	0.06	-0.41	1.12		0.27
ε^{100}Ru	-0.56	-0.17	0.80	0.10	-0.87	-0.40	-0.72	-0.31	0.26	

Converting the Z-scores into absolute differences, and comparing the averages for the different geochemical (i.e., lithophile or siderophile) and nucleosynthetic (i.e., iron-peak or heavy element) groups, no systematic difference in Z-scores is found (Supplementary Table 8). The mean across all Z-scores is 0.65±0.55.

Supplementary Table 8 Collated, absolute mean Z-scores between different isotopic groups according to their geochemical or nucleosynthetic affinity. Predictors are organised by columns and predicted values by rows.

	lithophile	siderophile
lithophile	0.73 ± 0.18	0.70 ± 0.26
siderophile	0.69 ± 0.38	0.57 ± 0.13
	iron-peak	heavy
iron-peak	0.68 ± 0.23	0.63 ± 0.23
heavy	0.75 ± 0.47	0.66 ± 0.21

Finally, visual representations of every binary ε-ε plot are shown, organised by predictor element, x_{pred}, in Supplementary Figures 9 to 18.

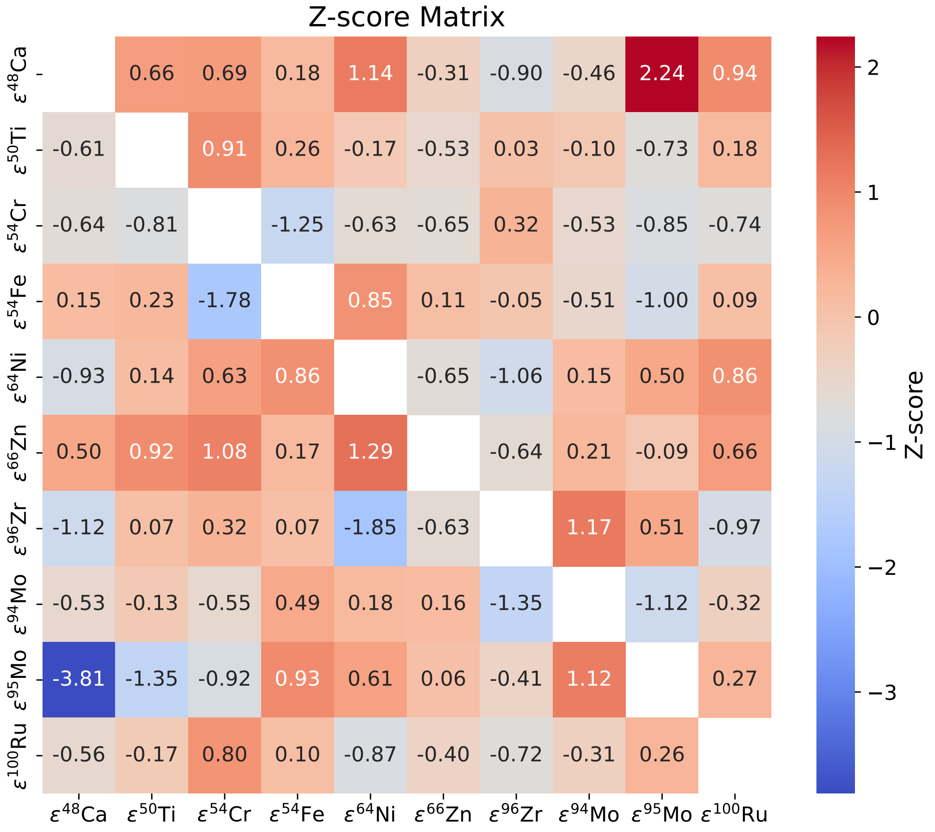

Supplementary Figure 8 Matrix showing the Z-score (eq. S3) of binary isotope ratios.

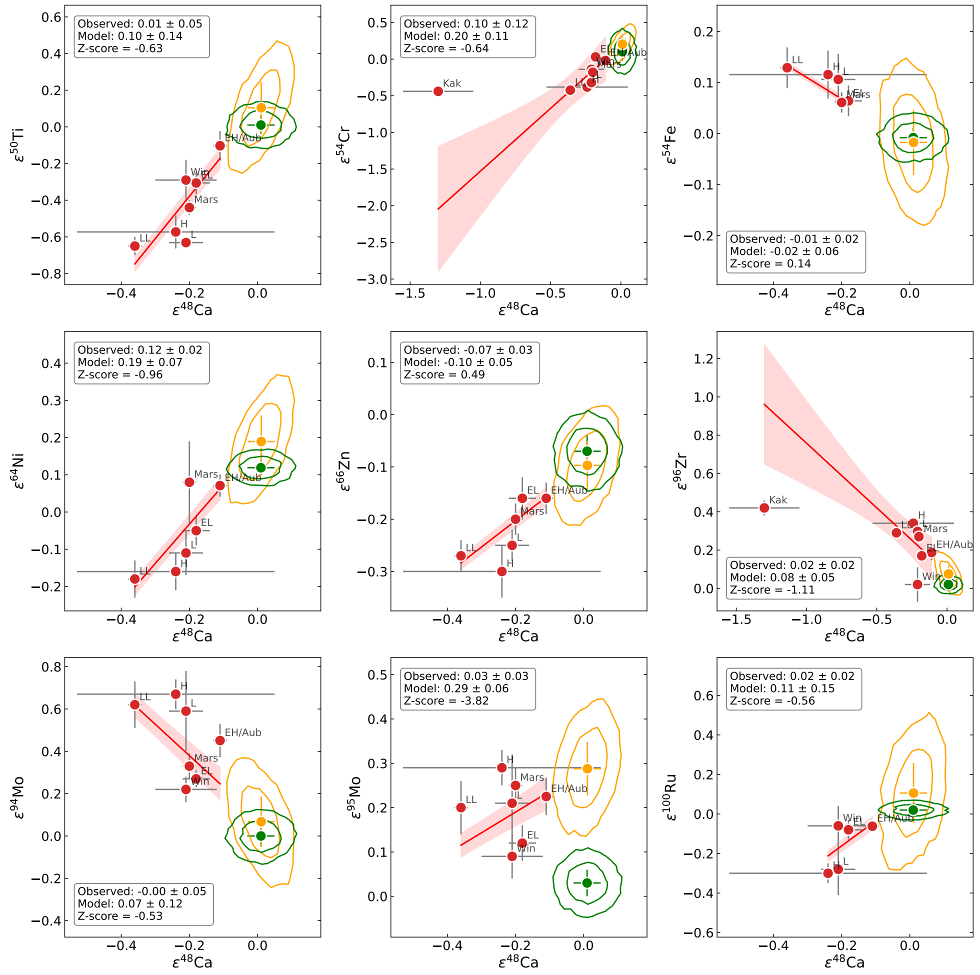

Supplementary Figure 9 Binary $\varepsilon^i x_{pred}$-$\varepsilon^j y$ plots in which the predictor is ε^{48}Ca. Data points denote means and 2-σ uncertainties of analyses of reservoirs belonging to the OC-EC group (red points) and iron meteorites (grey points), with the York regression being shown as the red line with its 2-σ uncertainty envelope (red field). The observed- and predicted composition of the bulk silicate Earth (BSE) are given by the green- and yellow points and their 2-σ uncertainties, respectively. The green- and yellow fields delineate the regions within which 66 % and 95 % of the data lie for the observed- and predicted composition of the BSE, respectively. The caption also shows the Z-score value between the observed- and modelled BSE, computed as Z-score $= (y_{mod} - y_{obs})/\sqrt{\sigma^2_{mod} + \sigma^2_{obs}}$

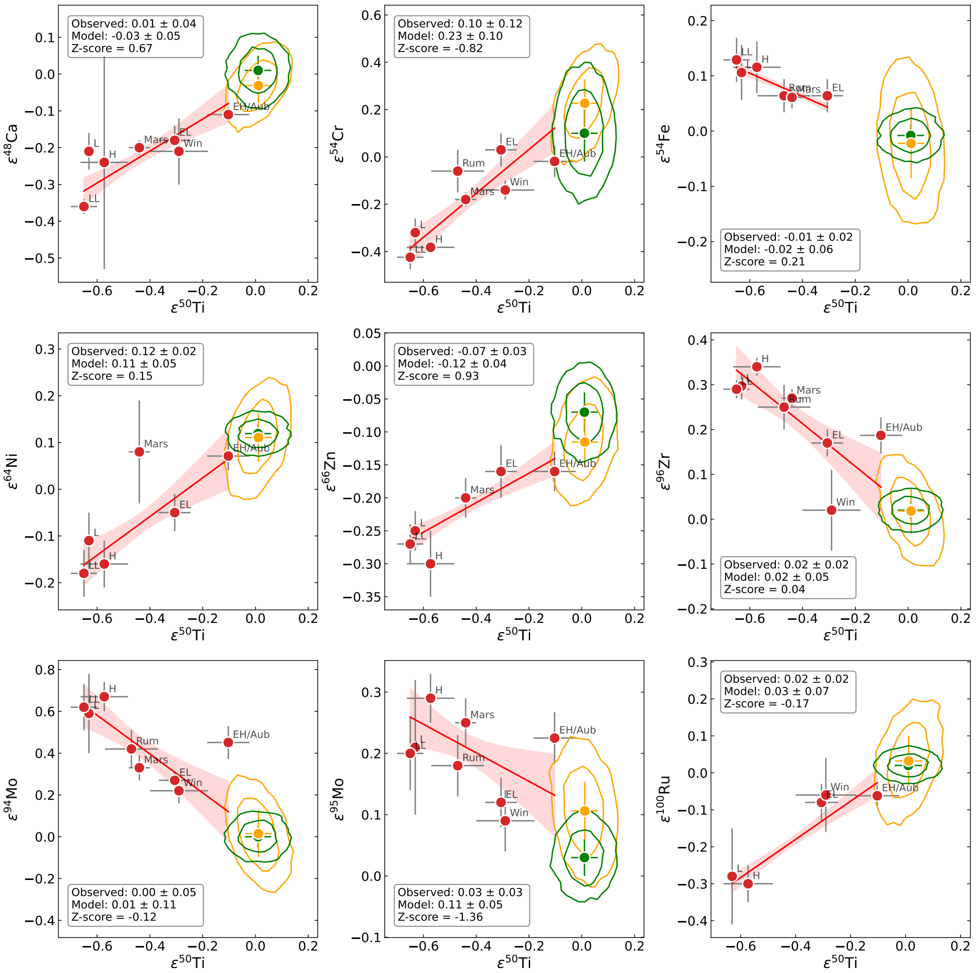

Supplementary Figure 10 Binary $\varepsilon^i x_{pred}$-$\varepsilon^j y$ plots in which the predictor is ε^{50}Ti. Data points denote means and 2-σ uncertainties of analyses of reservoirs belonging to the OC-EC group (red points) and iron meteorites (grey points), with the York regression being shown as the red line with its 2-σ uncertainty envelope (red field). The observed- and predicted composition of the bulk silicate Earth (BSE) are given by the green- and yellow points and their 2-σ uncertainties, respectively. The green- and yellow fields delineate the regions within which 66 % and 95 % of the data lie for the observed- and predicted composition of the BSE, respectively. The caption also shows the Z-score value between the observed- and modelled BSE, computed as $Z\text{-score} = (y_{mod} - y_{obs})/\sqrt{\sigma^2_{mod} + \sigma^2_{obs}}$

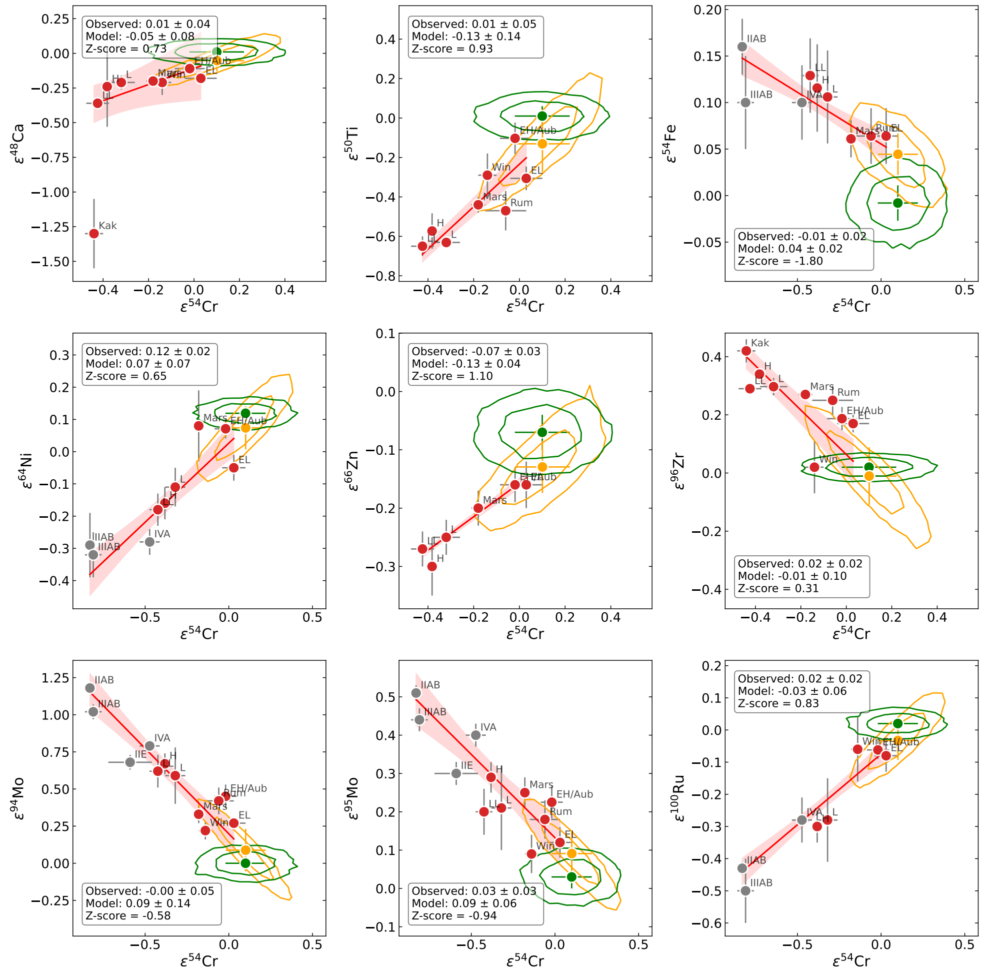

Supplementary Figure 11 Binary $\varepsilon^i x_{pred}$-$\varepsilon^j y$ plots in which the predictor is ε^{54}Cr. Data points denote means and 2-σ uncertainties of analyses of reservoirs belonging to the OC-EC group (red points) and iron meteorites (grey points), with the York regression being shown as the red line with its 2-σ uncertainty envelope (red field). The observed- and predicted composition of the bulk silicate Earth (BSE) are given by the green- and yellow points and their 2-σ uncertainties, respectively. The green- and yellow fields delineate the regions within which 66 % and 95 % of the data lie for the observed- and predicted composition of the BSE, respectively. The caption also shows the Z-score value between the observed- and modelled BSE, computed as $Z\text{-score} = (y_{mod} - y_{obs})/\sqrt{\sigma^2_{mod} + \sigma^2_{obs}}$

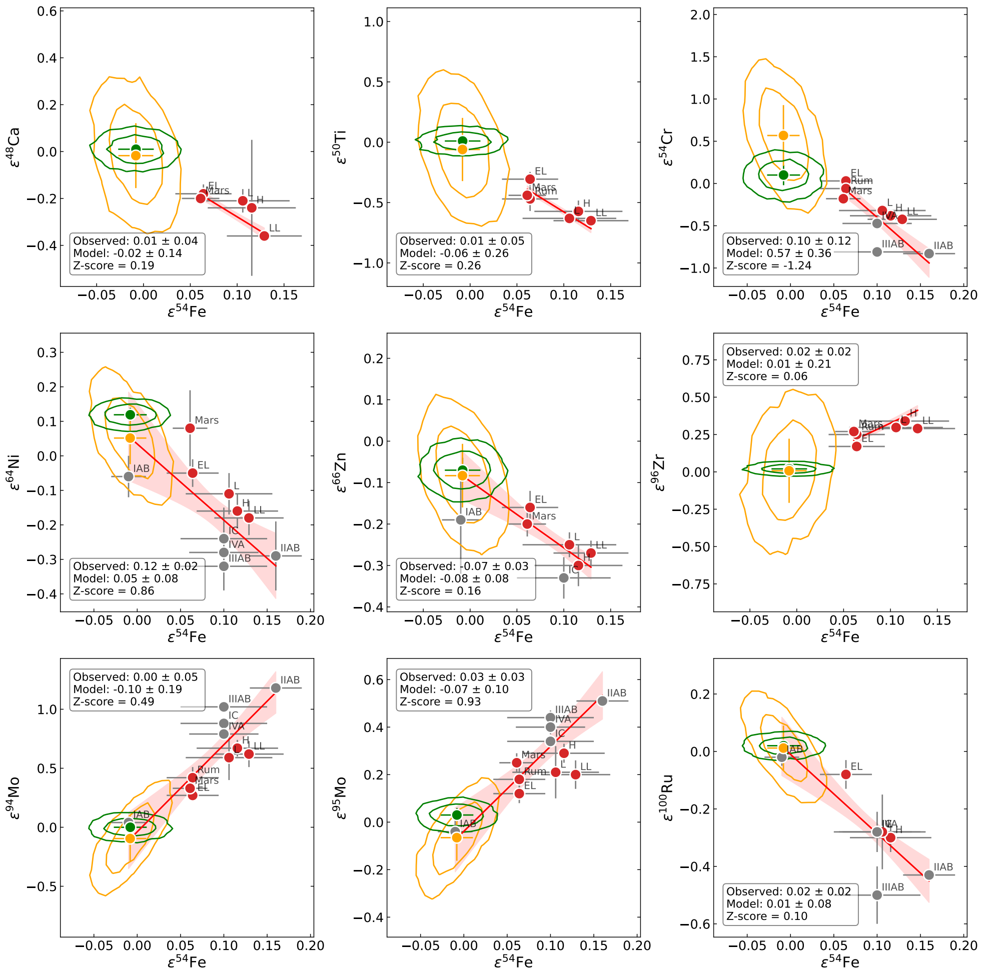

Supplementary Figure 12 Binary $\varepsilon^i x_{pred}$-$\varepsilon^j y$ plots in which the predictor is ε^{54}Fe. Data points denote means and 2-σ uncertainties of analyses of reservoirs belonging to the OC-EC group (red points) and iron meteorites (grey points), with the York regression being shown as the red line with its 2-σ uncertainty envelope (red field). The observed- and predicted composition of the bulk silicate Earth (BSE) are given by the green- and yellow points and their 2-σ uncertainties, respectively. The green- and yellow fields delineate the regions within which 66 % and 95 % of the data lie for the observed- and predicted composition of the BSE, respectively. The caption also shows the Z-score value between the observed- and modelled BSE, computed as $Z\text{-score} = (y_{mod} - y_{obs})/\sqrt{\sigma^2_{mod} + \sigma^2_{obs}}$

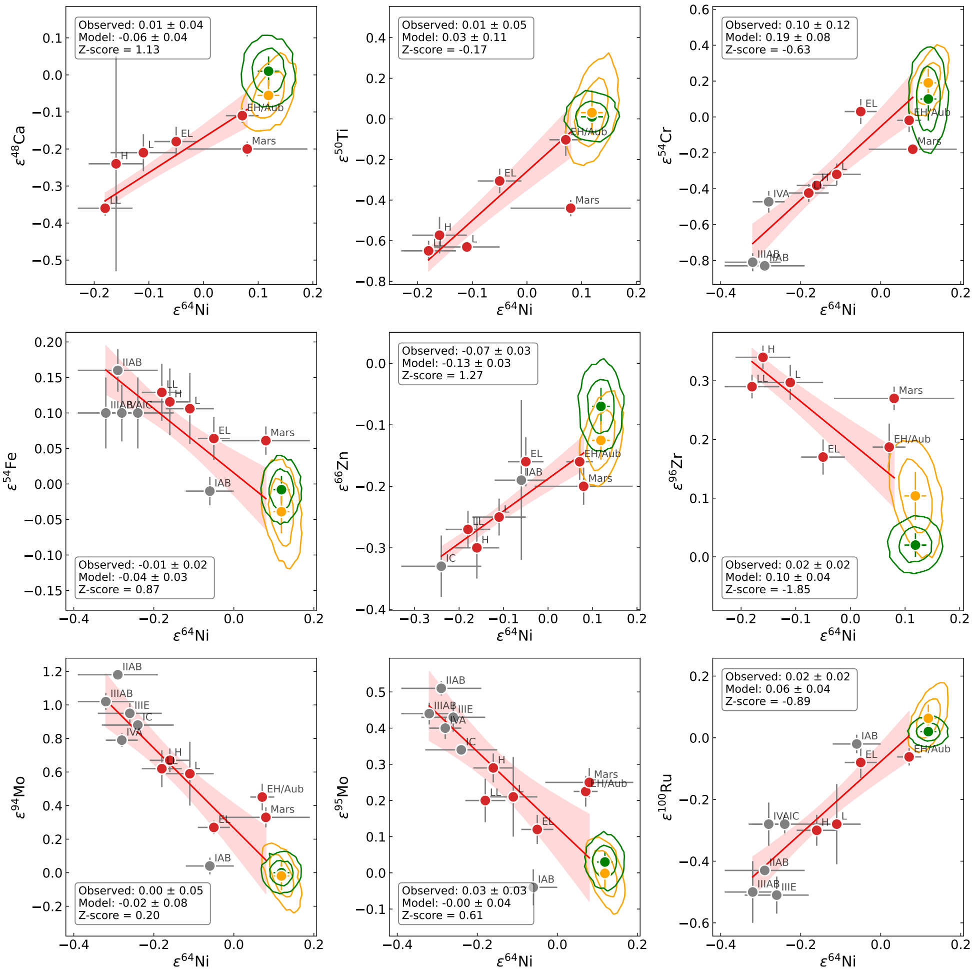

Supplementary Figure 13 Binary $\varepsilon^i x_{pred}$-$\varepsilon^j y$ plots in which the predictor is ε^{64}Ni. Data points denote means and 2-σ uncertainties of analyses of reservoirs belonging to the OC-EC group (red points) and iron meteorites (grey points), with the York regression being shown as the red line with its 2-σ uncertainty envelope (red field). The observed- and predicted composition of the bulk silicate Earth (BSE) are given by the green- and yellow points and their 2-σ uncertainties, respectively. The green- and yellow fields delineate the regions within which 66 % and 95 % of the data lie for the observed- and predicted composition of the BSE, respectively. The caption also shows the Z-score value between the observed- and modelled BSE, computed as $Z\text{-score} = (y_{mod} - y_{obs})/\sqrt{\sigma^2_{mod} + \sigma^2_{obs}}$

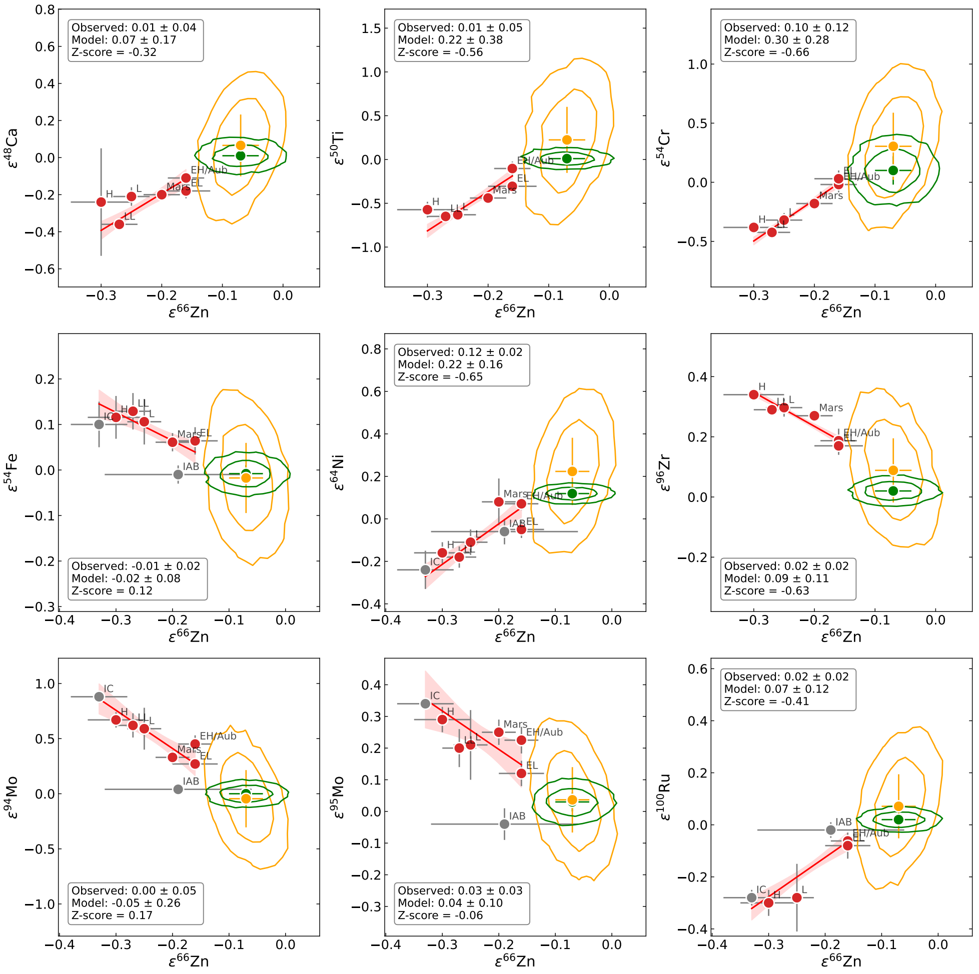

Supplementary Figure 14 Binary $\varepsilon^i x_{pred}$-$\varepsilon^j y$ plots in which the predictor is ε^{66}Zn. Data points denote means and 2-σ uncertainties of analyses of reservoirs belonging to the OC-EC group (red points) and iron meteorites (grey points), with the York regression being shown as the red line with its 2-σ uncertainty envelope (red field). The observed- and predicted composition of the bulk silicate Earth (BSE) are given by the green- and yellow points and their 2-σ uncertainties, respectively. The green- and yellow fields delineate the regions within which 66 % and 95 % of the data lie for the observed- and predicted composition of the BSE, respectively. The caption also shows the Z-score value between the observed- and modelled BSE, computed as $Z\text{-score} = (y_{mod} - y_{obs})/\sqrt{\sigma^2_{mod} + \sigma^2_{obs}}$

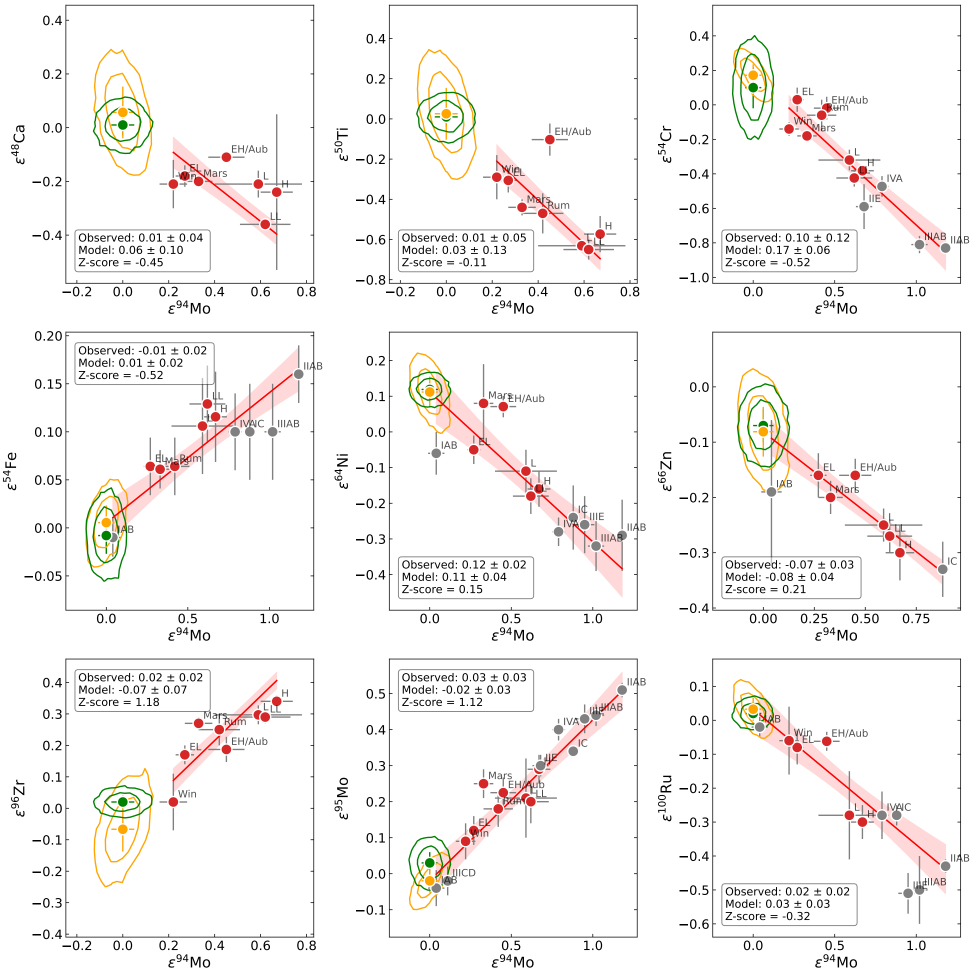

Supplementary Figure 15 Binary $\varepsilon^i x_{pred}$-$\varepsilon^j y$ plots in which the predictor is ε^{94}Mo. Data points denote means and 2-σ uncertainties of analyses of reservoirs belonging to the OC-EC group (red points) and iron meteorites (grey points), with the York regression being shown as the red line with its 2-σ uncertainty envelope (red field). The observed- and predicted composition of the bulk silicate Earth (BSE) are given by the green- and yellow points and their 2-σ uncertainties, respectively. The green- and yellow fields delineate the regions within which 66 % and 95 % of the data lie for the observed- and predicted composition of the BSE, respectively. The caption also shows the Z-score value between the observed- and modelled BSE, computed as $Z\text{-score} = (y_{mod} - y_{obs})/\sqrt{\sigma^2_{mod} + \sigma^2_{obs}}$

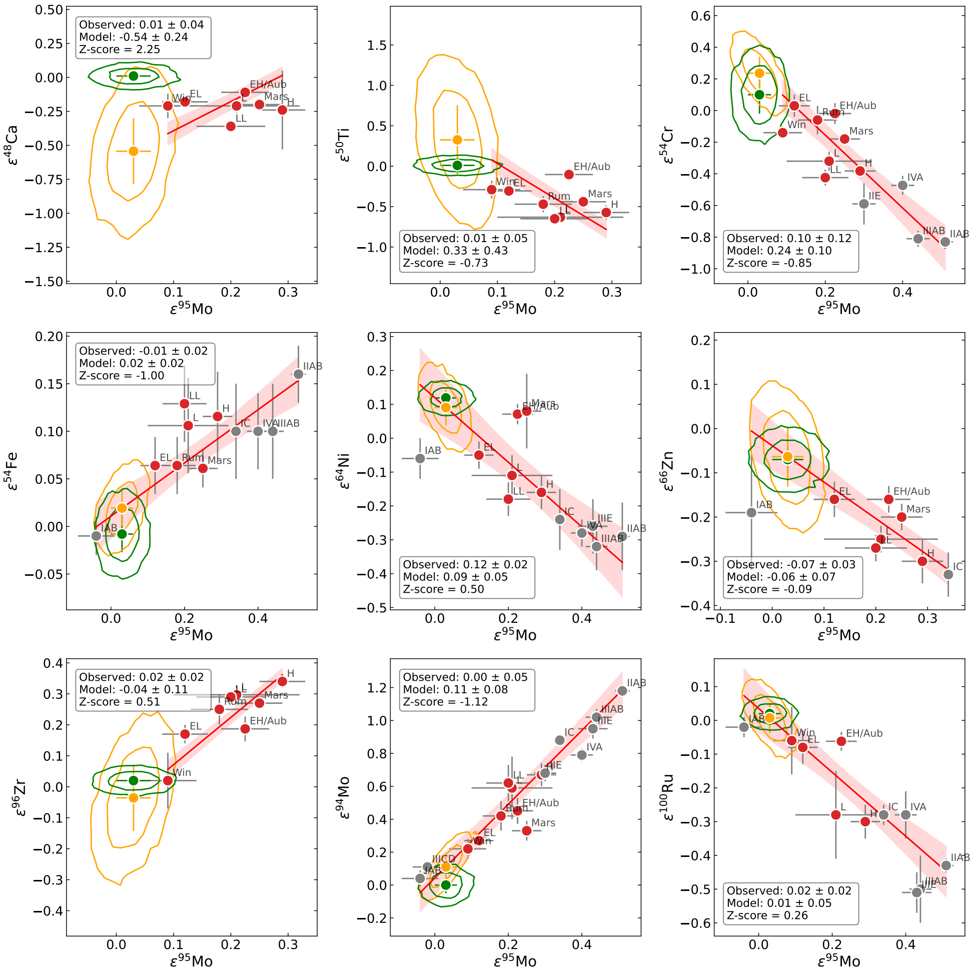

Supplementary Figure 16 Binary $\varepsilon^i x_{pred}$-$\varepsilon^j y$ plots in which the predictor is ε^{95}Mo. Data points denote means and 2-σ uncertainties of analyses of reservoirs belonging to the OC-EC group (red points) and iron meteorites (grey points), with the York regression being shown as the red line with its 2-σ uncertainty envelope (red field). The observed- and predicted composition of the bulk silicate Earth (BSE) are given by the green- and yellow points and their 2-σ uncertainties, respectively. The green- and yellow fields delineate the regions within which 66 % and 95 % of the data lie for the observed- and predicted composition of the BSE, respectively. The caption also shows the Z-score value between the observed- and modelled BSE, computed as $Z\text{-score} = (y_{mod} - y_{obs})/\sqrt{\sigma^2_{mod} + \sigma^2_{obs}}$

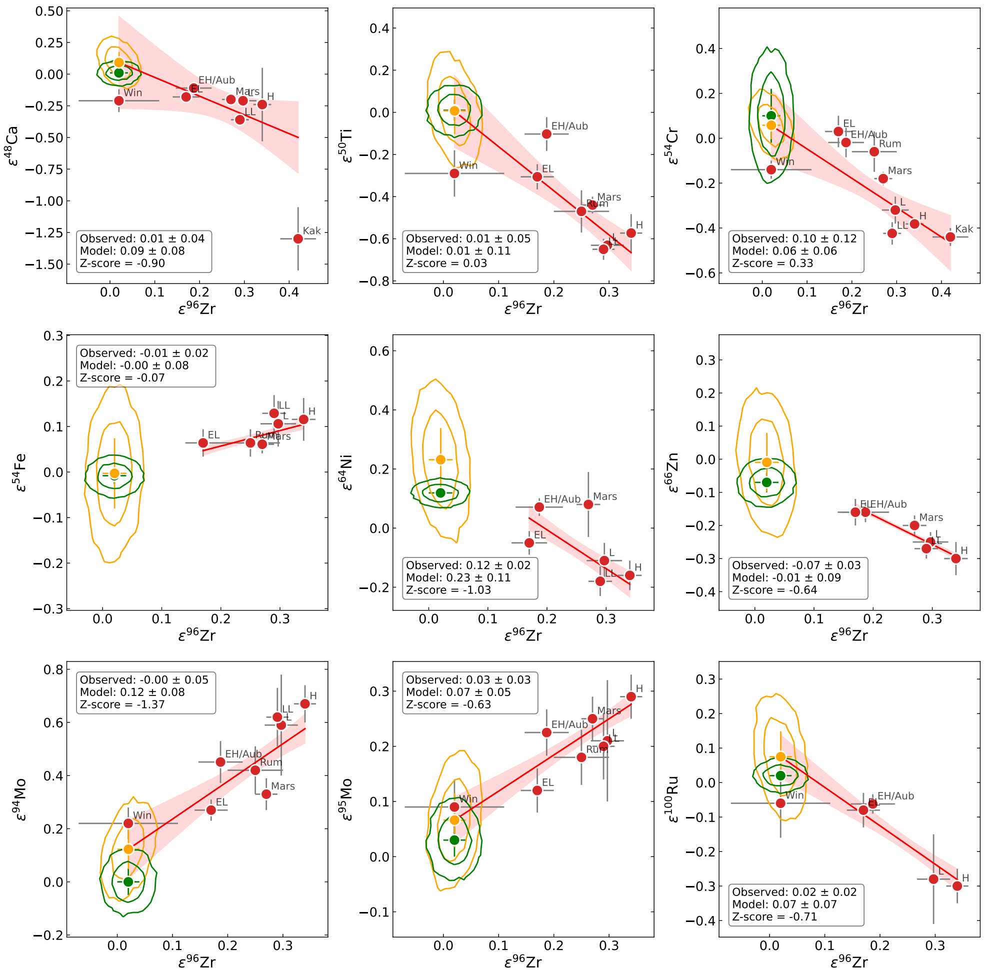

Supplementary Figure 17 Binary $\varepsilon^i x_{pred}$-$\varepsilon^j y$ plots in which the predictor is ε^{96}Zr. Data points denote means and 2-σ uncertainties of analyses of reservoirs belonging to the OC-EC group (red points) and iron meteorites (grey points), with the York regression being shown as the red line with its 2-σ uncertainty envelope (red field). The observed- and predicted composition of the bulk silicate Earth (BSE) are given by the green- and yellow points and their 2-σ uncertainties, respectively. The green- and yellow fields delineate the regions within which 66 % and 95 % of the data lie for the observed- and predicted composition of the BSE, respectively. The caption also shows the Z-score value between the observed- and modelled BSE, computed as Z-score $= (y_{mod} - y_{obs})/\sqrt{\sigma^2_{mod} + \sigma^2_{obs}}$

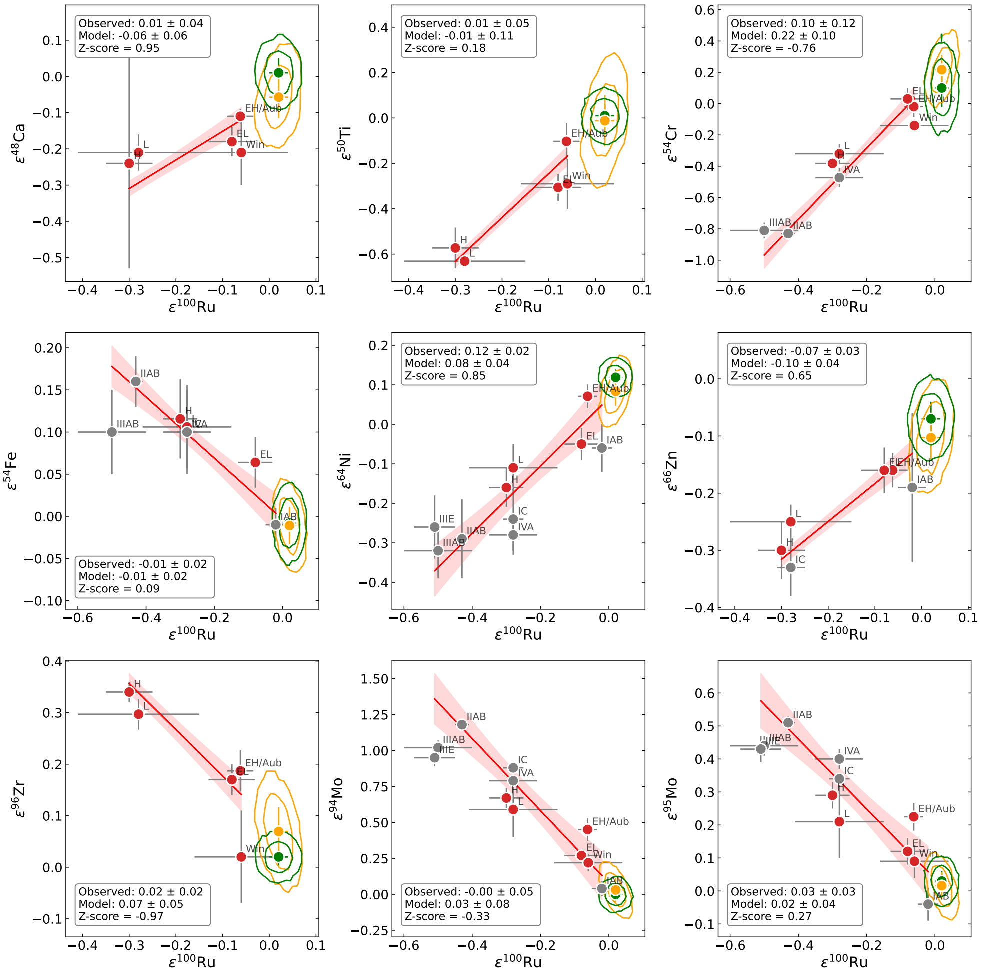

Supplementary Figure 18 Binary $\varepsilon^i x_{pred}$-$\varepsilon^j y$ plots in which the predictor is ε^{100}Ru. Data points denote means and 2-σ uncertainties of analyses of reservoirs belonging to the OC-EC group (red points) and iron meteorites (grey points), with the York regression being shown as the red line with its 2-σ uncertainty envelope (red field). The observed- and predicted composition of the bulk silicate Earth (BSE) are given by the green- and yellow points and their 2-σ uncertainties, respectively. The green- and yellow fields delineate the regions within which 66 % and 95 % of the data lie for the observed- and predicted composition of the BSE, respectively. The caption also shows the Z-score value between the observed- and modelled BSE, computed as $Z\text{-score} = (y_{mod} - y_{obs})/\sqrt{\sigma^2_{mod} + \sigma^2_{obs}}$

4 Mass fraction of CI in the bulk silicate Earth

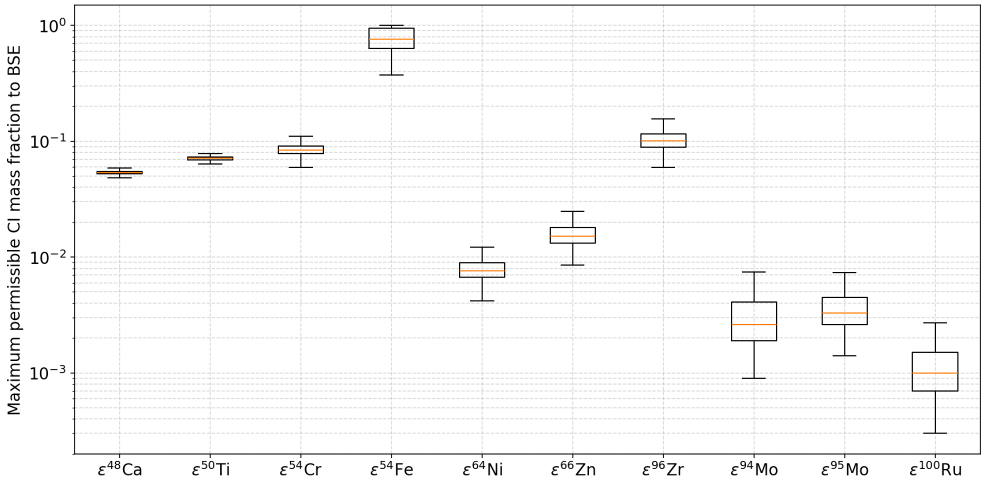

Supplementary Figure 19 A box-and-whisker plot showing maximum permissible mass fraction of CI chondrites in the BSE (f_{CI}), accounting for concentrations in both end-members for each element as calculated in eq. 16. The red line delineates the median value, the limits box the 25th and 75th percentile range and the whiskers the 5th and 95th percentile range calculated from a Monte Carlo simulation of 10^4 iterations.

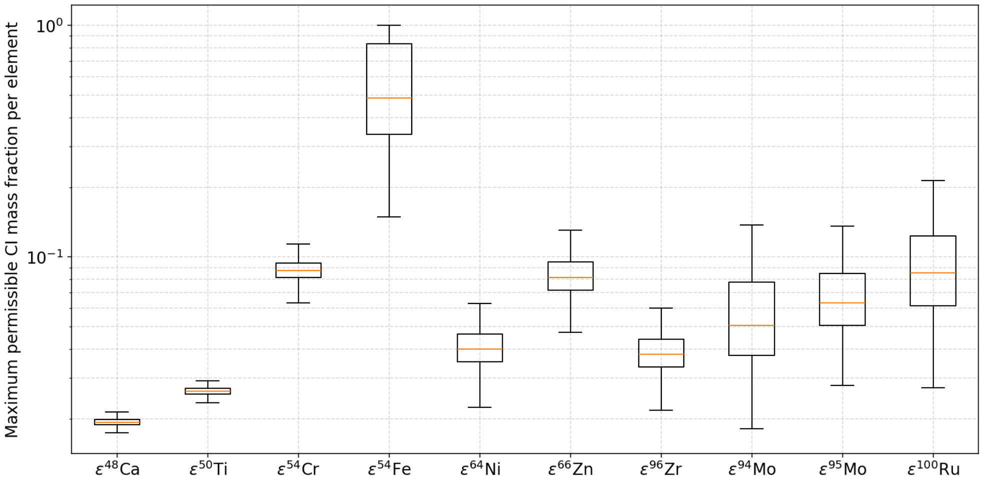

Supplementary Figure 20 A box-and-whisker plot showing maximum permissible mass fraction that CI chondrites comprise of the budget of each element in the BSE ($f_{\mathrm{CI},i}$), as calculated from eq. 18. The red line delineates the median value, the limits box the 25th and 75th percentile range and the whiskers the 5th and 95th percentile range calculated from a Monte Carlo simulation of 10^4 iterations.

5 Prediction of the isotopic compositions of Venus and Mercury

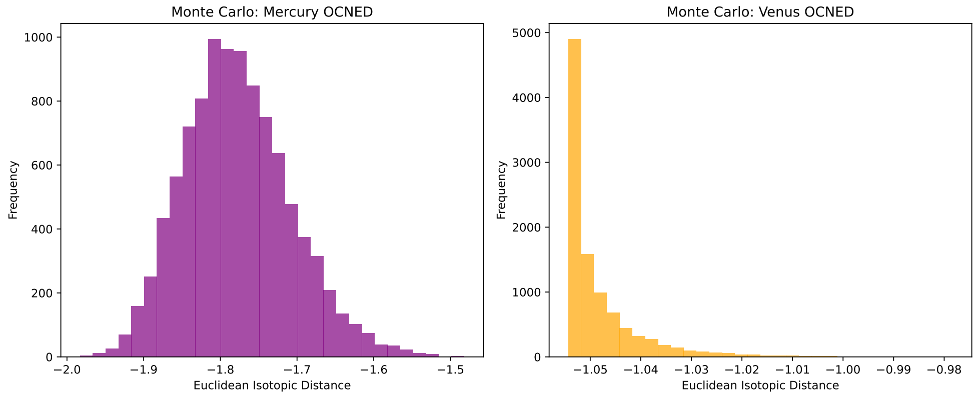

Supplementary Figure 21 Posterior distributions of the Isotopic Euclidean Distances (R_A) of Venus and Mercury as computed from 10^4 Monte Carlo simulations of fits of mass-conserving Gaussians to the R_A values of Earth and Mars (Fig. 3 in the main text), themselves calculated using all 10 isotopic anomalies (Fig. 1a in the main text).

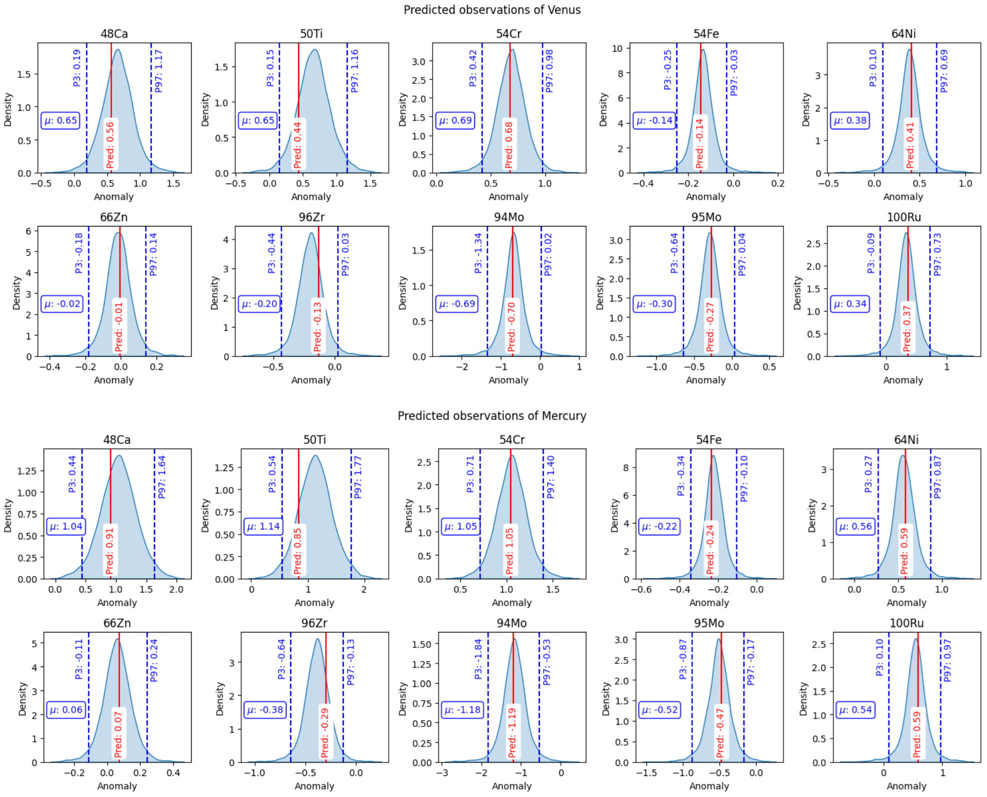

Supplementary Figure 22 Posterior distributions of the predicted isotopic anomalies in each of the 10 isotopic systems for Venus (top panels) and Mercury (bottom panels) as computed from the Bayesian Latent Factor Analysis over 10^4 iterations, including all 10 isotopic anomalies (Fig. 1a in the main text). The vertical blue dashed lines delineate the 3rd and 97th) percentiles of the distribution, respectively, μ the mean isotopic anomaly, and the red vertical line the prediction from deterministic PCA (all expressed in ε units).

Reservoir	Group	Δ^{17}O	ε^{48}Ca	ε^{50}Ti	ε^{54}Cr	ε^{54}Fe	ε^{64}Ni	ε^{66}Zn	ε^{96}Zr	ε^{94}Mo	ε^{95}Mo	ε^{100}Ru
Venus	-	-	0.64 ± 1.14	0.66 ± 0.31	0.69 ± 0.17	-0.13 ± 0.09	0.39 ± 0.23	-0.01 ± 0.13	-0.20 ± 0.21	-0.69 ± 0.46	-0.29 ± 0.27	0.33 ± 0.27
Mercury	-	-	1.04 ± 0.43	1.13 ± 0.37	1.06 ± 0.34	-0.22 ± 0.10	0.56 ± 0.21	0.06 ± 0.11	-0.38 ± 0.22	-1.15 ± 0.75	-0.52 ± 0.37	0.54 ± 0.31

Supplementary Table 9 Predicted Data for Venus and Mercury based on the B-LFA analysis and extrapolation of the 'OC-EC' trend defined in LF1-LF2 space across 10 isotopic anomalies (Fig. 1a in the main text) and the fit to their heliocentric distances (Fig. 3 in the main text). Values are given with their 2σ uncertainties (±).

References

[1] Dauphas, N., Hopp, T. & Nesvorný, D. Bayesian inference on the isotopic building blocks of Mars and Earth. *Icarus* **408**, 115805 (2024).

[2] Budde, G., Tissot, F. L., Kleine, T. & Marquez, R. T. Spurious molybdenum isotope anomalies resulting from non-exponential mass fractionation. *Geochem.* **83**, 126007 (2023).

[3] Albarede, F. *et al.* Precise and accurate isotopic measurements using multiple-collector icpms. *Geochim. Cosmochim. Acta* **68**, 2725–2744 (2004).

[4] Bermingham, K. R. *et al.* The non-carbonaceous nature of Earth's late-stage accretion. *Geochim. Cosmochim. Acta* 38–51 (2025).

[5] York, D., Evensen, N. M., Martınez, M. L. & De Basabe Delgado, J. Unified equations for the slope, intercept, and standard errors of the best straight line. *Am. J. Phys.* **72**, 367–375 (2004).

[6] Burkhardt, C., Hin, R. C., Kleine, T. & Bourdon, B. Evidence for Mo isotope fractionation in the solar nebula and during planetary differentiation. *Earth Planet. Sci. Lett.* **391**, 201–211 (2014).

[7] McCoy-West, A. J. *et al.* Extensive crustal extraction in Earth's early history inferred from molybdenum isotopes. *Nat. Geosci.* **12**, 946–951 (2019).

[8] Hin, R. C. *et al.* The influence of crustal recycling on the molybdenum isotope composition of the Earth's mantle. *Earth Planet. Sci. Lett.* **595**, 117760 (2022).

[9] Budde, G., Burkhardt, C. & Kleine, T. Molybdenum isotopic evidence for the late accretion of outer Solar System material to Earth. *Nat. Astron.* **3**, 736–741 (2019).

[10] Chen, H.-W., Lee, T., Lee, D.-C., Shan, J. J.-S. & Chen, J. C. 48ca heterogeneity in differentiated meteorites. *The Astrophysical Journal Letters* **743**, L23 (2011).

[11] Schiller, M., Bizzarro, M. & Fernandes, V. A. Isotopic evolution of the protoplanetary disk and the building blocks of Earth and the Moon. *Nature* **555**, 507–510 (2018).

[12] Onyett, I. J. *et al.* Silicon isotope constraints on terrestrial planet accretion. *Nature* **619**, 539–544 (2023).

[13] Trinquier, A. *et al.* Origin of nucleosynthetic isotope heterogeneity in the solar protoplanetary disk. *Science* **324**, 374–376 (2009).

[14] Zhang, J., Dauphas, N., Davis, A. M., Leya, I. & Fedkin, A. The proto-earth as a significant source of lunar material. *Nature Geoscience* **5**, 251–255 (2012).

[15] Williams, C. D. *et al.* Chondrules reveal large-scale outward transport of inner Solar System materials in the protoplanetary disk. *Proc. Natl. Acad. Sci. U.S.A.* **117**, 23426–23435 (2020).

[16] Burkhardt, C. *et al.* Terrestrial planet formation from lost inner solar system material. *Sci. Adv.* **7**, eabj7601 (2021).

[17] Rüfenacht, M. *et al.* Genetic relationships of solar system bodies based on their nucleosynthetic Ti isotope compositions and sub-structures of the solar protoplanetary disk. *Geochim. Cosmochim. Acta* **355**, 110–125 (2023).

[18] Trinquier, A., Birck, J.-L. & Allegre, C. J. Widespread 54cr heterogeneity in the inner solar system. *The Astrophysical Journal* **655**, 1179–1185 (2007).

[19] Qin, L., Alexander, C. M. O., Carlson, R. W., Horan, M. F. & Yokoyama, T. Contributors to chromium isotope variation of meteorites. *Geochimica et Cosmochimica Acta* **74**, 1122–1145 (2010).

[20] Yamakawa, A., Yamashita, K., Makishima, A. & Nakamura, E. Chromium isotope systematics of achondrites: chronology and isotopic heterogeneity of the inner solar system bodies. *The Astrophysical Journal* **720**, 150–154 (2010).

[21] Kruijer, T. S., Borg, L. E., Wimpenny, J. & Sio, C. K. Onset of magma ocean solidification on mars inferred from mn-cr chronometry. *Earth and Planetary Science Letters* **542**, 116315 (2020).

[22] Zhu, K., Moynier, F., Schiller, M. & Bizzarro, M. Dating and tracing the origin of enstatite chondrite chondrules with cr isotopes. *The Astrophysical Journal Letters* **894**, L26 (2020).

[23] Zhu, K. *et al.* Late delivery of exotic chromium to the crust of mars by water-rich carbonaceous asteroids. *Science Advances* **8**, eabp8415 (2022).

[24] Schiller, M., Bizzarro, M. & Siebert, J. Iron isotope evidence for very rapid accretion and differentiation of the proto-earth. *Science Advances* **6**, eaay7604 (2020).

[25] Hopp, T., Dauphas, N., Spitzer, F., Burkhardt, C. & Kleine, T. Earth's accretion inferred from iron isotopic anomalies of supernova nuclear statistical equilibrium origin. *Earth and Planetary Science Letters* **577**, 117245 (2022).

[26] Steele, R. C. J., Elliott, T., Coath, C. D. & Regelous, M. Confirmation of mass-independent ni isotopic variability in iron meteorites. *Geochimica et Cosmochimica Acta* **75**, 7906–7925 (2011).

[27] Tang, H. & Dauphas, N. Abundance, distribution, and origin of 60fe in the solar protoplanetary disk. *Earth and Planetary Science Letters* **359**, 248–263 (2012).

[28] Tang, H. & Dauphas, N. 60fe–60ni chronology of core formation in mars. *Earth and Planetary Science Letters* **390**, 264–274 (2014).

[29] Nanne, J. A., Nimmo, F., Cuzzi, J. N. & Kleine, T. Origin of the non-carbonaceous–carbonaceous meteorite dichotomy. *Earth and Planetary Science Letters* **511**, 44–54 (2019).

[30] Cook, D. L., Meyer, B. S. & Schönbächler, M. Iron and nickel isotopes in iid and ivb iron meteorites: Evidence for admixture of an sn ii component and implications for the initial abundance of 60fe. *The Astrophysical Journal* **917**, 59 (2021).

[31] Steller, T., Burkhardt, C., Yang, C. & Kleine, T. Nucleosynthetic zinc isotope anomalies reveal a dual origin of terrestrial volatiles. *Icarus* **386**, 115171 (2022).

[32] Savage, P. S., Moynier, F. & Boyet, M. Zinc isotope anomalies in primitive meteorites identify the outer solar system as an important source of Earth's volatile inventory. *Icarus* **386**, 115172 (2022).

[33] Martins, R., Kuthning, S., Coles, B. J., Kreissig, K. & Rehkämper, M. Nucleosynthetic isotope anomalies of zinc in meteorites constrain the origin of earth's volatiles. *Science* **379**, 369–372 (2023).

[34] Kleine, T., Steller, T., Burkhardt, C. & Nimmo, F. An inner solar system origin of volatile elements in mars. *Icarus* **397**, 115519 (2023).

[35] Paquet, M., Sossi, P. A. & Moynier, F. Origin and abundances of volatiles on mars from the zinc isotopic composition of martian meteorites. *Earth and Planetary Science Letters* **611**, 118126 (2023).

[36] Akram, W., Schönbächler, M., Bisterzo, S. & Gallino, R. Zirconium isotope evidence for the heterogeneous distribution of s-process materials in the solar system. *Geochimica et Cosmochimica Acta* **165**, 484–500 (2015).

[37] Render, J. & Brennecka, G. A. Isotopic signatures as tools to reconstruct the primordial architecture of the solar system. *Earth and Planetary Science Letters* **555**, 116705 (2021).

[38] Render, J., Brennecka, G. A., Burkhardt, C. & Kleine, T. Solar System evolution and terrestrial planet accretion determined by Zr isotopic signatures of meteorites. *Earth Planet. Sci. Lett.* **595**, 117748 (2022).

[39] Poole, G. M., Rehkämper, M., Coles, B. J., Goldberg, T. & Smith, C. L. Nucleosynthetic molybdenum isotope anomalies in iron meteorites–new evidence for thermal processing of solar nebula material. *Earth and Planetary Science Letters* **473**, 215–226 (2017).

[40] Worsham, E. A. *et al.* Distinct evolution of the carbonaceous and non-carbonaceous reservoirs: Insights from ru, mo, and w isotopes. *Earth and Planetary Science Letters* **521**, 103–112 (2019).

[41] Yokoyama, T., Nagai, Y., Fukai, R. & Hirata, T. Origin and evolution of distinct molybdenum isotopic variabilities within carbonaceous and non-carbonaceous reservoirs. *Astrophys. J.* **883**, 62 (2019).

[42] Tornabene, H. A., Ash, R. D., Walker, R. J. & Bermingham, K. R. Genetics, age and crystallization history of group ic iron meteorites. *Geochimica et Cosmochimica Acta* **340**, 108–119 (2023).

[43] Chen, J. H., Papanastassiou, D. A. & Wasserburg, G. J. Ruthenium endemic isotope effects in chondrites and differentiated meteorites. *Geochimica et Cosmochimica Acta* **74**, 3851–3862 (2010).

[44] Fischer-Gödde, M. & Kleine, T. Ruthenium isotopic evidence for an inner Solar System origin of the late veneer. *Nature* **541**, 525–527 (2017).

[45] Bermingham, K. R., Worsham, E. A. & Walker, R. J. New insights into mo and ru isotope variation in the nebula and terrestrial planet accretionary genetics. *Earth and Planetary Science Letters* **487**, 221–229 (2018).

[46] Fischer-Gödde, M. *et al.* Ruthenium isotope vestige of Earth's pre-late-veneer mantle preserved in Archaean rocks. *Nature* **579**, 240–244 (2020).

[47] Hopp, T., Budde, G. & Kleine, T. Heterogeneous accretion of earth inferred from mo–ru isotope systematics. *Earth and Planetary Science Letters* **534**, 116065 (2020).

[48] Greenwood, R., Franchi, I., Gibson, J. & Benedix, G. Oxygen isotope variation in primitive achondrites: The influence of primordial, asteroidal and terrestrial processes. *Geochim. Cosmochim. Acta* **94**, 146–163 (2012).

[49] Haba, M. K., Wotzlaw, J.-F., Lai, Y.-J., Yamaguchi, A. & Schönbächler, M. Mesosiderite formation on asteroid 4 Vesta by a hit-and-run collision. *Nat. Geosci.* **12**, 510–515 (2019).

[50] Keil, K. Brachinite meteorites: Partial melt residues from an FeO-rich asteroid. *Geochem.* **74**, 311–329 (2014). SI: 100 years.

[51] Benedix, G., McCoy, T. J., Keil, K. & Love, S. A petrologic study of the iab iron meteorites: Constraints on the formation of the iab-winonaite parent body. *Meteoritics & Planetary Science* **35**, 1127–1141 (2000).

[52] Wasson, J. & Kallemeyn, G. The iab iron-meteorite complex: A group, five subgroups, numerous grouplets, closely related, mainly formed by crystal segregation in rapidly cooling melts. *Geochim. Cosmochim. Acta* **66**, 2445–2473 (2002).

[53] Ek, M., Hunt, A. C., Lugaro, M. & Schönbächler, M. The origin of s-process isotope heterogeneity in the solar protoplanetary disk. *Nat. Astron.* **4**, 273–281 (2020).

[54] Woosley, S. E. & Heger, A. Nucleosynthesis and remnants in massive stars of solar metallicity. *Physics Reports* **442**, 269–283 (2007).

[55] Dauphas, N. *et al.* Neutron-rich chromium isotope anomalies in supernova nanoparticles. *Astrophys. J.* **720**, 1577 (2010).

[56] Lyons, J. & Young, E. CO self-shielding as the origin of oxygen isotope anomalies in the early solar nebula. *Nature* **435**, 317–320 (2005).

6 Acknowledgments

PAS and DJB were supported by the Swiss State Secretariat for Education, Research and Innovation (SERI) under contract No. MB22.00033, a SERI-funded ERC Starting grant "2ATMO". PAS also thanks the Swiss National Science Foundation (SNSF) through an Eccellenza Professorship (#203668).

7 Author contributions

PAS conceived the study, ran the PCA, developed the multivariate analysis and gaussian fitting model and wrote the paper. DJB developed the PCA and B-LFA analyses and contributed to writing the paper.

8 Additional information

All files cited in the text can be found online as Supplementary Data.
