# Supplementary material for: Homogeneous accretion of the Earth in the inner Solar System
Source: Nat Astron. 2026 Mar 27;10(7):972–9. doi: 10.1038/s41550-026-02824-7 (PMC13379315; doi:10.1038/s41550-026-02824-7)
Supplement: Supplementary file 2 — Peer Review File [file 41550_2026_2824_MOESM2_ESM.pdf]

nature portfolio

Peer Review File

Homogeneous accretion of the Earth in the inner Solar System

Corresponding Author: Professor Paolo Sossi

This file contains all reviewer reports in order by version, followed by all author rebuttals in order by version.

Attachments originally included by the reviewers as part of their assessment can be found at the end of this file.

Version 0:

Reviewer comments:

Reviewer #1

(Comments for the Author)
This is my second viewing of the manuscript. I enjoyed it first time round but demurred over it being sufficiently original for Nature. In resubmission the authors make a vigorous defence of the novelty of their work. While I would quibble that many aspects have been apparent from observations using various individual and paired isotopic systems, the authors make an elegant case for a simple description of solar system variability across numerous isotope ratios. They also make a strong argument for a very small amount of CI component permissible in bulk Earth. As the authors point out in their rebuttal, this has been a topic of some debate in recent literature. Doubtless others, like myself, may feel they are familiar with aspects of the approach, but the manuscript is thought provoking, well-executed and I'm sure will engender debate. Thus I think it would make a suitable contribution to Nature Astronomy. I have made a few very minor comments below

There are some of the traditional formatting issues in a BibTeX reference list

Probably the manuscript is too limited for space and this is not critical, but it might be useful to have a plot of 54Cr vs 50Ti to support visually the points raised in the introduction (and later discussion)

Line 57. Dauphas 17 is cited for the notion of heterogeneous accretion. Although the paper does invoke a change in materials during accretion, the input becomes LESS CC enriched with time, in contrast to most models of heterogeneous accretion. Moreover, I think the main take-home of this paper if that terrestrial accretion is dominated by EC throughout, regardless of the chemical behaviour of isotopic system used (i.e. siderophile to lithophile). Further, Dauphas 17 notes that the change in input materials is not inferred if 142Nd is included in the inversion. So this is a very long winded way of saying I think another reference would be more suitable here.

Line 65 A reference to Martin et al 23 is missing, but part of the near simultaneous trinity of papers on MIF of Zn.

Line 65 the new statement “and evidence for heterogeneity among Ru isotopes exists” doesn't do much to help the reader. Either expand or remove.

Line 67 I know what the authors mean, but I think readers might be confused. Having said the Mo isotopic composition of BSE requires heterogenous accretion (tacitly given the Muenster/Goettingen terrestrial datum) on line 67 the authors then say, in apparent contradiction, that Mo isotopes require NC accretion alone and so is not heterogeneous (which is true, but only if using the Tokyo/Maryland terrestrial Mo datum). This need to be spelt out a bit more clearly.

Line 89 I appreciate the additional uncertainty created by the dependence on mu30Si of correction to common delta30Si of Earth and potential building blocks. However, Dauphas et al 24 have discussed a self-consistent correction and given the authors anyway use the Chicago database for their study, this would make a reasonable starting point. It would be interesting for a comment on what inclusion of Si data does for the model, despite these caveats. I suspect it isn't a big deal, but given Si is a major element, it would be useful to consider these data, despite the caveats.

Line 240 Is 2% CI contribution really ‘insignificant’? It is certainly ‘small’ but this value is more than the fraction of continental

crust or inferred late veneer?
Indeed the late veneer is discussed in next paragraph and a possible non-insignificant CI contribution in this context is consistent with what the authors argue.

Line 274 The authors set up scenario 2 up to (reasonably) sound implausible and hence it seems a bit odd to note that it might be ‘plausible’. I think it would be more appropriate down play the option by saying that while ‘possible’.

(Remarks on code availability)

Reviewer #2

(Comments for the Author)
Review of:
“Homogeneous accretion of the Earth in the inner solar system”
by Sossi & Bower

The paper uses an extensive set of previously published isotope data to place constraints on the material from which the Earth accreted. The authors show by data analyses that the Earth almost exclusively accreted from inner solar system material that is most similar to the compositions of non-carbonaceous (NC) meteorites. They discount previous notions for having a high mass fraction of outer solar system carbonaceous chondrite (CC) material in the accretionary assemblage of Earth.
The results of this paper are very important because they contribute to a recent vibrant discussion about how much CC material from distant regions of the solar system was accreted to Earth. Because there are estimates ranging from <1 % to up to 40 %, depending on which element is used for estimating the CC fraction, the way the authors address this problem by considering isotope data obtained for multiple elements certainly helps to obtain improved constraints on the accretion of the Earth. The results presented in this work will have important constraints for dynamical models of solar system evolution and planetary accretion.

However, before the manuscript could be accepted for publication, it needs proper revision of the points raised in my report (comments below and annotated in the pdf).

1. Mass fraction of CC (or CI) material:
Previously, a high mass fraction of CC material was inferred based on the Mo isotope composition for the Earth (bulk silicate Earth, BSE) reported by Budde et al. (2019). In the study of Budde et al. the Mo isotope composition plotted in between the compositions of NC and CC meteorites and therefore required a mixed NC and CC origin of Mo in the Earth.
In this regard, Sossi and Bower demonstrate in their manuscript that by considering new Mo isotope data from the literature (Bermingham et al., 2025), the new Mo isotope composition inferred for the BSE is different from the one reported by Budde et al. The newly defined Mo isotope composition for the BSE is now consistent with a predominant NC origin of Mo in the BSE and therefore does not support the accretion of a large fraction of CC material as inferred based on the previously reported Mo isotope composition for the BSE by Budde et al. (2019). Budde et al. (2023) later themselves reported that Mo isotope analyses of terrestrial rocks are affected by fractionation effects that cannot be corrected for by using traditionally applied isotope fractionation correction laws for data reduction. That said it is nowhere clear that Earth indeed accreted a large fraction of CC material, especially not if this inference is based on a Mo isotope composition obtained for the Earth that was estimated based on analyses that were affected by analytical artifacts, as shown by Budde et al. (2023).
Reading through the comments provided by reviewers in previous review of this manuscript, it becomes clear that there is a vibrant debate about how much CC material was actually accreted to Earth. A larger CC mass fraction would require specific dynamical models, some of which were recently proposed based on the Budde et al. (2019) Mo isotope composition (e.g. Nimmo et al., 2024; Branco et al., 2025, Icarus). Hence, it is a very important point in the current study of Sossi and Bower that they re-assess and re-determine the Mo isotope composition of the Earth by also considering the latest Mo isotope data reported in the literature (Supplement, Fig. S1).
Nevertheless, I think that the authors focus a bit too much on their point that CC material would be insignificant for the accretion of Earth. First, even if the newly defined Mo isotope composition of the BSE is now consistent with a NC meteorite origin of Mo in the Earth, the uncertainties of the Mo isotope composition of the Earth readily allows for having 10-20 % of Mo derived from CC material. Even if the calculations done by the authors, by considering multiple elements, arrive at the conclusion that only marginal amounts of CC material were accreted, it should be noted and discussed that within uncertainties of the data a certain CC fraction would be permissible and would also be required to account for geochemical observations. The accretion of CC building blocks is supported by isotope data reported for e.g. Se and Te (Varas-Reus et al., 2019; Hellmann et al., 2021) and by relative abundances of S-Se-Te in the Earth’s mantle (Wang and Becker, 2013). Moreover, the D/H ratio of terrestrial surface waters is consistent with a CC origin of the Earth’s water (Alexander et al., 2012; Alexander 2107). So as explained with the next point (2.), there is evidence for why CC material must have been accreted to Earth and that this material is important for Earth to become a habitable planet.

2. Significance of CC material
Reading the manuscript in its present form, I am actually a bit puzzled why there is not much discussion about the origin of

water and other volatile elements on Earth for which there is previously reported evidence that their abundance on Earth would require accretion of carbonaceous chondrite-like (CC) outer solar system material. Even if the mass fraction of CC material that contributed to the Earth's growths is small, CC material remains an important ingredient for the Earth for becoming a habitable planet.
There are a few other constraints for why CC material must have been accreted to Earth. There is evidence from element partitioning (Rubie et al., 2011, 2015), from silver isotopes (Schönbächler et al. 2010), volatile elements (Marty, 2012). The distinct Ru isotope composition of the pre-late veneer mantle in comparison to the modern mantle provides another argument for why CC material was added to the Earth during late accretion (Fischer-Gödde et al., 2020).
In the present form of the manuscript, I don't like how the authors marginalize the significance of CC material for Earth becoming the planet like it is today.
I can understand that they want to claim their point but this should not be done on any expense by not discussing or considering the results and models from previous works. Unravelling the accretion of the Earth remains a challenging task, especially because today we can only investigate the product after some 4.6 billion years of time that passed by. The outcome of different models for the accretion of Earth is variable, depending on the input parameters chosen for the model. The input parameters may change with an increasing amount of new or better data. That said, acknowledging the constraints from previously reported data and models developed from these data for the mixture of materials from which the Earth accreted should be done more carefully in the manuscript.

(Remarks on code availability)

Reviewer #3

(Comments for the Author)
see attached PDF

(Remarks on code availability)
full report included in PDF - Perhaps missing some files?

Version 1:

Reviewer comments:

Reviewer #2

(Comments for the Author)
Review of:
"Homogeneous accretion of the Earth in the inner solar system"
by Sossi & Bower

Revised manuscript

Overall, the authors have sufficiently-well addressed the points raised in review. I have a few additional comments provided below.

Line 62
Please add reference 17 for the NC late veneer constraint.

Line 66:
How does this fit to your model if 30% of Zn in the BSE derives from CC material?

Lines 237-240:
This is not quite correct. Please rephrase this sentence to indicate that the H and N isotope composition of the Earth's surface reservoirs cannot readily be derived from NC-like material alone (see below).

Piani et al. (2020) stated on page 1113 in the last paragraph of their study: "Earth's surficial (oceans and atmosphere) H and N isotopic compositions (deltaD = 0 ‰ and delta15N = 0 ‰) cannot be easily derived from EC materials alone because they are richer in D and 15N by ~100 to 200 ‰ and 5 to 40 ‰, respectively."
"We find a ~4% contribution from CI-like material to Earth's surficial reservoir based on hydrogen abundances and isotopic compositions and a ~15% contribution based on equivalent data for nitrogen."

These estimated amounts of CI-like material are not trivial. Therefore, these data refer to a larger fraction of the Earth's accretionary material. Merely stating that the H and N budget could solely be derived from NC meteorite-like material falls too short here.

General comment about fraction of CC material:
There are many studies in which a carbonaceous chondrite contribution among the Earth's building blocks is proposed. Some of these studies also use constraints from isotope compositions (either mass-dependent or mass-independent) of elements in the bulk silicate Earth. Other evidence is based on relative abundances of siderophile and chalcophile elements in the BSE (e.g., Wang and Becker, 2013; Braukmüller et al., 2019; Mezger et al., 2021).

It is fine if with your model you arrive at a different probably more provoking conclusion, but I think it is good scientific conduct to also refer to the constraints from prior studies and compare them with your new results. Reading through the manuscript I sometimes feel that this could be done more properly, e.g. by adding a sentence in the conclusion section that opposed to the new model presented by the authors, previous studies based on X elements or Y isotopes provided evidence for accretion of CC material.

I will leave this point to the authors about how they want their paper to be perceived by the community.

Other than that, I think the revised version of the manuscript is much improved and after thorough consideration of the above-mentioned points is well-suited for publication in Nature Astronomy.

Cologne, January 2026

Mario Fischer-Gödde

(Remarks on code availability)

Open Access This Peer Review File is licensed under a Creative Commons Attribution 4.0 International License, which permits use, sharing, adaptation, distribution and reproduction in any medium or format, as long as you give appropriate credit to the original author(s) and the source, provide a link to the Creative Commons license, and indicate if changes were made.
In cases where reviewers are anonymous, credit should be given to 'Anonymous Referee' and the source.
The images or other third party material in this Peer Review File are included in the article's Creative Commons license, unless indicated otherwise in a credit line to the material. If material is not included in the article's Creative Commons license and your intended use is not permitted by statutory regulation or exceeds the permitted use, you will need to obtain permission directly from the copyright holder.
To view a copy of this license, visit https://creativecommons.org/licenses/by/4.0/

Homogeneous accretion of the Earth in the inner solar system

Response to Reviewers #2

Legend
Reviewer comments in normal face.
Our responses in bold face. NB: Line numbers refer to the non-track changes version of the manuscript file.

Reviewer #1 (Comments for the Author):

This is my second viewing of the manuscript. I enjoyed it first time round but demurred over it being sufficiently original for Nature. In resubmission the authors make a vigorous defence of the novelty of their work. While I would quibble that many aspects have been apparent from observations using various individual and paired isotopic systems, the authors make an elegant case for a simple description of solar system variability across numerous isotope ratios. They also make a strong argument for a very small amount of CI component permissible in bulk Earth. As the authors point out in their rebuttal, this has been a topic of some debate in recent literature. Doubtless others, like myself, may feel they are familiar with aspects of the approach, but the manuscript is thought provoking, well-executed and I'm sure will engender debate. Thus I think it would make a suitable contribution to Nature Astronomy. I have made a few very minor comments below

Response: We thank the reviewer for the positive assessment of our work!

There are some of the traditional formatting issues in a BibTeX reference list.
Response: Thanks, we have done our best to fix these now.

Probably the manuscript is too limited for space and this is not critical, but it might be useful to have a plot of 54Cr vs 50Ti to support visually the points raised in the introduction (and later discussion).
Response: We agree, and this suggestion was also made by Reviewer #2. In an ideal world, we would add this plot, but, given that this is a standard figure that the interested reader can find in other papers on isotopic anomalies, and we are restricted for space, we have to prioritise our new findings and, regretfully, are not able to add such a figure.

Line 57. Dauphas 17 is cited for the notion of heterogeneous accretion. Although the paper does invoke a change in materials during accretion, the input becomes LESS CC enriched with time, in contrast to most models of heterogeneous accretion.

Moreover, I think the main take-home of this paper if that terrestrial accretion is dominated by EC throughout, regardless of the chemical behaviour of isotopic system used (i.e. siderophile to lithophile). Further, Dauphas 17 notes that the change in input materials is not inferred if 142Nd is included in the inversion. So this is a very long winded way of saying I think another reference would be more suitable here.

Response: Yes, the reviewer is absolutely right. We have now rephrased our wording in this section and modified the references (L. 57-59). The Dauphas [2017] conclusions have now been largely superseded by Dauphas et al. [2024], in which they argue, much like Nimmo et al. [2024], that the Earth accreted largely EC (NC) material (95 %) up to ~75 % of its mass, followed by 80 % EC and 20 % CC up to 99.5 %, before reverting back to 95 % EC for the final 0.5 % of accretion.

Line 65 A reference to Martin et al 23 is missing, but part of the near simultaneous trinity of papers on MIF of Zn.

Response: Thank you, yes, we have now added the reference to Martins et al. [2023] (L. 63).

Line 65 the new statement "and evidence for heterogeneity among Ru isotopes exists" doesn't do much to help the reader. Either expand or remove.

Response: Indeed, we now dedicate a sentence to explaining that there is evidence for a change in the ε^{100}Ru composition of Archean vs. modern-day terrestrial rocks. See new L. 63-65.

Line 67 I know what the authors mean, but I think readers might be confused. Having said the Mo isotopic composition of BSE requires heterogenous accretion (tacitly given the Muenster/Goettingen terrestrial datum) on line 67 the authors then say, in apparent contradiction, that Mo isotopes require NC accretion alone and so is not heterogeneous (which is true, but only if using the Tokyo/Maryland terrestrial Mo datum). This need to be spelt out a bit more clearly.

Response: Yes, we can see that the reasoning might not have been sufficiently explicit here. We have reworded this state that the Mo isotopic estimate was re-assessed by the Tokyo/Maryland groups. See L. 66-69.

Line 89 I appreciate the additional uncertainty created by the dependence on mu30Si of correction to common delta30Si of Earth and potential building blocks. However, Dauphas et al 24 have discussed a self-consistent correction and given the authors anyway use the Chicago database for their study, this would make a reasonable starting point. It would be interesting for a comment on what inclusion of Si data does for the model, despite these caveats. I suspect it isn't a big deal, but given Si is a major element, it would be useful to consider these data, despite the caveats.

Response: Ordinarily, we would agree with the reviewer and examine the corrected data point reported by [Dauphas et al., 2024]. They apply a correction to the μ^{30}Si value measured by Onyett et al. [2023], from +12±3 to +5±3, yielding a new difference between EH and Earth of

+4±3, which Dauphas et al. [2024] claim is barely resolvable.
However, in this case, it is still not clear whether the data for enstatite chondrites as corrected by Dauphas et al. (2024) are accurate, because a second group at IPGP has reported contrasting results in an abstract in LPSC (https://www.hou.usra.edu/meetings/metsoc2025/pdf/5250.pdf). In it, they report μ^{29}Si for enstatite chondrites of +12.7±3.0, $n = 5$, which is clearly distinguished from the bulk silicate Earth, +1.4±2.6 ppm. This is counter to the conclusions drawn from the corrected data of Dauphas et al. (2024). The authors go on to note that "Notably, our results differ significantly from the data reported by Onyett et al. (2023)."
Given the lack of consensus in the data of the same meteorite group (enstatite chondrites) measured by different groups, combined with the fact that our analysis already covers 10 different systems, we feel it would be premature and perhaps counterproductive to include Si isotopes in our analysis. We refrain from any further comment at this stage.

Line 240 Is 2% CI contribution really 'insignificant'? It is certainly 'small' but this value is more than the fraction of continental crust or inferred late veneer? Indeed the late veneer is discussed in next paragraph and a possible non-insignificant CI contribution in this context is consistent with what the authors argue.

Response: Yes, we could have been more precise here. We have now expanded on this section to give two possible limits (L. 233-239); the first is the 2 % figure, which is derived from Ca isotopes. Since Ca is a refractory lithophile element, the BSE contribution is the same as the bulk Earth (BE) contribution. The second is the maximum recorded in the BSE, which is given by the $<$0.1 % CI material allowed from Ru isotopes. We have also appealed to volatile element abundances (H, C, N) to show that CI chondrites could not represent more than 0.1 % the mass of the BSE. The two constraints can be reconciled if CI material is accreted during ongoing core formation, such that amounts of H, C, N and Ru are extracted from the mantle into the core. However, these are all upper limits, and we highlight that lower CI fractions are permissible.

Line 274 The authors set up scenario 2 up to (reasonably) sound implausible and hence it seems a bit odd to note that it might be 'plausible'. I think it would be more appropriate down play the option by saying that while 'possible'.

Response: Yes, we have changed this to 'possible', and we now also note, following comments from Reviewer #2, that K and Ru isotopes provide some evidence of possible heterogeneous accretion, depending on the mass fraction of the mantle that the Isua rocks are assumed to represent.

Reviewer #2 (Comments for the Author):

Review of: "Homogeneous accretion of the Earth in the inner solar system" by Sossi & Bower

The paper uses an extensive set of previously published isotope data to place constraints on the material from which the Earth accreted. The authors show by data analyses that the Earth almost exclusively accreted from inner solar system material that is most similar to the compositions of non-carbonaceous (NC) meteorites. They discount previous notions for having a high mass fraction of outer solar system carbonaceous chondrite (CC) material in the accretionary assemblage of Earth.

The results of this paper are very important because they contribute to a recent vibrant discussion about how much CC material from distant regions of the solar system was accreted to Earth. Because there are estimates ranging from <1 % to up to 40 %, depending on which element is used for estimating the CC fraction, the way the authors address this problem by considering isotope data obtained for multiple elements certainly helps to obtain improved constraints on the accretion of the Earth. The results presented in this work will have important constraints for dynamical models of solar system evolution and planetary accretion.

However, before the manuscript could be accepted for publication, it needs proper revision of the points raised in my report (comments below and annotated in the pdf).
Response: We thank the reviewer for their clear and positive summary, and for the constructive comments that follow.

1. Mass fraction of CC (or CI) material:

Previously, a high mass fraction of CC material was inferred based on the Mo isotope composition for the Earth (bulk silicate Earth, BSE) reported by Budde et al. (2019). In the study of Budde et al. the Mo isotope composition plotted in between the compositions of NC and CC meteorites and therefore required a mixed NC and CC origin of Mo in the Earth.

In this regard, Sossi and Bower demonstrate in their manuscript that by considering new Mo isotope data from the literature (Bermingham et al., 2025), the new Mo isotope composition inferred for the BSE is different from the one reported by Budde et al. The newly defined Mo isotope composition for the BSE is now consistent with a predominant NC origin of Mo in the BSE and therefore does not support the accretion of a large fraction of CC material as inferred based on the previously reported Mo isotope composition for the BSE by Budde et al. (2019). Budde et al. (2023) later themselves reported that Mo isotope analyses of terrestrial rocks are affected by fractionation effects that cannot be corrected for by using traditionally applied isotope fractionation correction laws for data reduction. That said it is nowhere clear that Earth indeed accreted a large fraction of CC material, especially not if this inference is based on a Mo isotope composition obtained for the Earth that was estimated based on analyses that were affected by analytical artifacts, as shown by Budde et al. (2023).

Reading through the comments provided by reviewers in previous review of this manuscript, it becomes clear that there is a vibrant debate about how much CC material was actually accreted to Earth. A larger CC mass fraction would require specific dynamical models, some of which were recently proposed based on the Budde et al. (2019) Mo isotope composition (e.g. Nimmo et al., 2024; Branco et al., 2025,

Icarus). Hence, it is a very important point in the current study of Sossi and Bower that they re-assess and re-determine the Mo isotope composition of the Earth by also considering the latest Mo isotope data reported in the literature (Supplement, Fig. S1).

Response: Thank you, we agree with these points, and highlight that we use both the Budde et al. [2023] and Bermingham et al. [2025] (which are consistent with one another) to derive a new bulk silicate Earth value.

Nevertheless, I think that the authors focus a bit too much on their point that CC material would be insignificant for the accretion of Earth. First, even if the newly defined Mo isotope composition of the BSE is now consistent with a NC meteorite origin of Mo in the Earth, the uncertainties of the Mo isotope composition of the Earth readily allows for having 10-20 % of Mo derived from CC material. Even if the calculations done by the authors, by considering multiple elements, arrive at the conclusion that only marginal amounts of CC material were accreted, it should be noted and discussed that within uncertainties of the data a certain CC fraction would be permissible and would also be required to account for geochemical observations. The accretion of CC building blocks is supported by isotope data reported for e.g. Se and Te (Varas-Reus et al., 2019; Hellmann et al., 2021) and by relative abundances of S-Se-Te in the Earth's mantle (Wang and Becker, 2013). Moreover, the D/H ratio of terrestrial surface waters is consistent with a CC origin of the Earth's water (Alexander et al., 2012; Alexander 2107). So as explained with the next point (2.), there is evidence for why CC material must have been accreted to Earth and that this material is important for Earth to become a habitable planet.

Response: Yes, we completely agree that, given the uncertainties in the nucleosynthetic isotopic ratios, our model shows that *some* CC material is permissible, but is likely to be very low (now quantified as <0.1 % in the BSE or <2 % in the bulk Earth). We emphasise that material is *not* allowed at mass fractions higher than this, lest the linear trends among the 'OC-EC subgroup' of the NC meteorites break down. We dedicated a discussion to this possibility (see also Methods under "Permissible fraction of CI material in the BSE"). We expand on this to be more explicit on L. 233-239.
On the stable isotope data such as Se and Te, as well as the D/H ratio, Piani et al. [2020] showed that the bulk silicate Earth's H isotopic composition is consistent with EH-like material, and not only CIs (now cited on L. 238). Furthermore, it should be noted that the use of simple elemental ratios or stable isotope ratios (e.g., δ^{15}N) are far less reliable indicators of the provenance of Earth-forming material, as they can be fractionated by mass transfer processes (such as core formation or evaporation). Therefore, these ratios may suggest differences in provenance, but cannot be used as direct evidence of CC material in the BSE in the same way the nucleoynsthetic isotope ratios can.

2. Significance of CC material

Reading the manuscript in its present form, I am actually a bit puzzled why there is not much discussion about the origin of water and other volatile elements on Earth for which there is previously reported evidence that their abundance on Earth would require accretion of carbonaceous chondrite-like (CC) outer solar system material. Even if the mass fraction of CC material that contributed to the Earth's growths is small, CC material remains an important ingredient for the Earth for becoming a habitable planet. There are a few other constraints for why CC material must have been accreted to Earth. There is evidence from element partitioning (Rubie et al., 2011, 2015), from silver isotopes (Schönbächler et al. 2010), volatile elements (Marty, 2012). The distinct Ru isotope composition of the pre-late veneer mantle in comparison to the modern mantle provides another argument for why CC material was added to the Earth during late accretion (Fischer-Gödde et al., 2020). In the present form of the manuscript, I don't like how the authors marginalize the significance of CC material for Earth becoming the planet like it is today. I can understand that they want to claim their point but this should not be done on any expense by not discussing or considering the results and models from previous works. Unravelling the accretion of the Earth remains a challenging task, especially because today we can only investigate the product after some 4.6 billion years of time that passed by. The outcome of different models for the accretion of Earth is variable, depending on the input parameters chosen for the model. The input parameters may change with an increasing amount of new or better data. That said, acknowledging the constraints from previously reported data and models developed from these data for the mixture of materials from which the Earth accreted should be done more carefully in the manuscript.

Response: We agree that understanding the source of volatile elements to the Earth is an important consequence of our work, and that we dedicated essentially no space to this in our earlier submission. We now add a sentence on this point on L. 235-236, which states that the vast majority of Earth's volatiles are therefore likely to be endemic to the inner solar system.

As for our response to the previous comment, our approach is to examine only nucleosynthetic isotope anomalies, whose variations are tracers of material provenance. The other lines of evidence cited by the reviewer for the accretion of CC material are not based on these nucleosynthetic isotope ratios, and thus, the interpretations derived therefore are non-unique with respect to provenance. Nevertheless, for completeness, we treat them here individually:

1) Rubie et al. [2011, 2015]: These papers indicate that the first ∼70% of mass accretion to Earth was reducing, while the latter 30% was more oxidising, in order to match the abundances of Ni, Mo and W, in particular, in the Earth's mantle. A fundamental assumption is to equate reducing material with NC material and oxidising with CC material. Grewal et al. [2024] showed that the fO2s of iron meteorite parents bodies are all

relatively oxidising, despite that they show both NC and CC affinities. Therefore, it is not required that NC material be reducing. Second, Siebert et al. [2013] showed that homogeneous accretion is possible at relatively high pressures/temperatures of core formation due to incorporation of O in the metallic phase. The Rubie model assumes CI-like abundances of the major rock-forming elements (Mg, Si, Fe, Cr, Ni, Co). At the time, CI chondrites were thought to be a good approximation for the composition of material that formed the Earth, but subsequent studies showed that non-carbonaceous chondrites were more likely candidates, and our present study indicates that the Earth does not require any chondritic material at all. Since the abundances vary among chondrites [Yoshizaki and McDonough, 2021], how this assumption affects the conclusions of Rubie et al. [2011] remains untested.

2) Silver isotopes - Schönbächler et al. [2010]. They argue that the Earth's Ag isotopic composition is consistent with a CC contribution that was added late in Earth's accretion. The isotopic data are consistent with this interpretation, yet, Schönbächler et al. [2010] favour this conclusion due to the inference that moderately volatile element abundances appear to be consistent with a CC origin - a popular empirical conclusion at the time. However, since the Earth's composition (ε^{107}Ag = -2.2 ± 0.7, 2SD) overlaps with NC meteorites, Sharps (H3.4, -1.7 ± 0.7) or Ucera (H5, -2.2 ± 0.6), an NC origin for the BSE Ag isotope composition is equally permissible. Similarly, the Ag isotopic composition of the Earth's mantle also depends on factors that do not trace provenance, such as the time at which the core segregated, and the Earth's Pd/Ag ratio.

3) Volatile elements - Marty [2012]. The work of Marty [2012] supports a chondritic contribution, but not necessarily carbonaceous chondrites. Indeed, they note *"the average D/H and 15N/14N compositions of chondrites (excluding enstatite chondrites which do not contain significant amounts of water) are undistinguishable from the terrestrial surface inventory (Fig. 2)"*. Subsequently, Piani et al. [2020] showed that enstatite chondrites *do* have enough H, and, moreover have similar N and D/H ratios to the bulk silicate Earth. More recently, Marty [2022] concluded that *"The hydrogen and nitrogen isotopic signatures of mantle rocks and minerals are consistent with an EC-like contribution whereas those of heavy noble gases are still too imprecise to conclude."* Other views exist [e.g., Alexander, 2017], but this serves to underscore that there is no consensus that can be drawn on the provenance of volatiles from stable isotope or elemental abundance ratios alone.

On the Ru isotopic composition of the Earth's mantle, we acknowledge that some CC material is permitted (but not required), because Archean rocks show different ε^{100}Ru to modern-day igneous rocks. That is, the

Archean rocks from Isua [Messling et al., 2025] are even more *s*-process-enriched than are modern-day rocks. However, equally, this difference may be due to differences in the accretion of NC material, and hence does not require CC material. Furthermore, we argue that, because the fraction of the Earth's mantle that these rocks represent is unknown, it cannot be assumed that they were representative of the entire mantle. We make this point more explicitly in the manuscript on new L. 240-253. See response to detailed comment on L. 242-250, below.

Detailed (in-line) comments on the pdf:

L. 23: This would be great to compute how this would translate into actual isotope anomalies that would be expected for these planets. I mean real epsilon values instead of Euclidian Isotopic Distances shown in Fig. 3. Maybe a table in hte Supplement with the predicted anomalies would help.

Response: Yes, definitely, great idea! We now back-compute the ε values from the Isotopic Euclidean Distances, as suggested by the reviewer. See section new Supplementary Figure 22 and Table S9.

L. 33 - 36: For the interested reader, it would be good to show a figure of e54Cr vs. e50Ti to better illustrate what you are referring to here. Especially for non-isotope experts this would help a lot. Also because you use this plot to explain the different fractions of CC material depending on which CC group is chosen for the mixture.

Response: As also suggested by Reviewer #1, we agree it would help with comprehension. However, this plot is standard in many isotopic anomaly papers since 2011, and, given the space constraints in Nature Astronomy, we have to prioritise our new findings.

L. 41 - 43: This discussion would aslo benefit from a figure as mentioned above.

Response: See above.

L. 45 - 46: Or heterogeneous accretion models (Rubie et al., 2011, 2015; Schönbächler et al., 2010, Marty, 2012) in which it was proposed that CC material was accreted towards the final stages of Earth's main accretion including the late veneer.

Response: Thanks for the suggestions! We now add these references in the next paragraph in which we explicitly discuss heterogeneous accretion (L. 57).

L. 51: In case of Ru the positive e100Ru anomalies identified in Archean mantle-derived rocks extend the spectrum towards s-process enriched building blocks (Fischer-Gödde et al., 2020). These data are direct evidence for building blocks that are not represented in the meteorite record. The positive e100Ru value inferred for the pre-late veneer mantle composition shoes that the Earth accreted from material that is more enriched in s-process nulcides. All meteorites, as in case of Mo, show s-process depleted compositions. But only for Ru an s-process enriched composition was identified among the terrestrial rock record. The sentence stating that other elements (Zr, Ru, Nd)

needs to be rephrased to account for s-process enriched building blocks as evidenced by Ru isotopes. Assuming that the Hadean mantle of the Earth had an e100Ru value of +0.22, using extrapolations in Fig. S18 you can make predictions what the isotope compositions of other elemetns would be. Taking into account the distinct s-proces enriched composition of the Archean mantle in comparison to the modern mantle composition, how do you explain the difference in the context of your model? And how do you explain the shift from the Archean/Hadean mantle composition to the modern mantle composition? One way would be by accretion of CC material. That's at least how it was explained in our previous study in 2020.

Response: Thank you for the comments, we understand the reviewer's point, but it is not entirely correct. Our text, as written (that the BSE's Mo, Zr, Ru and Nd isotope composition is an *s*-process-enriched end-member) is accurate. This is evident in the work of Burkhardt et al. [2016]; Render et al. [2022] and the Fischer-Gödde et al. [2020] paper cited by the reviewer. Furthermore, in our paper, we show that these isotopic anomalies are all correlated, and hence reflect the same material. The reviewer is right in that Ru isotopic evidence indicates that there is a component even more extreme than this and is "not represented in the meteorite record" - but - as we argue - neither is the composition of the Earth! This is one of the key new findings of our work.

The second point regarding how we explain the shift in Archean to modern mantle composition of the Earth is treated later in the manuscript. Briefly, we favour an explanation that the Archean value [Messling et al., 2025], which is from one location (Isua metaperidotites) is not representative of the bulk Archean mantle, given that it is impossible to determine the mass fraction of the mantle reservoir from which it derived. We now acknowledge that this is not the only interpretation, and addition of less *s*-process-rich material to the mantle over time could be an alternative explanation (as argued by Messling et al. [2025], we now mention this on L. 248-250), but this would mean that the consistency with the present-day lithophile anomalies is fortuitous. Moreover, this material need not be CC (although it is permitted), since other NC meteorites are also poorer *s*-process nuclides than is the present-day BSE [see Fischer-Gödde and Kleine, 2017].

L.55- 58: This is certainly not a new suggestion and was already proposed in much earlier studies (Rubie et al., 2011, 2015; Schönbächler et al., 2010, Marty, 2012). I am pretty sure that ref. 9 (Dauphas, 2017) did not propose that the accreted material became more CC like over time, especially not for the second half of Earth's accretion that was suggested to be dominated by enstatite chondrite-like NC material.

Response: Yes, the reviewer is right on both counts. As mentioned above, we now cite these studies as having suggested the Earth accreted heterogeneously.

Indeed, the Dauphas [2017] study reports that the first 60 % of accretion contained 50 % enstatite chondrite-like and the final 40 % was 100 % enstatite chondrite-like. This is largely superseded by the Dauphas et al. [2024] model, which essentially shows the same thing as the recent Nimmo et al. [2024] model (i.e., 95 % EC material in the first 75 % of accretion, 80 % EC from 75 - 99.5 % and back to 95 % EC for the last 0.5 % of mass accretion to the Earth - late veneer). We have updated the text to accurately reflect this more nuanced view (L. 57-59).

L. 57: Another possibility suggested by Budde et al. 20219 was that the Moon-forming impactor was a CC-type body. However, this is solely based on an Mo isotope argument. As your assessment in Fig. S1 shows, the Mo isotope composition of Budde et al., 2019 was estimated based on samples that were affected by residual mass fractionation effects that could not be corrected for. That's why it is important to re-determine the Mo isotope composition of the BSE, e.g. like you have done in the supplement.

Response: Yes, we agree that there are a number of ad-hoc models that, as we show, are not consistent with the data. Thanks for mentioning it!

L. 60: How about ruthenium ?? The distinct e100Ru compositions of the modern BSE and the early Archean (pre-late veneer) mantle clearly show that this was the case (Fischer-Gödde et al., 2020; Messling et al., 2025). In fact, Ru is so far the only element for which we have direct isotopic evidence, not Mo!

Response: Yes, fair enough, even though we did mention ε^{100}Ru in the next sentence, we see how it would be worthwhile to cite up-front. We have now revised this paragraph to be more precise on this issue, as also suggested by Reviewer #1. In particular, we distinguish between heterogeneous accretion (for which Ru isotopes provide some evidence) and heterogeneous accretion of CC material (which is a distinct issue). See updated L. 64-66.

L. 61 - 62: The problem here is that the Mo isotope composition of the BSE as determined in the study of Budde et al 2019 is affected by analytical artifacts as the authors themselves describe in a later study (Budde et al., 2023). However, instead of using the BSE Mo isotope composition of Budde et al 2019 there are also other studies reporting Mo isotope composition for the BSE like Yokoyama et al 2019 and Bermingham et al. 2025. That said, I much prefer taking these new data into account for re-determinng the Mo isotope composition of the BSE like you have done in the supplement (Fig. S1). It would be more wise to rephrase this sentence on a way that a more accurate Mo isotope composition of the BSE would substantially reduce the fraction of permitable CC material.

Response: Thank you, we agree. Although going into the details is perhaps too technical for the introduction, we have rephrased this sentence as the reviewer suggests, in that the two competing estimates of Budde et al. [2023] and Yokoyama et al. [2019]; Bermingham et al. [2025]

imply uncertainty in the Mo isotopic composition (which we subsequently update in our paper, as mentioned by the reviewer). See updated L. 66-69.

L. 67: First, even if the Mo isotope composition is more consistent with an NC origin for most of the Mo, the values of e94Mo and e95Mo inferred for the BSE composition will come with an uncertainty. This uncertainty would easily allow for having 10 to 20% of CC derived Mo in the BSE.

Response: Yes, we agree that there is an uncertainty associated with the amount of CC material permitted (see response above and our section "Permissible fraction of CI material in the BSE" in the Methods and Supplementary Information. However, here, we think it is an issue of semantics - we state that the *need* for CC material is obviated. That is, it is not needed within current uncertainties, but a small amount is allowed (see our new L. 238-239). We have rephrased this to be more explicit. See L. 66-69.

L. 76: Why not listing all the isotope values used for the modelling in this parantheses?

Response: Thanks, we list them all just 6 lines later. Here now we delete any reference to them and just state we use 10 isotope systems. See updated L. 76.

L. 84 - 87: As stated in my comment above accurately determining the Mo isotope composition of the BSE is a major challenge but very important to do carefully because depending on the detailed Mo composition, the CC fraction required for the Earth can vary drastically. So taking these new data into account is really important, especially because the earlier data by Budde were affected by analytical artifacts.

Response: Thank you, yes, we agree!

L. 87 - 89: I don't think there is ONLY one explanation for the origin of the 17O variations. I would rephrase this sentence to express that the self-shielding is not the only possible explanation, plus it is not at all clear if this would be the most likely theory for explaining the oxygen isotope variations.

Response: Self-shielding is the most widely accepted explanation, though we take the point that it may not be the only process leading to mass-independent O isotope variations. We now update L. 89-91.

L. 172 - 176: Discussion of pre-late veneer and pre-giant impact signatures? Here, and in the context of the following sentences, you might want to add a sentence that the status of Earth being an NC end-membder composition is even more evident in the light of new e40K and e100Ru anomalies identified in Archean rocks that are distinct from the compositions of the modern BSE and any meteorite group (Wang et al., 2025, Nature Geosci; Fischer-Gödde et al, 2020).

Response: Thanks, indeed, we have now added these references here. See updated L. 174. The implications are discussed later in the manuscript.

L. 221-223: Referring to my comment above about e40K and e100Ru, please mind that the positive (s-process enriched) e100Ru value inferred for the pre-late veneer mantle composition is different from both modern BSE and any meteorites. This is direct evidence for the missing NC component!!

Response: Yes, we are aware of this. We now explicitly state we use the present-day composition in our analysis (L. 88-89). As will become clear, we have an alternative interpretation for the 'pre-late veneer' composition.

L. 235 - 236: What do you want to express with this sentence? Assuming a mixture of a BSE compnente with concnetrations that are identical to CI chondrite? This does sound very odd to me. Please clarify or better explain.

Response: For clarity, we have rephrased this as follows: "For binary mixing between isotopically CI- and BSE-like end-members, both with equivalent concentrations of each element...". L. 227-229.

L. 240: Especially in the light of the discussion around the origin of water and other volatile elements on Earth, the question arises how an 'insignificant' fraction of 0.3 to 2 % CI or CC material could make a huge difference for having water on Earth?

Response: Yes, a very fair comment (see also response to Reviewer #1). We emphasise that these are *maximum* values (new L. 233-235). We also now highlight that accretion of more than 1, 0.3 and 0.1 % CI material, respectively, would exceed the H, C and N contents of the BSE, allowing us to constrain the likely amount of CI material to less than about 0.1 %.

L. 242-250: Here, I strongly disagree. As mentioned in my comment above the positiv e100Ru value inferred for the pre-late veneer mantle shows that s-process enriched building blocks contributed to the Earth'S accretion. These building blocks were even more enriched in s-process nuclides as the modern BSE. This data cannot be neglected in your paper by simply saying this material did not significantly contribute to the accretion of Earth. Also, if positiv e100Ru values in OIBs sources originate from a core with a positive e100Ru value, as recently suggested (Messling et al., 2025), this demonstrates that the core of the Earth is also s-process enriched compared to the modern BSE compositioen. If the core is enriched in s-process Ru nuclides, this cannot be generated from the accretion of a minor insignificant accretionary component. This issue needs proper revision and more careful assessment.

Response: We take the point that the data of Messling et al. [2025] suggests some degree of heterogeneous accretion - we stated as much in our earlier submission, we wrote that this "...implies some heterogeneous accretion to the Earth". However, the rest of the line of argumentation is speculation. Neither the reviewer (nor anyone else) can be sure to what extent the Isua rocks are representative of the whole of the Archean mantle. Likewise, we cannot be sure that they are an insignificant accretionary component. We simply present our interpretation as an equally viable alternative to that of Messling et al. [2025]. For transparency, we now also mention the Messling et al. [2025] interpretation (see new L. 242-244).

We add that, if their interpretation is correct, then this would imply the position of the present-day BSE ε^{100}Ru composition at the end of linear extensions of the OC-EC trend defined by lithophile element isotopes would necessarily be a coincidence (see new L. 248-250). Therefore, we favour our interpretation.

L. 259: If Messling et al. (2025) is corret with their interpreation that OIB sources inherited positive e100Ru values from the Earth's core this assumption would not be correct.

Response: Point taken, we have added a caveat here (L. 261).

L. 280-282: Getting a bit tired to mention but the difference in e100Ru between the Archean pre-late veneer mantle composition and the modern BSE composition shows that the Earth accreted from a continuum of compositions.

Response: Thanks, but, as mentioned above, this is an interpretation. We have now discussed it and given the appropriate credit (see comment L. 242-250), which we hope is sufficient for the reviewer.

L. 285: Please refer here to previous studies in which A heliocentric zoning of nucleosynthetic Ru and Mo isotope anomalies was proposed (Render et al, 2017 and Fischer-Gödde and Kjleine, 2017).

Response: Good point, we have now mentioned them here. Note that these studies are also mentioned later in the manuscript (old L. 292).

L. 301-302: Again, in case of Ru, based on the s-process enriched signature inferred for the Archean mantle composition and the heliocentirc zoning of e100Ru isotope anomalies, it was suggested in Fischer-Gödde et al., 2020 that such s-process enriched building material was present in the inner solar system closer to the sun where it was accreted by Venus and Mercury.

Response: True, we have now also added the Fischer-Gödde et al. [2020] reference in the appropriate section (L. 291). The new result of our work is actually to pin down the precise isotopic compositions of Venus and Mercury, rather than to indicate they could lie somewhere to the s-process enriched side of the NC array for heavy elements, as has been done in the past.

Fig. 3: With your model you could predict how the epsilon values (anomalies) for the various elements you considered would like like foir ech element for Mercury, Venus and MArs? From an isotope perspective this would be very interesting to know. It might be worth to include a table in the supplement in which you make predictions for the isotope compositons of other terrestrial planets.

Response: Yes, great thought, this is correct. This has now been added - see response to comment on L. 23.

L. 311-312: This scenario would probably need a reference?

Response: This is just an idea for now - there is no specific reference for this statement.

L. 319-320: I agree, it would be great if they did that.
Response: Agreed!

Supplementary Information

Table S1: What do the uncertainties reflect here? 2 sd, 2 se ? Please state clearly.
Response: This has now been added (2 se).

Table S1: It is not ok to not give credit to previous studies in which these data were reported. I think in hte manuscript you mention that you are using data that were compiled in a previous study. In my view it is not ok merely citing a study that had compiled all this data from the literature. Proper credit must be given to the studies in which the original data were reported.
Response: Yes, we agree. This cannot be done in the main text due to reference restrictions, but we now add some key references to the supplementary material to this end.

Page 7. As suggested in one of the previous reviewer comments, why not using average OC and EC here ? Is there any element that shows a resolvable difference between H-L-LL or EH-EL?
Response: We already demonstrated in our previous response to reviewers that i) Mathematically, there is no difference in the regressions when the analyses are grouped or ungrouped, because we use weighted averages and the York regression that takes into account errors in x and y, and ii) Yes, there are differences between EH-EL and hence they cannot be grouped. We refer the reviewer to our earlier response. Titanium isotopes are a prime example of this (see Zhang et al. [2012]; Rüfenacht et al. [2023]). No changes made.

Fig. S4. What do the uncertainties on the regressions reflect? How were they calculated?
Response: Thanks for bringing this up - we did not specify this in the caption. These are York regressions and their associated 2σ uncertainties.

Reviewer #3 (Comments for the Author):

Sossi and Bower (review) Reviewed by Greg Brennecka (w/ code checking help from someone in the group).

Summary: This is a great paper overall. I have very few criticisms of the work, and I commend the authors on a very nice study. My comments are basically just ways I think the authors can clarify a few things for the general reader and some minor suggestions. Very good stuff and, other than all the math, a pleasure to read :)

Specifics:
Line 26: There is no doubt that the Trinquier et al. 2009 paper is a seminal work in cosmochemistry, but I don't think it is the correct reference here. The NC/CC dichotomy, to my reading, wasn't really recognized in print until Warren in 2011. Following that paper, there are myriad refs you could use, such as works like Budde et al. 2016, Kruijer et al. 2017, and perhaps the review by Bermingham et al. 2020 to direct people to other work/readings on the subject. Trinquier et al. 2009 deserves a ton of credit, but probably not on the dichotomy in my opinion, despite what the authors might now claim they wrote about in that important 2009 paper.

Response: Yes, a point well made, we have now simply referenced the work of Warren [2011], and we build up the story sequentially with more recent papers (including the Kruijer et al. [2017] paper mentioned) cited later in the sentence.

Line ∼55: I think the discussion of the percent of CC material making up Earth is good and needed, but they way it is currently written could be misconstrued that 40% of the mass of Earth, when, it is of course 40% of the sampled Mo is from CC – because the rest is inaccessible and in the core. I am certain the authors understand this, and it is worded properly as they do (L55: "the fraction of Mo derived. . . "), but given this is a general interest journal, I am not sure it will come through to folks not fully immersed in the topic. I would suggest it could help to make this clearer what is actually being claimed by the Mo, Zn, et al. studies. Again, this is not a knock on the author's current phrasing or anything, just a suggestion to make it more understandable to those that are not experts in the topic already.

Response: Thanks for the tips, we have now reworded this for clarity.

Line 140: Perhaps consider including Shollenberger et al. (2025-EPSL) and Rego et al. (2025-GPL) here, they come to similar conclusions based on isotopes from major elements from Ryugu and other CIs. The Rego paper was likely published after submission of your manuscript, but both papers provide strong support and discussion for a separate CI/Ryugu reservoir. Not critical, but would be helpful for the argument here.

Response: Thank you for bringing these papers to our attention. Indeed, the Shollenberger et al. [2025] paper was published after our initial submission in March, and the Siciliano Rego et al. [2025] paper after we submitted our revisions. We now cite them on L. 137. We are also heartened to see that the PCA of Shollenberger et al. [2025] shows very similar results to those presented in our work!

Figure 1 and associated: No agenda or important question here, but I am curious why the authors prioritized 64Ni over 62Ni or 60Ni. All show variation, and 60Ni can behave differently than 64 (or 62, which are usually well correlated). And since 94Mo and 95Mo were both chosen, I was just curious as to why only one Ni isotope was included. Something to consider, and it might change Ni a bit if 60 is used instead/in

addition. Of course the decay and input from 60Fe might need to be considered in some cases, but something to think about.

Response: Yes, this is a fair question. As the reviewer rightly mentions, ε^{64}Ni and ε^{62}Ni are correlated [Tang and Dauphas, 2012; Nanne et al., 2019]. In fact, as noted in our Supplementary Table S1, we use a combination of ε^{64}Ni and ε^{62}Ni and convert the latter into ε^{64}Ni by the relationship ε^{64}Ni=(2.84±0.31)ε^{62}Ni (see Table S1). The linear correlation between ε^{64}Ni and ε^{62}Ni spans both NC and CC groups, and is hence unlike that seen between ε^{94}Mo and ε^{95}Mo, which are only linearly correlated *within* NC and CC groups, but displaced relative to one another, meaning both should be considered. Our analysis is therefore independent of whether ε^{64}Ni or ε^{62}Ni is used.

The reason not to use ε^{60}Ni is two-fold; i) the abundance of ^{60}Ni is not only nucleosynthetic in origin, but also depends upon the decay of ^{60}Fe. Although the ^{60}Fe/^{56}Fe ratio is very low (10^{-8}, Tang and Dauphas [2012]), this may influence the ε^{60}Ni at the ppm-level, depending on the accretion time and Fe/Ni ratio. ii) The entire range of ε^{60}Ni variations in chondrites and iron meteorites [e.g., Nanne et al., 2019] are of the order of ~10-15 ppm, making them only somewhat larger than possible radiogenic variability and close to the analytical precision [±4ppm Nanne et al., 2019]. Consequently, we use ε^{64}Ni where the anomalies are largest and thus best analytically resolved.

Fig. 1 and associated: Very interesting about the ureilites. . . is it worth some more discussion as to why this is the case? If you are positing the CI are a separate reservoir, then it would stand to reason that the ureilites would be a fourth, right? This is of course addressed in the nice work by Rufenacht et al. 2023, but without the statistical rigor you are presenting here. I would consider expanding on this a bit, perhaps.

Response: Yes, we agree this is something to follow-up on. In the interest of space, we do not add any further comments to the text. We note here, however, that the ureilites are also special for two other reasons i) they show heterogeneities in ε^{50}Ti, ε^{54}Cr, Δ^{17}O [e.g., Kruttasch et al., 2025] and thus cannot be considered to come from a homogeneous parent body, and ii) their Δ^{17}O compositions fall on the CCAM (Carbonaceous Chondrite Anhydrous Mineral Line) with a slope of 1, unlike any NC body, but similar to CI Chondrites. Therefore, treating the ureilites in any detail would entail a more detailed discussion, certainly worthy of future investigation.

Fig. 2: Just a thought, but perhaps it would be easier to see if you also used different shades of gray/green in addition to circle square. Cool figure, but not easy to read at first glance and I think that will help. Also, most of the values are BARELY within uncertainty (e.g., Ni, Zn, Ca, Zr?, Fe?), so this is not a slam dunk, but cool, no doubt.

Response: At the moment, we think the figure is fine as-is, as the important point to highlight here is simply the lithophile vs. siderophile and iron-peak vs. heavy element distinction, but the identity of the specific element is secondary, since they all fall on the line, within uncertainty.

Line 274: I don't understand what "1" points to here.
Response: Thanks, good catch, this was meant to refer to the Results section. We have removed this sentence in any case to save space.

Reviewer #3 (Remarks on code availability):
Code check: It was asked to check the code, so here are some notes about the downloadable files specifically: There are four folders that were provided in the raw materials. A description of run issues is provided below for each of the four folders.

10634_0_source_data_130915_t11b23/
This folder only contains data and does not have any code to run.
Response: N/A.

10634_0_source_data_4713666_stl3bb/
Assuming the user knows how to set up the proper environment, this code runs fine. Two plots are produced, but only one is saved. The unsaved figure looks different from what's in the supplied manuscript, but effort was only put into execution, not reproduction.
Response: Thanks for confirming that the code functions. Yes, the output of the code yields the Multivariate Linear Regression analyses for one single predictor element (x_{pred}). Fig. 2 in the paper is constructed by cycling through each of the 10 isotope systems as predictors and then taking a weighted average.

10634_0_source_data_4713667_stl3bc/
Assuming the user knows how to set up the proper environment, this code runs fine. The figure mostly matches the one in the supplied manuscript.
Response: Thanks for confirming that the code functions.

code_pca/
The code in this folder could not be run.

1. No README.md file was provided with the code, which means the code cannot be installed properly using Poetry. This was easily fixed, but it is still a roadblock. The lack of a README.md also means the user doesn't have run instructions.
2. By default, 'run_pca.py' in the 'bedroc/cc_nc' folder cannot be run because the data folder cannot be found.
3. Assuming the above step is fixed, the wrong float type is configured in '__init__.py' for the version of numpy installed by poetry, which results in a runtime error in 'run_pca.py'.

4. Assuming the above step is fixed, 'run_pca.py' cannot be run because the file 'out/all_idata.pkl' could not be found.

Response: We appreciate the detailed feedback on the various problems with the code. The code has been overhauled and packaged into an easy-to-use open source python package, entitled 'bedroc', now hosted on our GitHub - https://github.com/ExPlanetology/bedroc. The code is now fully documented (readme, example notebooks, and online documentation: https://bedroc.readthedocs.io/en/latest/) and can be installed simply via the `pip install bedroc` command. The source file (NC_CC_AllData.csv) used to produce Fig. 1 and the associated statistical information is also provided within the package. See the 'isotope_anomalies.ipynb' notebook in the notebook/ directory of the source code.

References

C. M. O. Alexander. The origin of inner solar system water. *Philosophical Transactions of the Royal Society A: Mathematical, Physical and Engineering Sciences*, 375 (2094):20150384, 2017.

K. R. Bermingham, H. A. Tornabene, R. J. Walker, L. V. Godfrey, B. S. Meyer, P. Piccoli, and S. Mojzsis. The non-carbonaceous nature of earth's late-stage accretion. *Geochimica et Cosmochimica Acta*, pages 38–51, 2025.

G. Budde, F. L. Tissot, T. Kleine, and R. T. Marquez. Spurious molybdenum isotope anomalies resulting from non-exponential mass fractionation. *Geochemistry*, 83(3): 126007, 2023.

C. Burkhardt, L. Borg, G. Brennecka, Q. Shollenberger, N. Dauphas, and T. Kleine. A nucleosynthetic origin for the earth's anomalous 142nd composition. *Nature*, 537 (7620):394–398, 2016.

N. Dauphas. The isotopic nature of the Earth's accreting material through time. *Nature*, 541(7638):521–524, 2017. ISSN 1476-4687. doi: 10.1038/nature20830.

N. Dauphas, T. Hopp, and D. Nesvorný. Bayesian inference on the isotopic building blocks of Mars and Earth. *Icarus*, 408:115805, Jan. 2024. ISSN 0019-1035. doi: 10.1016/j.icarus.2023.115805.

M. Fischer-Gödde and T. Kleine. Ruthenium isotopic evidence for an inner solar system origin of the late veneer. *Nature*, 541(7638):525–527, 2017.

M. Fischer-Gödde, B.-M. Elfers, C. Münker, K. Szilas, W. D. Maier, N. Messling, T. Morishita, M. Van Kranendonk, and H. Smithies. Ruthenium isotope vestige of earth's pre-late-veneer mantle preserved in archaean rocks. *Nature*, 579(7798): 240–244, 2020.

D. S. Grewal, N. X. Nie, B. Zhang, A. Izidoro, and P. D. Asimow. Accretion of the earliest inner solar system planetesimals beyond the water snowline. *Nature Astronomy*, 8(3):290–297, 2024.

T. S. Kruijer, C. Burkhardt, G. Budde, and T. Kleine. Age of jupiter inferred from the distinct genetics and formation times of meteorites. *Proceedings of the National Academy of Sciences*, 114(26):6712–6716, 2017.

P. M. Kruttasch, K. Ziegler, J.-C. Storck, N. D. Greber, A. Anand, and K. Mezger. Ureilite precursor formation from an isotopically and chemically heterogeneous, isolated protoplanetary disk reservoir. *Earth and Planetary Science Letters*, 652: 119179, 2025.

R. Martins, S. Kuthning, B. J. Coles, K. Kreissig, and M. Rehkämper. Nucleosynthetic isotope anomalies of zinc in meteorites constrain the origin of earth's volatiles. *Science*, 379(6630):369–372, 2023.

B. Marty. The origins and concentrations of water, carbon, nitrogen and noble gases on earth. *Earth and Planetary Science Letters*, 313:56–66, 2012.

B. Marty. Meteoritic noble gas constraints on the origin of terrestrial volatiles. *Icarus*, 381:115020, 2022.

N. Messling, M. Willbold, L. Kallas, T. Elliott, J. G. Fitton, T. Müller, and D. Geist. Ru and w isotope systematics in ocean island basalts reveals core leakage. *Nature*, 642:376–380, 2025.

J. A. Nanne, F. Nimmo, J. N. Cuzzi, and T. Kleine. Origin of the non-carbonaceous–carbonaceous meteorite dichotomy. *Earth and Planetary Science Letters*, 511:44–54, 2019.

F. Nimmo, T. Kleine, A. Morbidelli, and D. Nesvorny. Mechanisms and timing of carbonaceous chondrite delivery to the earth. *Earth and Planetary Science Letters*, 648:119112, 2024.

I. J. Onyett, M. Schiller, G. V. Makhatadze, Z. Deng, A. Johansen, and M. Bizzarro. Silicon isotope constraints on terrestrial planet accretion. *Nature*, 619(7970):539–544, 2023. doi: 10.1038/s41586-023-06135-z.

L. Piani, Y. Marrocchi, T. Rigaudier, L. G. Vacher, D. Thomassin, and B. Marty. Earth's water may have been inherited from material similar to enstatite chondrite meteorites. *Science*, 369(6507):1110–1113, 2020.

J. Render, G. A. Brennecka, C. Burkhardt, and T. Kleine. Solar system evolution and terrestrial planet accretion determined by zr isotopic signatures of meteorites. *Earth and Planetary Science Letters*, 595:117748, 2022.

D. C. Rubie, D. J. Frost, U. Mann, Y. Asahara, F. Nimmo, K. Tsuno, P. Kegler, A. Holzheid, and H. Palme. Heterogeneous accretion, composition and core–mantle differentiation of the earth. *Earth and Planetary Science Letters*, 301(1-2):31–42, 2011.

D. C. Rubie, S. A. Jacobson, A. Morbidelli, D. P. O'Brien, E. D. Young, J. de Vries, F. Nimmo, H. Palme, and D. J. Frost. Accretion and differentiation of the terrestrial planets with implications for the compositions of early-formed solar system bodies and accretion of water. *Icarus*, 248:89–108, 2015.

M. Rüfenacht, P. Morino, Y.-J. Lai, M. A. Fehr, M. K. Haba, and M. Schönbächler. Genetic relationships of solar system bodies based on their nucleosynthetic Ti isotope compositions and sub-structures of the solar protoplanetary disk. *Geochimica et Cosmochimica Acta*, 355:110–125, 2023. ISSN 0016-7037. doi: 10.1016/j.gca.2023.06.005.

M. Schönbächler, R. Carlson, M. Horan, T. Mock, and E. Hauri. Heterogeneous accretion and the moderately volatile element budget of earth. *Science*, 328(5980):884–887, 2010.

Q. R. Shollenberger, J. Render, J. Wimpenny, R. M. Armytage, N. Gunawardena, J. M. Rolison, J. I. Simon, and G. A. Brennecka. Elemental and isotopic signatures of asteroid ryugu support three early solar system reservoirs. *Earth and Planetary Science Letters*, 664:119443, 2025.

E. Siciliano Rego, N. Dauphas, and T. Hopp. Consolidating the isotopic trichotomy of planetary materials with new evidence. *Geochemical Perspectives Letters*, 37:7–11, 2025.

J. Siebert, J. Badro, D. Antonangeli, and F. J. Ryerson. Terrestrial accretion under oxidizing conditions. *Science*, 339(6124):1194–1197, 2013.

H. Tang and N. Dauphas. Abundance, distribution, and origin of 60fe in the solar protoplanetary disk. *Earth and Planetary Science Letters*, 359:248–263, 2012.

P. H. Warren. Stable-isotopic anomalies and the accretionary assemblage of the Earth and Mars: A subordinate role for carbonaceous chondrites. *Earth and Planetary Science Letters*, 311(1):93–100, 2011. ISSN 0012-821X. doi: 10.1016/j.epsl.2011.08.047.

T. Yokoyama, Y. Nagai, R. Fukai, and T. Hirata. Origin and evolution of distinct molybdenum isotopic variabilities within carbonaceous and noncarbonaceous reservoirs. *The Astrophysical Journal*, 883(1):62, 2019.

T. Yoshizaki and W. F. McDonough. Earth and mars–distinct inner solar system products. *Geochemistry*, 81(2):125746, 2021.

J. Zhang, N. Dauphas, A. M. Davis, I. Leya, and A. Fedkin. The proto-earth as a significant source of lunar material. *Nature Geoscience*, 5(4):251–255, 2012.

Homogeneous accretion of the Earth in the inner solar system

Response to Reviewers #3

Legend
Reviewer comments in normal face.
Our responses in bold face. NB: Line numbers refer to the non-track changes version of the manuscript file.

Reviewer - # 2 (Comments for the Author):

Review of:
"Homogeneous accretion of the Earth in the inner solar system"
by Sossi & Bower
Revised manuscript
Overall, the authors have sufficiently-well addressed the points raised in review. I have a few additional comments provided below.

Response: We thank the reviewer for the positive assessment of our work!

Line 62 Please add reference 17 for the NC late veneer constraint.

Response: We have now added the reference in question [Fischer-Gödde and Kleine, 2017] at the appropriate location.

Line 66: How does this fit to your model if 30% of Zn in the BSE derives from CC material?

Response: We show that this is not the case. However, if it turns out that Zn records a higher CC contribution than for the other systems measured (e.g., from newer data collected in future), this might indicate a correlation between the amount of CC material in the BSE and the volatility of the element. This is because Zn is by far the most volatile element for which we have reliable nucleosynthetic isotopic compositions in multiple planetary reservoirs. However, given there are no other moderately volatile elements presented in this work, and no evidence for a higher contribution to Zn compared to other, less volatile elements is found, we prefer not to speculate here.

Lines 237-240: This is not quite correct. Please rephrase this sentence to indicate that the H and N isotope composition of the Earth's surface reservoirs cannot readily be derived from NC-like material alone (see below).

Piani et al. (2020) stated on page 1113 in the last paragraph of their study: "Earth's surficial (oceans and atmosphere) H and N isotopic compositions (deltaD = 0 ‰ and delta15N = 0 ‰) cannot be easily derived from EC materials alone because they are richer in D and 15N by 100 to 200 ‰ and 5 to 40 ‰, respectively." "We find a 4% contribution from CI-like material to Earth's surficial reservoir based on hydrogen abundances and isotopic compositions and a 15% contribution based on equivalent data for nitrogen."

These estimated amounts of CI-like material are not trivial. Therefore, these data refer to a larger fraction of the Earth's accretionary material. Merely stating that the H and N budget could solely be derived from NC meteorite-like material falls too short here.

Response: The reviewer is correct. The isotopic compositions of H and N appear to be more consistent with a CC contribution than for an enstatite chondrite contribution. However, there are three caveats here; *i*) these isotopic systems contain two isotopes only, and hence variations arising from mass-dependent and mass-independent processes cannot be discerned; *ii*) given that the mantle- and surficial (ocean and atmosphere) reservoirs differ in isotopic composition on the same planet (Earth), there is likely to be a mass-dependent control behind such differences and hence they cannot be used in the same way as mass-independent nucleosynthetic variations to infer origin of N and H directly, and *iii*) the D/H ratios of individual meteoritic reservoirs themselves are rather variable (see Grant et al. [2024]), making identification of one particular type of chondrite difficult. Furthermore, we highlight that we do not invoke any of the above chondrites as those that delivered C and N to the Earth - our model would predict that we do not have such samples in the collection. This discussion simply serves to underline that it is ambiguous to use other, non-nucleosynthetic proxies as indicators of origin. The sentence in the main text has now been modified: "Their mantle isotopic compositions, albeit mass-dependent, are also consistent with an NC-like provenance, though their surface reservoirs are fractionated, complicating unambiguous identification of provenance."

General comment about fraction of CC material: There are many studies in which a carbonaceous chondrite contribution among the Earth's building blocks is proposed. Some of these studies also use constraints from isotope compositions (either mass-dependent or mass-independent) of elements in the bulk silicate Earth. Other evidence is based on relative abundances of siderophile and chalcophile elements in the BSE (e.g., Wang and Becker, 2013; Braukmüller et al., 2019; Mezger et al., 2021).

It is fine if with your model you arrive at a different probably more provoking conclusion, but I think it is good scientific conduct to also refer to the constraints

from prior studies and compare them with your new results. Reading through the manuscript I sometimes feel that this could be done more properly, e.g. by adding a sentence in the conclusion section that opposed to the new model presented by the authors, previous studies based on X elements or Y isotopes provided evidence for accretion of CC material.

I will leave this point to the authors about how they want their paper to be perceived by the community.

Response: Point taken. There have indeed been a multitude of studies that use other, non-nucleosynthetic proxies as supporting the accretion of CC material to the Earth, as cited by the reviewer. As we discuss above and in the previous response to reviewers, these proxies are likely to be less exacting as to tracing the origin of materials that accreted to Earth than are nucleosynthetic isotopic variations. We agree with the reviewer that it is worth mentioning the body of evidence used in support of such a notion. We feel this statement is better placed within the introduction, and we add a sentence to this end that reads: "Proxies that are not nucleosynthetic in origin [Schönbächler et al., 2010; Wang and Becker, 2013; Alexander, 2017; Varas-Reus et al., 2019], have been cited in support of a CC contribution to the BSE, particularly in the latter stages of its accretion."

Other than that, I think the revised version of the manuscript is much improved and after thorough consideration of the above-mentioned points is well-suited for publication in Nature Astronomy.

Response: Thank you for your careful and detailed reviews!

Cologne, January 2026
Mario Fischer-Gödde

References

C. M. O. Alexander. The origin of inner solar system water. *Philos. Trans. R. Soc. A Math. Phys. Eng. Sci.*, 375(2094):20150384, 2017.

M. Fischer-Gödde and T. Kleine. Ruthenium isotopic evidence for an inner Solar System origin of the late veneer. *Nature*, 541(7638):525–527, 2017.

H. Grant, R. Tartèse, R. Jones, L. Piani, and Y. Marrocchi. Identification of a primordial high d/h component in the matrix of unequilibrated ordinary chondrites. *Geochimica et Cosmochimica Acta*, 378:58–70, 2024.

M. Schönbächler, R. Carlson, M. Horan, T. Mock, and E. Hauri. Heterogeneous accretion and the moderately volatile element budget of Earth. *Science*, 328(5980): 884–887, 2010.

M. I. Varas-Reus, S. König, A. Yierpan, J.-P. Lorand, and R. Schoenberg. Selenium isotopes as tracers of a late volatile contribution to Earth from the outer Solar System. *Nature Geosci.*, 12(9):779–782, 2019.

Z. Wang and H. Becker. Ratios of S, Se and Te in the silicate Earth require a volatile-rich late veneer. *Nature*, 499(7458):328–331, 2013.

Sossi and Bower (review)

Reviewed by Greg Brennecka (w/ code checking help from someone in the group).

Summary: This is a great paper overall. I have very few criticisms of the work, and I commend the authors on a very nice study. My comments are basically just ways I think the authors can clarify a few things for the general reader and some minor suggestions. Very good stuff and, other than all the math, a pleasure to read :)

Specifics:

Line 26: There is no doubt that the Trinquier et al. 2009 paper is a seminal work in cosmochemistry, but I don't think it is the correct reference here. The NC/CC dichotomy, to my reading, wasn't really recognized in print until Warren in 2011. Following that paper, there are myriad refs you could use, such as works like Budde et al. 2016, Kruijer et al. 2017, and perhaps the review by Bermingham et al. 2020 to direct people to other work/readings on the subject. Trinquier et al. 2009 deserves a ton of credit, but probably not on the dichotomy in my opinion, despite what the authors might now claim they wrote about in that important 2009 paper.

Line ~55: I think the discussion of the percent of CC material making up Earth is good and needed, but they way it is currently written could be misconstrued that 40% of the *mass* of Earth, when, it is of course 40% of the sampled Mo is from CC – because the rest is inaccessible and in the core. I am certain the authors understand this, and it is worded properly as they do (L55: "the fraction of Mo derived…"), but given this is a general interest journal, I am not sure it will come through to folks not fully immersed in the topic. I would suggest it could help to make this clearer what is actually being claimed by the Mo, Zn, et al. studies. Again, this is not a knock on the author's current phrasing or anything, just a suggestion to make it more understandable to those that are not experts in the topic already.

Line 140: Perhaps consider including Shollenberger et al. (2025-EPSL) and Rego et al. (2025-GPL) here, they come to similar conclusions based on isotopes from major elements from Ryugu and other CIs. The Rego paper was likely published after submission of your manuscript, but both papers provide strong support and discussion for a separate CI/Ryugu reservoir. Not critical, but would be helpful for the argument here.

Figure 1 and associated: No agenda or important question here, but I am curious why the authors prioritized ^{64}Ni over ^{62}Ni or ^{60}Ni. All show variation, and ^{60}Ni can behave differently than 64 (or 62, which are usually well correlated). And since ^{94}Mo and ^{95}Mo were both chosen, I was just curious as to why only one Ni isotope was included. Something to consider, and it might change Ni a bit if 60 is used instead/in addition. Of course the decay

and input from ^{60}Fe might need to be considered in some cases, but something to think about.

Fig. 1 and associated: Very interesting about the ureilites...is it worth some more discussion as to why this is the case? If you are positing the CI are a separate reservoir, then it would stand to reason that the ureilites would be a fourth, right? This is of course addressed in the nice work by Rufenacht et al. 2023, but without the statistical rigor you are presenting here. I would consider expanding on this a bit, perhaps.

Fig. 2: Just a thought, but perhaps it would be easier to see if you also used different shades of gray/green in addition to circle square. Cool figure, but not easy to read at first glance and I think that will help. Also, most of the values are BARELY within uncertainty (e.g., Ni, Zn, Ca, Zr?, Fe?), so this is not a slam dunk, but cool, no doubt.

Line 274: I don't understand what "1" points to here.

Code check:

It was asked to check the code, so here are some notes about the downloadable files specifically:

There are four folders that were provided in the raw materials. A description of run issues is provided below for each of the four folders.

10634_0_source_data_130915_t11b23/

This folder only contains data and does not have any code to run.

10634_0_source_data_4713666_stl3bb/

Assuming the user knows how to set up the proper environment, this code runs fine. Two plots are produced, but only one is saved. The unsaved figure looks different from what's in the supplied manuscript, but effort was only put into execution, not reproduction.

10634_0_source_data_4713667_stl3bc/

Assuming the user knows how to set up the proper environment, this code runs fine. The figure mostly matches the one in the supplied manuscript.

code_pca/

The code in this folder could not be run.

1. No README.md file was provided with the code, which means the code cannot be installed properly using Poetry. This was easily fixed, but it is still a roadblock. The lack of a README.md also means the user doesn't have run instructions.
2. By default, `run_pca.py` in the `bedroc/cc_nc` folder cannot be run because the data folder cannot be found.
3. Assuming the above step is fixed, the wrong float type is configured in `__init__.py` for the version of numpy installed by poetry, which results in a runtime error in `run_pca.py`.
4. Assuming the above step is fixed, `run_pca.py` cannot be run because the file `out/all_idata.pkl` could not be found.
